# Reshaping Ullmann Amine Synthesis in Deep Eutectic Solvents: A Mild Approach for Cu-Catalyzed C–N Coupling Reactions With No Additional Ligands

**DOI:** 10.3389/fchem.2019.00723

**Published:** 2019-10-30

**Authors:** Andrea Francesca Quivelli, Paola Vitale, Filippo Maria Perna, Vito Capriati

**Affiliations:** Dipartimento di Farmacia-Scienze del Farmaco, Università di Bari “Aldo Moro”, Consorzio C.I.N.M.P.I.S., Bari, Italy

**Keywords:** Ullmann amination reaction, copper catalysis, deep eutectic solvents, amine synthesis, green chemistry

## Abstract

The CuI-catalyzed Ullmann amine cross-coupling between (hetero)aryl halides (Br, I) and aromatic and aliphatic amines has been accomplished in deep eutectic solvents as environmentally benign and recycling reaction media. Under optimized conditions, the reaction proceeds smoothly under mild conditions (60–100°C) in air, in the absence of ligands, with a catalyst (CuI) loading of 10 mol% and K_2_CO_3_ (aliphatic primary and secondary amines) or *t*-BuOK (aromatic amines) as the base. A variety of amines have been synthesized in yields up to 98% with a broad substrate scope.

## Introduction

The century “classical” Ullmann reaction is known to be the first transition metal-mediated organic transformation, and it was used for the synthesis of symmetric biaryls from aryl halides (Ullmann, 1901, 1903, 1904; Goldberg, 1906). However, as it suffered from the requirement of stoichiometric amounts of copper salts, harsh reaction conditions (temperatures ≥200°C), poor functional group tolerance and low yields of the expected adducts, it remained dormant for decades with limited applications. As a result of the tremendous impact of Pd-catalyzed cross-coupling reactions have had in the field of organic synthesis over the past 25 years (Seechurn et al., 2012; Sherwood et al., 2019), there has been recently a revival of interest in the so-called “Ullmann-type” reaction, which makes use of cheaper and environmentally responsible first-row transition metals to catalyze the nucleophilic aromatic substitution between an aryl halide and a nucleophile (Cristau et al., 2004, 2005; Chen and Chen, 2006; Shafir and Buchwald, 2006; Jones et al., 2007; Ma and Cai, 2008; Tye et al., 2008; Beletskaya and Ananikov, 2011; Sambiagio et al., 2014). The fundamental interrogation of how this reaction proceeds (radical or polar mechanism) has been a matter of debate throughout the years, and details still remain challenging today. A mechanism involving a Cu(I)/Cu(III) catalytic cycle has been experimentally supported in most Ullmann-type couplings, whereas the one-electron redox pathway involving aryl radical species is thought to take place in certain C-Heteroatom coupling reactions under light irradiation (Strieter et al., 2005; Tye et al., 2008; Giri and Hartwig, 2010; Sperotto et al., 2010; Casitas and Ribas, 2013; Ribas and Güell, 2014).

**Scheme 1:**
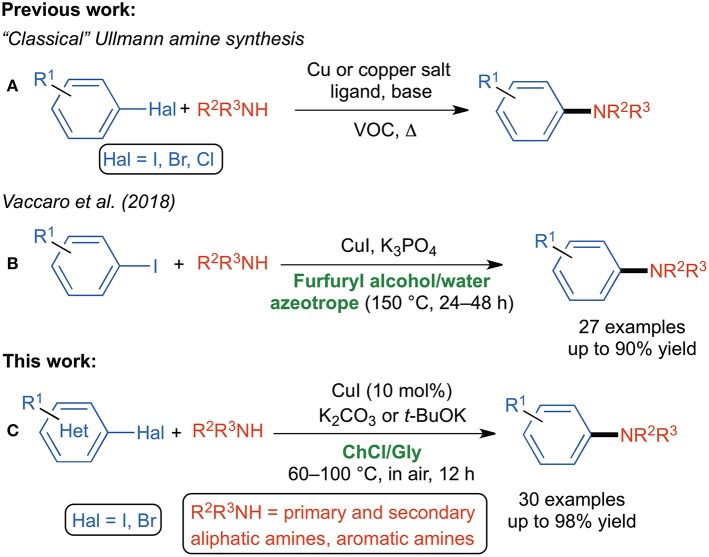
**(A)** “Classical” Ullmann amine synthesis; **(B)** bio-based Ullmann amine synthesis; **(C)** DES-based Ullmann amine synthesis.

Among *N*-arylation strategies available in the armory of the synthetic chemist, the Ullmann amine synthesis (UAS) has sparked in recent years the interest of the scientific community as it well complements the most famous Pd-catalyzed Hartwig-Buchwald amination reaction (Hartwig, 1998, 2000; Wolfe et al., 1998; De Meijere and Diederich, 2004; Surry and Buchwald, 2008). Key aspects of the UAS are the use of (a) polydentate auxiliary ligands with oxygen- or nitrogen coordination sites (e.g., diamines, amino acids, oximes, diols, 1,10-phenantrolines), which often improve the solubility of the copper precursors and the stability of the active catalyst, and/or (b) toxic and volatile organic compounds (VOCs) (e.g., DMF, DMSO, dioxane, THF, toluene) (Scheme 1A) (Evano et al., 2008; Monnier and Taillefer, 2009; Surry and Buchwald, 2010; Zhang et al., 2012b; Jiang et al., 2013; Zhou et al., 2015; Gao et al., 2017). Copper-catalyzed *N*-arylation in water or in aqueous media are also known. However, they are usually carried out in the presence of phase transfer catalysts and with ligands, and often require high reaction temperature (100–130°C) (Li et al., 2010; Huang et al., 2013; Sambiagio et al., 2014; Chakraborti et al., 2018). Interestingly, Wei et al. have reported in 2011 the copper powder-catalyzed ligand-free amination of aryl halides with aliphatic amines in neat water and in air at 100°C, but secondary amines and aniline derivatives proved to be not reactive under these conditions (Jiao et al., 2011). Recent publications have highlighted the successful use of (engineered) surfactants (a) as ligands to stabilize the copper intermediates in the catalytic transformation and (b) to provide a micellar environment favoring the aggregation of reagents in water (Liu and Zhou, 2013; Ge et al., 2019). Green pathways to biaryls via palladium nanoparticles-catalyzed Ullmann reactions in ionic liquids or in ionic liquids/supercritical carbon dioxide systems have also been reported (Durán Pachón et al., 2006; Calò et al., 2009; Cheng et al., 2010).

Growing efforts are continuously being made to replace harmful, carcinogenic, petroleum-based VOCs with “greener,” recyclable neoteric reaction media, as the largest contribution to the waste stream (over 80%) in the practice of synthetic organic chemistry is provided by solvents (Lipshutz and Ghorai, 2014; Lipshutz et al., 2016). As for the Ullmann-type reaction, an important step forward has recently been made by Vaccaro et al. who developed a waste-minimized protocol for copper-catalyzed UAS in the biomass-derived furfuryl alcohol able to act as an effective bidentate ligand for copper and also to form an azeotrope with water (Scheme 1B) (Ferlin et al., 2018). Deep Eutectic Solvents (DESs) are mixtures usually made up of a hydrogen bond donor (HBD) and a hydrogen bond acceptor (HBA) that, upon mixing together in a proper molar ratio and heating, show an eutectic point temperature far below that of an ideal liquid mixture because of strong hydrogen-bonding interactions between the individual components that decreases the lattice energy of the system. DESs have been defined as the organic reaction medium of the twenty-first century and provide a valuable “green” alternative to common VOCs because of some interesting properties: a low vapor pressure, thermal stability, non-flammability and a high heat capacity (Ruß and König, 2012; Zhang et al., 2012a; García-Álvarez, 2015; García-Álvarez et al., 2015, 2018; Liu et al., 2015; Alonso et al., 2016; Sheldon, 2016; Martins et al., 2018). There is also a consensus that the toxicity and citotoxicity of hydrophilic DESs depend on their components (Hayyan et al., 2013, 2015, 2016). Those based on naturally occurring and biomass-produced compounds [e.g., amino acids, sugars, vitamins, choline chloride (ChCl)] benefit of tunable physicochemical properties, biodegradability, easy preparation, non-toxicity and low cost (Hayyan et al., 2013). To date, *N*-arylation of amines in DESs has only been performed employing an engineered copper nanoparticle modified carboxamide-functionalized magnetic graphene oxide as an efficient nanocatalyst. The latter was recovered and reused, jointly with the eutectic mixture, for five consecutive runs (Shaabani and Afshari, 2018). *N*-arylantraquinone derivatives have also been successfully synthesized, under metal-free conditions and in the presence of choline hydroxide as an environmentally benign and recyclable catalyst, however, via an S_N_Ar mechanistic pathway (Pant et al., 2018). Building on recent breakthroughs made by our research group (Mallardo et al., 2014; Sassone et al., 2015; Cicco et al., 2016, 2017, 2018; Mancuso et al., 2016; Dilauro et al., 2017, 2018, 2019; Messa et al., 2018; Ghinato et al., 2019) and others (Vidal et al., 2014, 2016; Sánchez-Condado et al., 2019) on the effectiveness of using non-conventional reaction media (e.g., DESs and water) for promoting metal-catalyzed and organolithium/Grignard reagent-mediated deprotonation and nucleophilic addition reactions to unsaturated organic substrates under aerobic conditions, here we report the CuI-catalyzed UAS in DESs. The following features of the proposed protocol are noteworthy: (a) the transformation takes place smoothly in a ChCl/glycerol (Gly) eutectic mixture under air and moderate heating (60–100°C), (b) commercially available (hetero)aryl bromides and iodides are employed, (c) the reaction proceeds with no additional ligands and with a broad substrate scope, (d) the expected adducts are isolated in yields up to 98%, and (e) the catalyst, the DES and the base were easily and successfully recycled up to six times (Scheme 1C).

## Results and Discussion

**Figure 1:**
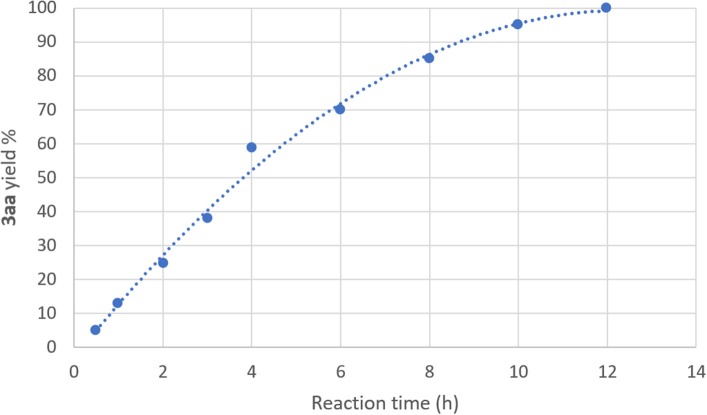
Yield vs. time profile for the synthesis of **3aa** at 60°C, in air. The yields were determined by ^1^H NMR using CH_2_Br_2_ as the internal standard.

**Scheme 2:**
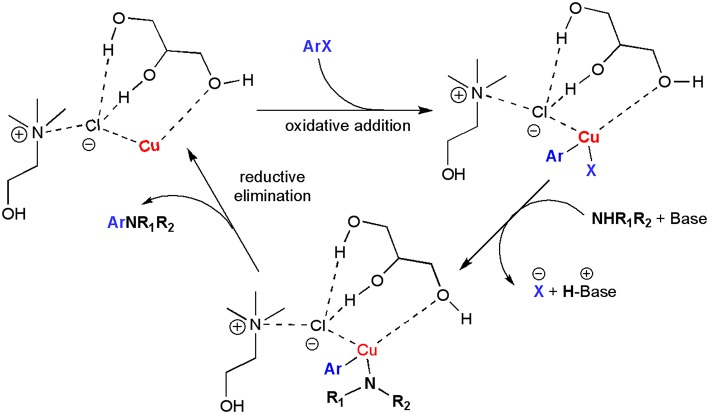
Possible catalytic cycle for the UAS promoted by DES.

As DESs are known to be used not only as solvents, but also as catalytic active species, batch reactions to assess the ability of DESs to promote the UAS with no additional ligands were carried out. We initially investigated as a model reaction the coupling between bromobenzene **1a** (0.5 mmol) and *N, N*-dimethylethylenediamine **2a** (1 equiv) in different eutectic mixtures for the preparation of adduct **3aa** using CuI (10 mol%) as a catalyst and K_2_CO_3_ as a base (Table 1). Since the beginning, encouraging results were achieved using DESs as reaction media for this kind of reaction. The mixture ChCl/Gly (1:2 mol mol^−1^) was the best among those tested as it delivered adduct **3aa** in 98% yield after 12 h heating and stirring at 60°C in air (entries 1–4, Table 1). The reaction is characterized by a slow kinetics delivering adduct **3aa** in a lower yield at shorter reaction time than 12 h (Figure 1). On the other hand, no coupling was observed by running the reaction at room temperature, in the absence of the base or CuI, whereas the yield of **3aa** dropped to 20–60% with a catalyst loading inbetween 5–7 mol% (entries 5–9, Table 1). A screening of bases also revealed that K_2_CO_3_, KOH and *t*-BuOK were equally effective when used in 2 equiv (entries 10–12, Table 1). Unsatisfactory outcomes in terms of reaction yield (5–30%) were instead obtained when using water, the ChCl/H_2_O (1:2 mol mol^−1^) eutectic mixture or Gly as the solvent (entries 13–15, Table 1). As has been discussed by Ma in an *Account* (Ma and Cai, 2008), UAS is generally carried out in the presence of bidentate ligands (among which amino acids) which might make Cu(I) species more reactive toward the oxidative addition reaction or stabilize the oxidative addition intermediates, thereby boosting the coupling process. The fact that the above-described UAS takes place smoothly in DESs in the absence of additional ligands points toward a somewhat stabilizing effect exerted by certain eutectic mixtures on the copper salt, eventually resulting in an improved catalytic performance (Scheme 2).

**Table 1:** Optimization of Ullmann amine coupling reaction between bromobenzene **1a** and *N, N*-dimethylethylenediamine **2a** to give adduct **3aa**^a^.

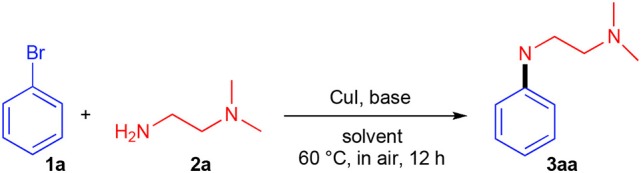				
**Entry**	**Solvent**	**CuI (mol%)**	**Base (equiv)**	**Yield (%)^b^**
1	DES A	10	K_2_CO_3_ (2)	20
2	DES B	10	K_2_CO_3_ (2)	82
3	DES C	10	K_2_CO_3_ (2)	91
**4**	**DES D**	**10**	**K**_**2**_**CO**_**3**_ **(2)**	**98^c^**
5	DES D^d^	10	K_2_CO_3_ (2)	0
6	DES D	10	–	0
7	DES D	–	K_2_CO_3_ (2)	0
8	DES D	5	K_2_CO_3_ (2)	20
9	DES D	7	K_2_CO_3_ (2)	60
10	DES D	10	K_2_CO_3_ (1)	50
11	DES D	10	KOH (2)	97
12	DES D	10	*t*-BuOK (2)	97
13	H_2_O	10	K_2_CO_3_ (2)	5
14	DES E	10	K_2_CO_3_ (2)	5
15	glycerol	10	K_2_CO_3_ (2)	30

a *Reaction conditions: bromobenzene (0.5 mmol), N,N-dimethylethylenediamine (1.0 equiv), CuI, and base were suspended in 1.0 g DES or in 1.0 mL water or glycerol and stirred vigorously at 60°C for 12 h. DES A, L-proline/L-lactic acid (1:2 mol mol^−1^); DES B, choline chloride/urea (1:2 mol mol^−1^); DES C, L-proline/glycerol (2:5 mol mol^−1^); DES D, choline chloride/glycerol (1:2 mol mol^−1^); DES E, choline chloride/water (1:2 mol mol^−1^)*.

b *Calculated via ^1^H NMR analysis of the crude reaction mixture using an internal standard technique (NMR internal standard: CH_2_Br_2_)*.

c *Yield of isolated product*.

d *Room temperature*.

**Scheme 3:**
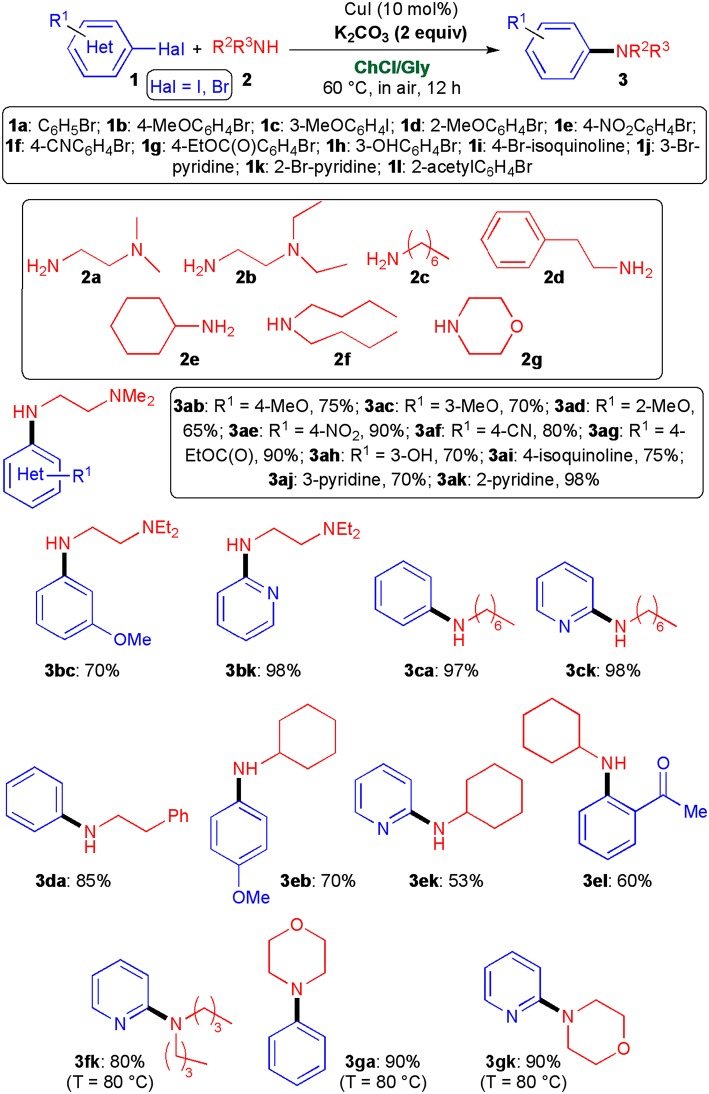
Synthesis of functionalized secondary and tertiary amines **3** by copper-catalyzed cross-coupling reactions of (hetero)aryl halides **1** with amines **2**. The yields reported are for products isolated and purified by column chromatography.

Adopting the optimized conditions described in Table 1, entry 4, we then explored the scope of this cross-coupling reaction with a variety of aliphatic primary and secondary amines (**2a**–**g**) and (hetero)aryl halides (**1a**–**l**) (Scheme 3). With regard to diamines **2a**, **b** and primary amines **2c**–**e** relatively high yields (53–98%) of the desired coupled products (**3ab**–**3ak**, **3bc, 3bk**, **3ca**, **3ck**, **3da**, **3eb**, **3ek**, and **3el**) were obtained not only in the reaction with aryl iodides and bromides bearing electron-donating (hydroxy, methoxy) and electron-withdrawing (ester, nitro, cyano, keto) groups at the *ortho*-, *meta*-, and *para*-positions, but also with brominated heterocycles, such as 2- and 3-bromopyridine and 4-bromoisoquinoline. Interestingly, both aliphatic and heterocyclic secondary amines **2f**, **g** proved to be competent coupling partners as well, and afforded (hetero)aryl derivatives **3fk**, **3ga**, and **3gk** in 80–90% yield after simply heating the reaction mixture from 60 to 80°C.

**Scheme 4:**
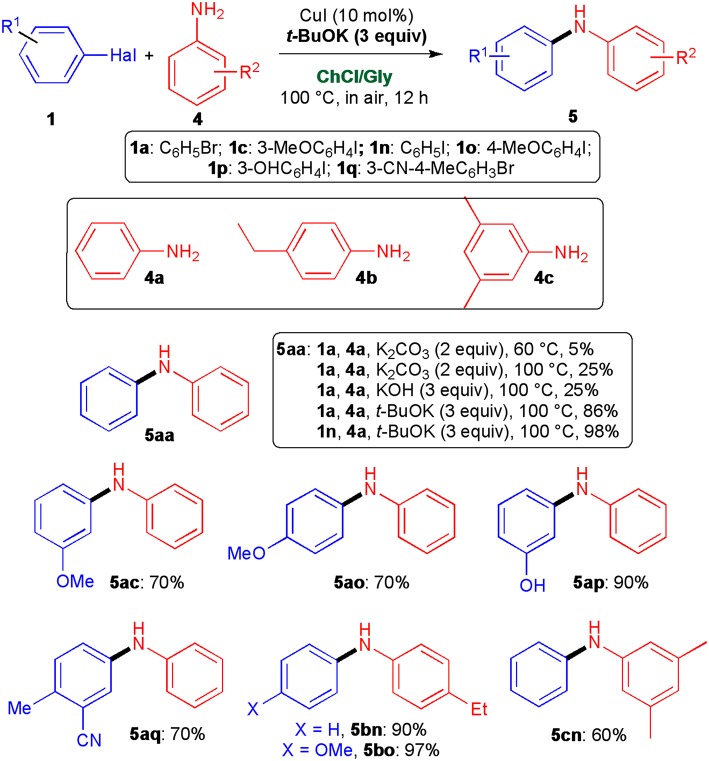
Synthesis of diarylamines **5** by copper-catalyzed cross-coupling reactions of aryl halides **1** with arylamines **4**. The yields reported are for products isolated and purified by column chromatography.

To further explore the utility of this new protocol, we also investigated the synthesis of diarylamines, which are described in the literature as important molecules due to their antioxidant capacity (Pinto-Basto et al., 2009). Preliminary experiments disclosed that, under the above experimental conditions, aniline (**4a**) hardly coupled with bromobenzene (**1a**) as the desired adduct **5aa** was delivered in 5% yield only. Even by increasing the temperature to 100°C or by replacing K_2_CO_3_ with the stronger KOH (3 equiv) the **5aa** yield was not higher than 25% (Scheme 4). To our delight, when the UAS was conducted in the presence of 3 equiv of *t*-BuOK at 100°C, adduct **5aa** now formed in 86% yield. This percentage could be improved up to 98% if iodobenzene (**1n**) was alternatively used as the starting aryl halide. Cross-couplings of **4a** and aniline derivatives **4b**, **c** with **1n** and assorted aryl iodides and bromides substituted with a methoxy (**1c**, **1o**), a hydroxy (**1p**), and a cyano group (**1q**) proceeded uneventfully as well in the presence of *t*-BuOK, at 100°C in air, leading to functionalised diarylamines **5ac**, **5ao**, **5ap**, **5aq**, **5bn**, **5bo**, and **5cn** in 60–97% yield (Scheme 4). Of note, under the above conditions, the coupling between **2a** and aryl chlorides, such as chlorobenzene and 3-chloropyridine proved to be ineffective even after 24 h reaction time.

**Figure 2:**
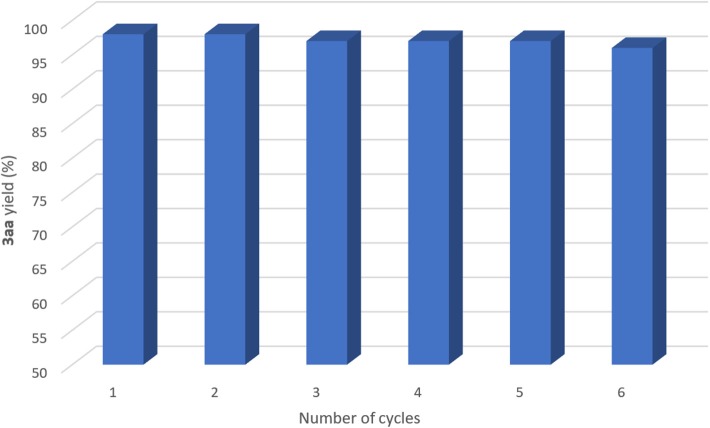
Recycling of CuI, DES, and K_2_CO_3_ in the coupling reaction between bromobenzene (**1a**) and *N, N*-dimethylethylenediamine (**2a**) with 10 mol% catalyst. The yields were determined by ^1^H NMR using CH_2_Br_2_ as the internal standard.

The catalyst, the DES and the base could be recycled easily. The amination reaction of bromobenzene **1a** with diamine **2a** was chosen as a model reaction as it provided almost quantitative yield (98%) of the corresponding adduct **3aa** (entry 4, Table 1). Once the stirring was stopped after 12 h reaction time, in-flask extraction with cyclopentyl methyl ether (CPME) (Watanabe et al., 2007; Azzena et al., 2019) (1 mL), which is an environmentally responsible solvent, afforded product **3aa** (98% yield, determined by ^1^H NMR, Figure 2, number of cycles = 1), but leaving the active copper species in the eutectic mixture. Then, upon the addition of new, fresh reagents, the catalyst, the DES and the base could be successfully re-used for further reaction runs. As shown in Figure 2, the catalyst remained active over 6 cycles, with a decrease in the final yield of **3aa** of up to 2%: 98% (second run), 97% (third run), 97% (fourth run), 97% (fifth run), and 96% (sixth run). In order to compare the reaction rates observed for each run, we re-investigated the above catalytic cycle at a lower conversion. Thus, each run was stopped after 3 h reaction time and the corresponding yield of **3aa** calculated: 38% (first, second, and third run), 36% (fourth run), 38% (fifth and sixth run), and 36% (seventh run). Overall, these results confirmed the excellent performance of the copper species as catalyst in the employed eutectic mixture. Finally, to evaluate the sustainability of the proposed protocol and to provide metrics for the “green” aspects herein discussed, we calculated the E-factor (kg of waste/kg of product) (Sheldon, 2007; Roschangar et al., 2015) for the recycling process, obtaining a value as low as 13.8 (10 mol% CuI, 6 cycles, see Supplementary Material for details).

## Conclusion

In summary, a practical and efficient protocol has been developed for performing CuI-catalyzed Ullmann amine synthesis using a biodegradable ChCl-based eutectic mixture as an environmentally responsible reaction medium in place of toxic and hazardous VOCs. Valuable aspects of the proposed protocol are: (a) cross-coupling reactions between (hetero)aryl bromides and iodides and a variety of aromatic and aliphatic primary and secondary amines run under mild conditions (60–100°C) in air, with K_2_CO_3_ or *t*-BuOK as the base, and a catalyst loading of CuI of up to 10 mol%; (b) the absence of additional ligands; (c) good reaction efficiency starting from both electron-deficient and electron-rich (hetero)aryl halides; (d) broad substrate scope and tolerance of several functional groups with the expected adducts isolated in up to 98% yield; (e) effective recycling of the DES, the catalyst and the base. Efforts toward targeting and producing medicinally relevant drug compounds on a larger scale are underway and will be reported in due course.

## Materials and Methods

### General Methods

^1^H NMR and ^13^C NMR spectra were recorded on a Bruker 600 MHz spectrometer and chemical shifts are reported in parts per million (δ). FT-IR spectra were recorded on a Perkin-Elmer 681 spectrometer. GC analyses were performed on a HP 6890 model, Series II by using a HP1 column (methyl siloxane; 30 m × 0.32 mm × 0.25 μm film thickness). Analytical thin-layer chromatography (TLC) was carried out on pre-coated 0.25 mm thick plates of Kieselgel 60 F254; visualization was accomplished by UV light (254 nm) or by spraying a solution of 5% (w/v) ammonium molybdate and 0.2% (w/v) cerium(III) sulfate in 100 mL 17.6% (w/v) aq. sulfuric acid and heating to 473 K until blue spots appeared. Chromatography was conducted by using silica gel 60 with a particle size distribution 40–63 μm and 230–400 ASTM. GC-MS analyses were performed on HP 5995C model. Cyclopentyl methyl ether (CPME) was used as the solvent in the work-up procedures. High-resolution mass spectrometry (HRMS) analyses were performed using a Bruker microTOF QII mass spectrometer equipped with an electrospray ion source (ESI). CP-OES analysis was performed on a Spectro Arcos FHE 12 (Ametek) spectrometer. Reagents and solvents, unless otherwise specified, were purchased from Sigma-Aldrich (Sigma-Aldrich, St. Louis, MO, USA) and used without any further purification. (Hetero)aryl halides and amines used for the synthesis of compounds **3** and **5** are commercially available. Copies of ^1^H and ^13^C NMR spectra are available in the Supplementary Material. Deep Eutectic Solvents [DES A: L-proline/L-lactic acid (1:2 mol mol^−1^); DES B: choline chloride/urea (1:2 mol mol^−1^); DES C: L-proline/glycerol (2:5 mol mol^−1^); DES D: choline chloride/glycerol (1:2 mol mol^−1^); DES E: choline chloride/water (1:2 mol mol^−1^)] were prepared by heating under stirring at 60–80°C for 10–30 min the corresponding individual components until a clear solution was obtained.

#### Typical Procedure for the Ullmann Cross-Coupling Reaction With Aliphatic Amines: Synthesis of *N*^1^,*N*^1^-dimethyl-*N*^2^-phenylethane-1,2-diamine (3aa)

CuI (10 mol%, 0.05 mmol, 10 mg), bromobenzene (**1a**, 1 equiv, 0.5 mmol, 79 mg, 53 μL), *N, N*-dimethylethylenediamine (**2a**, 1 equiv, 0.5 mmol, 44 mg, 55 μL) and the base (K_2_CO_3_, 2 equiv, 1 mmol, 138 mg) were suspended in 1 g DES (Gly/ChCl, 2:1 mol mol^−1^), under air, in a vial with a Teflon screw tap under vigorous stirring at 60°C. The reaction mixture was monitored by GC. After 12 h, the mixture was cooled to room temperature and 1 mL of H_2_O was added. Then, the mixture was extracted with CPME (3 × 1 mL) and the organic phase was dried over anhydrous Na_2_SO_4_ and filtered over a celite pad. Evaporation of the solvent under reduced pressure afforded the crude that was purified by flash-chromatography on silica gel (hexane/AcOEt 8:2) to provide the desired product **3aa** in 98% yield (80 mg).

#### *N*^1^,*N*^1^-Dimethyl-*N*^2^-phenylethane-1,2-diamine (3aa)

Colorless oil, yield 98%.

^1^H NMR (600 MHz, CDCl_3_) δ 2.27 (s, 6 H), 2.56–2.58 (m, 2 H), 3.16–3.17 (m, 2 H), 6.65 (d, *J* = 7.2 Hz, 2 H), 6.72 (t, *J* = 7.2 Hz, 1 H), 7.20 (d, *J* = 7.2 Hz, 2 H).

^13^C NMR (150 MHz, CDCl_3_) δ 42.2, 45.2, 58.1, 112.9, 117.2, 129.2, 148.6.

FT-IR (film, cm^−1^): 3379, 3051, 2920, 2821, 1728, 1603, 1505, 1463, 1258, 1041, 748, 700.

GC/MS (70 eV) *m/z* (%): 164 (M^+^, 15), 106 (10), 77 (9), 58 (100), 42 (6).

HRMS (ESI) *m/z* calcd for [C_10_H_16_N_2_ + H]^+^: 165.1386; found: 165.1393.

#### *N*^1^-(4-Methoxyphenyl)-*N*^2^,*N*^2^-dimethylethane-1,2-diamine (3ab)

Colorless oil, yield 75%.

^1^H NMR (600 MHz, CDCl_3_) δ 2.26 (s, 6 H), 2.56 (t, *J* = 6.0 Hz, 2 H), 3.12 (t, *J* = 6.0 Hz, 2 H), 3.76 (s, 3 H), 6.62 (d, *J* = 7.6 Hz, 2 H), 6.80 (d, *J* = 7.6 Hz, 2 H).

^13^C NMR (150 MHz, CDCl_3_) δ 42.2, 45.3, 55.9, 58.2, 114.2, 114.9, 143.0, 152.1.

FT-IR (film, cm^−1^): 3368, 2942, 1619, 1514, 1464, 1288, 1243, 1179, 1039, 959, 820.

GC/MS (70 eV) *m/z* (%): 194 (M^+^, 35), 136 (42), 135 (18), 121 (61), 108 (59), 77 (5), 59 (9), 58 (100), 42 (6).

HRMS (ESI) *m/z* calcd for [C_11_H_18_N_2_O + H]^+^: 195.1492; found: 195.1495.

#### *N*^1^-(3-Methoxyphenyl)-*N*^2^,*N*^2^-dimethylethane-1,2-diamine (3ac)

Colorless oil, yield 70%.

^1^H NMR (600 MHz, CDCl_3_) δ 2.24 (s, 6 H), 2.54 (t, *J* = 5.9 Hz, 2 H), 3.12 (t, *J* = 5.9 Hz, 2 H), 3.75 (s, 3 H), 6.18 (s, 1 H), 6.23–6.26 (m, 2 H), 7.06 (t, *J* = 8.0 Hz, 1 H).

^13^C NMR (150 MHz, CDCl_3_) δ 41.1, 45.1, 55.0, 58.0, 98.7, 102.4, 106.0, 129.9, 149.9, 160.8.

FT-IR (film, cm^−1^): 3400, 2924, 2852, 1736, 1602, 1516, 1457, 1378, 1346, 1252, 1179, 1128, 1026, 916, 737.

GC/MS (70 eV) *m/z* (%): 194 (M^+^, 30), 136 (36), 135 (16), 92 (5), 59 (9), 58 (100), 42 (6).

HRMS (ESI) *m/z* calcd for [C_11_H_18_N_2_O + H]^+^: 195.1492; found: 195.1493.

#### *N*^1^-(2-Methoxyphenyl)-N^2^,N^2^-dimethylethane-1,2-diamine (3ad)

Colorless oil, yield 65%.

^1^H NMR (600 MHz, CDCl_3_) δ 2.29 (s, 6 H), 2.61 (t, *J* = 6.2 Hz, 2 H), 3.22 (t, *J* = 6.2 Hz, 2 H), 3.86 (s, 3 H), 6.61 (d, *J* = 7.6 Hz, 1 H), 6.67 (t, *J* = 7.6 Hz, 1 H), 6.77 (d, *J* = 7.6 Hz, 1 H), 6.88 (t, *J* = 7.6 Hz, 1 H).

^13^C NMR (150 MHz, CDCl_3_) δ 41.2, 45.3, 55.4, 58.3, 109.4, 109.8, 116.4, 121.2, 138.4, 147.1.

FT-IR (film, cm^−1^): 3401, 2926, 2819, 2768, 1602, 1519, 1238, 1130, 733.

GC/MS (70 eV) *m/z* (%): 194 (M^+^, 26), 136 (28), 121 (16), 120 (23), 77 (6), 65 (6), 58 (100), 42(8). HRMS (ESI) *m/z* calcd for [C_11_H_18_N_2_O + H]^+^: 195.1492; found: 195.1493.

#### *N*^1^,*N*^1^-Dimethyl-*N*^2^-(4-nitrophenyl)ethane-1,2-diamine (3ae)

Yellow oil, yield 90%.

^1^H NMR (600 MHz, CDCl_3_) δ 2.26 (s, 6 H), 2.54–2.57 (m, 2 H), 3.15–3.16 (m, 2 H), 4.94 (br. s, 1 H), 6.56 (d, *J* = 7.6 Hz, 2 H), 7.42 (d, *J* = 7.6 Hz, 2 H).

^13^C NMR (150 MHz, CDCl_3_) δ 40.0, 44.8, 57.1, 111.1, 130.0, 137.9, 153.3.

FT-IR (film, cm^−1^): 3369, 2920, 1601, 1466, 1307, 1111, 838.

GC/MS (70 eV) *m/z* (%): 209 (M^+^, 22), 120 (5), 76 (6), 58 (100), 51 (8), 42 (7).

HRMS (ESI) *m/z* calcd for [C_10_H_15_N_3_O_2_ + H]^+^: 210.1237; found: 210.1239.

#### 4-{[2-(Dimethylamino)ethyl]amino}benzonitrile (3af)

Colorless oil, yield 80%.

^1^H NMR (600 MHz, CDCl_3_) δ 2.27 (s, 6 H), 2.57–2.59 (m, 2 H), 3.15–3.18 (m, 2 H), 4.97 (br. s, 1 H), 6.56 (d, *J* = 7.6 Hz, 2 H), 7.41 (d, *J* = 7.6 Hz, 2 H).

^13^C NMR (150 MHz, CDCl_3_) δ 40.1, 45.0, 57.3, 98.4, 112.2, 120.6, 133.7, 151.5.

FT-IR (film, cm^−1^): 3367, 2922, 2855, 2777, 2212, 1721, 1669, 1608, 1526, 1469, 1338, 1173, 1132, 1041, 825, 732.

GC/MS (70 eV) *m/z* (%): 189 (M^+^, 6), 102 (6), 58 (100), 42 (6).

HRMS (ESI) *m/z* calcd for [C_11_H_15_N_3_ + H]^+^: 190.1339; found: 190.1341.

#### Ethyl 4-{[2-(dimethylamino)ethyl]amino}benzoate (3ag)

Yellow oil, yield 90%.

^1^H NMR (600 MHz, CDCl_3_) δ 1.11 (t, *J* = 7.0 Hz, 3 H), 2.26 (s, 6 H), 2.53–2.57 (m, 2 H), 3.18–3.19 (m, 2 H), 4.33–4.41 (m, 2 H), 6.56–6.58 (m, 2 H), 7.86–7.88 (m, 2 H).

^13^C NMR (150 MHz, CDCl_3_) δ 14.5, 40.3, 45.0, 57.6, 60.1, 111.5, 118.4, 131.5, 152.0, 167.0

FT-IR (film, cm^−1^): 3395, 2922, 2852, 1682, 1607, 1464, 1341, 1276, 1110, 1041, 839, 700.

GC/MS (70 eV) *m/z* (%): 136 (M^+^, 6), 191 (6), 150 (6), 105 (7), 58 (100).

HRMS (ESI) *m/z* calcd for [C_13_H_20_N_2_O_2_ + H]^+^: 237.1598; found: 237.1600.

#### 3-{[2-(Dimethylamino)ethyl]amino}phenol (3ah)

Yellow oil, yield 70%.

^1^H NMR (600 MHz, CDCl_3_) δ 2.26 (s, 6 H), 2.55–2.57 (m, 2 H), 3.14–3.15 (m, 2 H), 6.12 (s, 1 H), 6.16 (d, *J* = 8.0 Hz, 1 H), 6.23 (d, *J* = 8.0 Hz, 1 H), 7.02 (t, *J* = 8.0 Hz, 1 H).

^13^C NMR (150 MHz, CDCl_3_) δ 41.0, 45.1, 57.9, 99.7, 104.4, 105.8, 134.8, 149.9, 156.7.

FT-IR (film, cm^−1^): 3368, 2928, 1605, 1463, 1261, 1047.

GC/MS (70 eV) *m/z* (%): 180 (M^+^, 16), 122 (10), 65 (6), 58 (100), 42 (7).

HRMS (ESI) *m/z* calcd for [C_10_H_16_N_2_O + H]^+^: 181.1335; found: 181.1336.

#### *N*^1^-(Isoquinolin-4-yl)-*N*^2^,*N*^2^-dimethylethane-1,2-diamine (3ai)

Colorless oil, yield 75%.

^1^H NMR (600 MHz, CDCl_3_) δ 2.38 (s, 6 H), 2.80–2.81 (m, 2 H), 3.39–3.40 (m, 2 H), 5.27 (br. s, 1 H), 7.58–7.61 (m, 1 H), 7.66–7.69 (m, 1 H), 7.85–7.86 (m, 2 H), 7.91–7.92 (m, 1 H), 8.70 (s, 1 H).

^13^C NMR (150 MHz, CDCl_3_) δ 46.6, 47.3, 60.3, 121.7, 124.6, 126.5, 126.9, 128.3, 128.9, 129.8, 134.2, 140.3.

FT-IR (film, cm^−1^): 3391, 2918, 1724, 1651, 1583, 1407, 1285, 1121, 1040, 842, 751.

GC/MS (70 eV) *m/z* (%): 215 (M^+^, 15), 157 (8), 128 (6), 101 (5), 58 (100), 42 (6).

HRMS (ESI) *m/z* calcd for [C_13_H_17_N_3_ + H]^+^: 216.1495; found: 216.1500.

#### *N*^1^,*N*^1^-Dimethyl-*N*^2^-(pyridin-3-yl)ethane-1,2-diamine (3aj)

Yellow oil, yield 70%.

^1^H NMR (600 MHz, CDCl_3_) δ 2.27 (s, 6 H), 2.57–2.59 (m, 2 H), 3.14–3.15 (m, 2 H), 4.37 (br. s, 1 H), 6.88–6.90 (m, 1 H), 7.09–7.10 (m, 1 H), 7.97–7.98 (m, 1 H), 8.07 (s, 1 H).

^13^C NMR (150 MHz, CDCl_3_) δ 40.6, 45.1, 57.7, 118.6, 123.7, 136.1, 138.6, 144.5.

FT-IR (film, cm^−1^): 3335, 2924, 2855, 1652, 1592, 1470, 1421, 1302, 1248, 1192, 1133, 1042, 874, 797, 709.

GC/MS (70 eV) *m/z* (%): 165 (M^+^, 8), 107 (5), 78 (6), 58 (100), 51 (5), 42 (8).

HRMS (ESI) *m/z* calcd for [C_9_H_15_N_3_ + H]^+^: 166.1339; found: 166.1339.

#### *N*^1^,*N*^1^-Dimethyl-*N*^2^-(pyridin-2-yl)ethane-1,2-diamine (3ak)

Yellow oil, yield 98%.

^1^H NMR (600 MHz, CDCl_3_) δ 2.27 (s, 6 H), 2.54–2.56 (m, 2 H), 3.34–3.36 (m, 2 H), 5.01 (br. s, 1 H), 6.41 (d, *J* = 7.3 Hz, 1 H), 6.56 (t, *J* = 7.3 Hz, 1 H), 7.40 (t, *J* = 7.3 Hz, 1 H), 8.09–8.10 (m, 1 H).

^13^C NMR (150 MHz, CDCl_3_) δ 39.4, 45.2, 58.0, 107.4, 112.6, 137.2, 148.0, 158.8.

FT-IR (film, cm^−1^): 3325, 2956, 2926, 2855, 1667, 1455, 1152, 769, 734.

GC/MS (70 eV) *m/z* (%): 165 (M^+^, 8), 107 (5), 95 (6), 78 (6), 71 (9), 58 (100), 51 (5), 42 (8).

HRMS (ESI) *m/z* calcd for [C_9_H_15_N_3_ + H]^+^: 166.1339; found: 166.1342.

#### *N*^1^,*N*^1^-Diethyl-*N*^2^-(3-methoxyphenyl)ethane-1,2-diamine (3bc)

Colorless oil, yield 70%.

^1^H NMR (600 MHz, CDCl_3_) δ 1.03 (t, *J* = 7.0 Hz, 6 H), 2.56 (q, *J* = 7.0 Hz, 4 H), 2.69 (t, *J* = 5.8 Hz, 2 H), 3.13 (t, *J* = 5.8 Hz, 2 H), 6.19 (s, 1 H), 6.25–6.27 (m, 3 H), 7.07 (t, *J* = 8.0 Hz, 1 H).

^13^C NMR (150 MHz, CDCl_3_) δ 11.7, 29.7, 46.7, 55.1, 55.4, 102.4, 106.2, 113.8, 123.0, 149.0, 150.1.

FT-IR (film, cm^−1^): 3380, 2962, 2925, 2852, 1732, 1614, 1585, 1504, 1474, 1425, 1381, 1285, 1243, 1229, 1211, 1161, 1034, 989.

GC/MS (70 eV) *m/z* (%): 222 (M^+^, 13), 136 (9), 92 (6), 86 (100), 58 (6).

HRMS (ESI) *m/z* calcd for [C_13_H_22_N_2_O + H]^+^: 223.1805; found: 223.1808.

#### *N*^1^,*N*^1^-Diethyl-*N*^2^-(pyridin-2-yl)ethane-1,2-diamine (3bk)

Colorless oil, yield 97%.

^1^H NMR (600 MHz, CDCl_3_) δ 1.03 (t, *J* = 7.0 Hz, 6 H), 2.56–2.59 (m, 4 H), 2.68–2.70 (m, 2 H), 3.32–3.33 (m, 2 H), 5.15 (br. s, 1 H), 6.41 (d, *J* = 7.5 Hz, 1 H), 6.54 (d, *J* = 7.5 Hz, 1 H), 7.40 (t, *J* = 7.5 Hz, 1 H), 8.10–8.11 (m, 1 H).

^13^C NMR (150 MHz, CDCl_3_) δ 11.6, 39.4, 46.7, 51.6, 107.4, 112.6, 137.2, 148.0, 158.9.

FT-IR (film, cm^−1^): 3322, 2954, 1668, 1454, 1152, 770, 732.

GC/MS (70 eV) *m/z* (%): 193 (M^+^, 11), 135 (6), 95 (6), 86 (100), 51 (5), 42 (8).

HRMS (ESI) *m/z* calcd for [C_11_H_19_N_3_ + H]^+^: 194.1652; found: 194.1653.

#### *N*-Heptylaniline (3ca)

Yellow oil, yield 97%.

^1^H NMR (600 MHz, CDCl_3_) δ 0.89–0.91 (m, 3 H), 1.30–1.41 (m, 6 H), 1.60–1.65 (m, 4 H), 3.11 (t, *J* = 7.0 Hz, 2 H), 3.49 (br. s, 1 H), 6.61 (d, *J* = 7.9 Hz, 2 H), 6.70 (t, *J* = 7.9 Hz, 1 H), 7.18 (t, *J* = 7.9 Hz, 2 H).

^13^C NMR (150 MHz, CDCl_3_) δ 14.1, 22.6, 27.1, 29.1, 29.6, 31.8, 44.0, 112.7, 115.9, 129.2, 148.7.

FT-IR (film, cm^−1^): 3400, 3010, 2850, 1678, 1450, 650, 780.

GC/MS (70 eV) *m/z* (%): 191 (M^+^, 15), 107 (7), 106 (100), 77 (10).

HRMS (ESI) *m/z* calcd for [C_13_H_21_N + H]^+^: 192.1747; found: 192.1749.

#### *N*-Heptylpyridin-2-amine (3ck)

Colorless oil, yield 98%.

^1^H NMR (600 MHz, CDCl_3_) δ 0.89–0.91 (m, 3 H), 1.30–1.42 (m, 6 H), 1.61–1.65 (m, 4 H), 3.25–3.26 (m, 2 H), 4.49 (br. s, 1 H), 6.41–6.42 (m, 1 H), 6.58–6.59 (m, 1 H), 7.42–7.45 (m, 1 H), 8.11–8.12 (m, 1 H).

^13^C NMR (150 MHz, CDCl_3_) δ 14.0, 22.6, 27.0, 29.1, 30.9, 31.8, 42.2, 105.6, 115.8, 137.3, 148.4, 159.0.

FT-IR (film, cm^−1^): 3326, 2926, 1668, 1604, 1515, 1455, 1377, 1289, 769.

GC/MS (70 eV) *m/z* (%): 192 (M^+^, 13), 176 (5), 121 (26), 108 (23), 107 (100), 94 (38), 78 (26), 41 (6).

HRMS (ESI) *m/z* calcd for [C_12_H_20_N_2_ + H]^+^: 193.1699; found: 193.1670.

#### *N*-Phenethylaniline (3da)

Colorless oil, yield 85%.

^1^H NMR (600 MHz, CDCl_3_) δ 2.93 (t, *J* = 7.0 Hz, 2 H), 3.41 (t, *J* = 7.0 Hz, 2 H), 3.68 (br. s, 1 H), 6.61–6.64 (m, 2 H), 6.70–6.73 (m, 1 H), 7.16–7.21 (m, 2 H), 7.23–7.25 (m, 3 H), 7.31–7.34 (m, 2 H).

^13^C NMR (150 MHz, CDCl_3_) δ 35.5, 45.0, 113.0, 117.5, 126.4, 128.6, 128.8, 129.3, 139.3, 148.0.

FT-IR (film, cm^−1^): 3408, 3025, 2927, 2860, 1601, 1505, 1475, 1319, 1179, 1079, 1029, 869, 747.

GC/MS (70 eV) *m/z* (%): 197 (M^+^, 12), 107 (8), 106 (100), 91 (8), 77 (18), 65 (5), 51 (5).

HRMS (ESI) *m/z* calcd for [C_14_H_15_N + H]^+^: 198.1277; found: 198.1278.

#### *N*-Cyclohexyl-4-methoxyaniline (3eb)

White solid, m.p. 41–42°C, yield 70%.

^1^H NMR (600 MHz, CDCl_3_) δ 1.01–1.37 (m, 4 H), 1.60–2.06 (m, 6 H), 3.47–3.50 (m, 1 H), 3.75 (s, 3 H), 6.57 (d, *J* = 8.8, 2 H), 6.76 (d, *J* = 8.8, 2 H).

^13^C NMR (150 MHz, CDCl_3_) δ 25.1, 26.0, 33.7, 52.8, 55.9, 114.9_1_, 114.9_3_, 141.6, 151.8.

FT-IR (KBr, cm^−1^): 3344, 3039, 3012, 2929, 2851, 1845, 1617, 1588, 1515, 1468, 1445, 1410, 1365, 1291, 1244, 1227, 1184, 1145, 1117, 1092, 1033, 977, 886, 819, 753, 650, 624, 557, 511, 466.

GC/MS (70 eV) *m/z* (%): 205 (M^+^, 66), 163 (12), 162 (100), 149 (17), 134 (13), 108 (22), 77 (5), 55 (5), 41 (6).

HRMS (ESI) *m/z* calcd for [C_13_H_19_NO + H]^+^: 206.1539; found: 206.1540.

#### *N*-Cyclohexylpyridin-2-amine (3ek)

White solid, m.p. 106–107°C, yield 53%.

^1^H NMR (600 MHz, CDCl_3_) δ 1.32–1.40 (m, 3 H), 1.54–1.63 (m, 3 H), 1.85–1.88 (m, 3 H), 1.99–2.00 (m, 2 H), 3.50–3.51 (m, 1 H), 6.69–6.75 (m, 1 H), 6.78–6.81 (m, 1 H), 7.75–7.79 (m, 2 H).

^13^C NMR (150 MHz, CDCl_3_) δ 24.3, 25.1, 32.1, 51.7, 108.0, 111.0, 136.3, 143.7, 153.0.

FT-IR (KBr, cm^−1^): 3265, 3143, 3091, 3069, 3020, 2924, 2852, 1610, 1573, 1520, 1486, 1452, 1416, 1362, 1345, 1285, 1251, 1227, 1152, 1116, 1095, 987, 974, 891, 861, 766, 731, 682, 580, 524.

GC/MS (70 eV) *m/z* (%): 176 (M^+^, 48), 147 (11), 133 (44), 120 (10), 119 (100), 107 (8), 98 (9), 95 (12), 79 (11), 67 (12), 52 (5), 41 (6).

HRMS (ESI) *m/z* calcd for [C_11_H_16_N_2_ + H]^+^: 177.1386; found: 177.1387.

#### 1-[2-(Cyclohexylamino)phenyl]ethan-1-one (3el)

Colorless oil, yield 60%.

^1^H NMR (600 MHz, CDCl_3_) δ 1.28–1.41 (m, 5 H), 1.62–1.64 (m, 1 H), 1.78–1.80 (m, 2 H), 2.01–2.02 (m, 2 H), 2.58 (s, 3 H), 3.42–3.46 (m, 1 H), 6.53 (t, *J* = 8.0 Hz, 1 H), 6.74 (d, *J* = 8.0 Hz, 1 H), 7.32 (t, *J* = 8.0 Hz, 1 H), 7.74 (d, *J* = 8.0 Hz, 1 H).

^13^C NMR (150 MHz, CDCl_3_) δ 24.5, 25.8, 29.6, 32.7, 50.3, 112.1, 113.3, 117.2, 132.9, 134.8, 150.2, 200.6.

FT-IR (film, cm^−1^): 3290, 2927, 2851, 1607, 1574, 1519, 1459, 1421, 1333, 1258, 1230, 1161, 1098, 1036, 952, 888, 802, 744.

GC/MS (70 eV) *m/z* (%): 217 (M^+^, 90), 202 (36), 200 (24), 188 (11), 174 (24), 162 (15), 160 (269), 146 (19), 136 883), 121 (14), 120 (100), 107 (11), 97 (14), 91 (42), 65 (18), 55 (24), 41 (22).

HRMS (ESI) *m/z* calcd for [C_14_H_19_NO + H]^+^: 218.1539; found: 218.1540.

#### *N,N*-Dibutylpyridin-2-amine (3fk)

Colorless liquid, yield 80%.

^1^H NMR (600 MHz, CDCl_3_) δ 0.95 (t, *J* = 7.3 Hz, 6 H), 1.33–1.38 (m, 4 H), 1.56–1.60 (m, 4 H), 3.42 (t, *J* = 7.6 Hz, 4 H), 6.41–6.46 (m, 2 H), 7.36–7.39 (m, 1 H), 8.11–8.12 (m, 1 H).

^13^C NMR (150 MHz, CDCl_3_) δ 14.2, 20.3, 29.9, 48.8, 105.6, 110.5, 136.9, 148.1, 157.7.

FT-IR (film, cm^−1^): 3370, 2923, 2852, 1738, 1596, 1463, 1376.

GC/MS (70 eV) *m/z* (%): 206 (30), 177 (9), 163 (51), 149 (18), 121 (100), 119 (10), 107 (81),78 (31), 41 (10).

HRMS (ESI) *m/z* calcd for [C_13_H_22_N_2_ + H]^+^: 207.1856; found: 207.1857.

#### 4-Phenylmorpholine (3ga)

Colorless solid, m.p. 51–52°C, yield 90%.

^1^H NMR (600 MHz, CDCl_3_) δ 3.17–3.18 (m, 4 H) 3.87–3.89 (m, 4 H), 6.89–6.94 (m, 2 H), 7.25–7.32 (m, 3 H).

^13^C NMR (150 MHz, CDCl_3_) δ 49.2, 67.8, 115.8, 120.1, 129.4, 151.6.

FT-IR (KBr, cm^−1^) 3057, 3023, 3002, 2962, 2888, 2855, 2825, 2761, 2687, 1973, 1598, 1494, 1447, 1376, 1298, 1260, 1228, 1176, 1119, 1064, 1049, 1031, 990, 924, 858, 772, 698, 636, 519.

GC/MS (70 eV) *m/z* (%): 163 (M^+^, 53), 132 (6), 105 (100), 104 (43), 77 (27), 51 (8).

HRMS (ESI) *m/z* calcd for [C_10_H_13_NO + H]^+^: 164.1070; found: 164.1071.

#### 4-(Pyridin-2-yl)morpholine (3gk)

Colorless oil, yield 90%.

^1^H NMR (600 MHz, CDCl_3_) δ 3.51–3.52 (m, 4 H), 3.83–3.85 (m, 4 H), 6.64–6.68 (m, 2 H), 7.49–7.52 (m, 1 H), 8.21–8.22 (m, 1 H).

^13^C NMR (150 MHz, CDCl_3_) δ 45.6, 66.7, 106.9, 113.7, 137.4, 147.9, 159.6.

FT-IR (film, cm^−1^): 3476, 2962, 2892, 2852, 1722, 1593, 1563, 1481, 1436, 1376, 1333, 1311, 1242, 1160, 1120, 1069, 950, 942, 855, 775, 734, 645, 615, 528, 457.

GC/MS (70 eV) *m/z* (%): 164 (M^+^, 36), 163 (28), 119 (23), 107 (39), 106 (14), 79 (100), 78 (27), 52 (12), 51 (12).

HRMS (ESI) *m/z* calcd for [C_9_H_12_N_2_O + H]^+^: 165.1022; found: 165.1021.

#### Typical Procedure for the Ullmann Cross-Coupling Reaction With Aromatic Amines: Synthesis of Diphenylamine 5aa

CuI (10 mol%, 0.05 mmol, 10 mg), iodobenzene (**1n**, 1 equiv, 0.5 mmol, 102 mg, 57 μL), aniline (**4a**, 1 equiv, 0.5 mmol, 47 mg, 46 μL) and the base (*t*-BuOK, 2 equiv, 1 mmol, 112 mg) were suspended in 1 g DES (Gly/ChCl, 2:1 mol mol^−1^), under air, in a vial with a Teflon screw tap under vigorous stirring at 100°C. The reaction was monitored by GC. Then, the mixture was extracted with CPME (3 × 1 mL) and the organic phase was dried over anhydrous Na_2_SO_4_ and filtered over a celite pad. Evaporation of the solvent under reduced pressure afforded the crude that was purified by flash-chromatography on silica gel (hexane/AcOEt 8:2) to provide the desired product **5aa** in 98% yield (83 mg).

#### Diphenylamine (5aa)

White solid, 98%.

^1^H NMR (600 MHz, CDCl_3_) δ 5.70 (br. s, 1 H), 6.93–6.96 (m, 2 H), 7.08–7.10 (m, 4 H), 7.27–7.29 (m, 4 H).

^13^C NMR (150 MHz, CDCl_3_) δ 117.8, 121.0, 129.3, 143.2.

FT-IR (film, cm^−1^): 3380, 3034, 1583, 1489, 739, 685.

GC/MS (70 eV) *m/z* (%): 169 (M^+^, 100), 168 (64), 167 (36), 141 (5), 115 (6), 77 (8), 65 (6), 51 (9).

HRMS (ESI) *m/z* calcd for [C_12_H_11_ + H]^+^: 170.0964; found: 170.0965.

#### 3-Methoxy-*N*-phenylaniline (5ac)

Yellow oil, yield 70%.

^1^H NMR (600 MHz, CDCl_3_) δ 3.80 (s, 3 H), 5.74 (br. s, 1 H), 6.50–6.53 (m, 1 H), 6.66–6.68 (m, 2 H), 6.95–6.98 (m, 1 H), 7.11–7.12 (m, 2 H), 7.17–7.20 (m, 1 H), 7.27–7.31 (m, 2 H).

^13^C NMR (150 MHz, CDCl_3_) δ 55.2, 103.3, 106.2, 110.2, 118.4, 121.3, 129.4, 130.1, 142.8, 144.6, 160.7.

FT-IR (film, cm^−1^): 3379, 3029, 1638, 1517, 1464, 1296, 1177, 870.

GC/MS (70 eV) *m/z* (%): 199 (M^+^, 100), 183 (9), 168 (16), 167 (13), 156 (7), 155 (8), 128 (12), 51 (7).

HRMS (ESI) *m/z* calcd for [C_13_H_13_NO + H]^+^: 200.1070; found: 200.1071.

#### 4-Methoxy-*N*-phenylaniline (5ao)

Yellow oil, yield 70%.

^1^H NMR (600 MHz, CDCl_3_) δ 3.81 (s, 3 H), 4.69 (br. s, 1 H), 6.86–6.88 (m, 3 H), 6.91–6.93 (m, 2 H), 7.08–7.10 (m, 2 H), 7.21–7.24 (m, 2 H).

^13^C NMR (150 MHz, CDCl_3_) δ 55.6, 114.7, 115.7, 119.6, 122.2, 129.3, 130.9, 135.8, 145.1.

FT-IR (film, cm^−1^): 3408, 2936, 2834, 1590, 1512, 1495, 1464, 1419, 1295, 1231, 1176, 1114, 1025, 908.

GC/MS (70 eV) *m/z* (%): 199 (M^+^, 64), 185 (13), 184 (100), 154 (10), 129 (10), 128 (13), 78 (11), 51 (7).

HRMS (ESI) *m/z* calcd for [C_13_H_13_NO + H]^+^: 200.1070; found: 200.1073.

#### 3-Hydroxydiphenylamine (5ap)

Brown solid, m. p. 70°C, yield 90%.

^1^H NMR (600 MHz, CDCl_3_) δ 5.33 (br. s, 1 H), 5.71 (br. s, 1 H), 6.39–6.40 (m, 1 H), 6.58–6.62 (m, 2 H), 6.94–6.97 (m, 1 H), 7.09–7.12 (m, 3 H), 7.26–7.29 (m, 2 H).

^13^C NMR (150 MHz, CDCl_3_) δ 104.0, 107.7, 110.0, 118.7, 121.5, 129.3, 130.3, 142.6, 145.0, 156.6.

FT-IR (KBr, cm^−1^): 3378, 1599, 1507, 1457, 1412, 1318, 1238, 1102, 822, 744, 693, 511.

GC/MS (70 eV) *m/z* (%): 185 (M^+^, 100), 166 (10), 156 (9), 154 (8), 129 (6), 77 (7).

HRMS (ESI) *m/z* calcd for [C_12_H_11_NO + H]^+^: 186.0913; found: 186.0914.

#### 2-Methyl-5-(phenylamino)benzonitrile (5aq)

Yellow oil, yield 70%.

^1^H NMR (600 MHz, CDCl_3_) δ 2.48 (s, 3 H), 6.98–7.07 (m, 3 H), 7.14–7.19 (m, 2 H), 7.29–7.33 (m, 3 H).

^13^C NMR (150 MHz, CDCl_3_) δ 19.6, 114.6, 118.7, 120.0, 121.3, 121.9, 122.3, 129.6, 131.2, 133.7, 141.9.

FT-IR (film, cm^−1^): 3368, 2918, 1605, 1463, 1261, 1047.

GC/MS (70 eV) *m/z* (%): 208 (M^+^ 100), 207 (56), 192 (17), 152 (5), 103 (5), 77 (10), 51 (6).

HRMS (ESI) *m/z* calcd for [C_14_H_12_N_2_ + H]^+^: 209.1073; found: 209.1074.

#### 4-Ethyl-*N*-phenylaniline (5bn)

Yellow solid, m.p.: 86–87, yield 90%.

^1^H NMR (600 MHz, CDCl_3_) δ 1.22 (t, *J* = 7.1 Hz, 3 H), 2.62 (q, *J* = 7.1 Hz, 2 H), 5.63 (br. s, 1 H), 6.89 (t, *J* = 7.5 Hz, 1 H), 7.03–7.05 (m, 4 H), 7.12 (d, *J* = 7.5 Hz, 2 H), 7.25 (d, *J* = 7.5 Hz, 2 H).

^13^C NMR (150 MHz, CDCl_3_) δ 15.3, 29.7, 117.0, 118.8, 120.3, 128.7, 129.3, 134.5, 141.4, 144.3.

FT-IR (film, cm^−1^): 3393, 3052, 3032, 2962, 2924, 2869, 1596, 1514, 1442, 1400, 1313, 1235, 1173, 1115, 1075, 1046, 1028, 994, 876, 847, 818, 743, 692, 615, 549, 506.

GC/MS (70 eV) *m/z* (%): 197 (M^+^, 43), 183 (15), 182 (100), 180 (7), 167 (12), 90 (5), 77 (8).

HRMS (ESI) *m/z* calcd for [C_14_H_15_N + H]^+^: 198.1277; found: 198.1278.

#### 4-Ethyl-*N*-(4-methoxyphenyl)aniline (5bo)

Colorless oil, yield 97%.

^1^H NMR (600 MHz, CDCl_3_) δ 1.22 (t, *J* = 6.7 Hz, 3 H), 2.58 (q, *J* = 6.7 Hz, 2 H), 3.80 (s, 3 H), 5.40 (br. s, 1 H), 6.85–6.89 (m, 4 H), 7.03–7.07 (m, 4 H).

^13^C NMR (150 MHz, CDCl_3_) δ 15.8, 28.0, 55.6, 114.7, 116.5, 121.2, 128.6, 135.9, 136.6, 142.6, 154.8.

FT-IR (film, cm^−1^): 3390, 2924, 2853, 1613, 1463, 1295, 1243, 1180, 1038, 821.

GC/MS (70 eV) m/z (%): 227 (M^+^, 62), 213 (16), 212 (100), 197 (10), 168 (11), 77 (5).

HRMS (ESI) m/z calcd for [C_15_H_17_NO + H]^+^: 228.1383; found: 228.1384.

#### 3,5-Dimethyl-*N*-phenylaniline (5cn)

Yellow oil, yield 60%.

^1^H NMR (600 MHz, CDCl_3_) δ 2.29 (s, 6 H), 5.62 (br. s, 1 H), 6.61 (s, 1 H), 6.73 (s, 2 H), 6.93 (t, *J* = 7.5 Hz, 1 H), 7.07 (d, *J* = 7.5 Hz, 2 H), 7.28 (t, *J* = 7.5 Hz, 2 H).

^13^C NMR (150 MHz, CDCl_3_) δ 21.4, 115.6, 117.9, 120.8, 122.9, 129.3, 139.0, 143.1, 143.3.

FT-IR (film, cm^−1^): 3383, 3025, 2914, 1583, 1516, 1496, 1327, 1265, 1169, 1081, 1034, 992, 878, 843.

GC/MS (70 eV) *m/z* (%): 197 (M^+^, 100), 181 (21), 180 (16), 167 (11), 164 (8), 121 (18), 120 (10), 106 (11), 91 (8), 77 (14), 65 (6).

HRMS (ESI) *m/z* calcd for [C_14_H_15_N + H]^+^: 198.1277; found: 198.1278.

#### Recycling of Cu Catalyst, DES and Base in the Coupling Reaction Between Bromobenzene (1a) and *N,N-*dimethylethylenediamine (2a) to Prepare 3aa. Typical Procedure

CuI (10 mol%, 0.05 mmol, 10 mg), bromobenzene (**1a**, 1 equiv, 0.5 mmol, 79 mg, 53 μL), *N, N*-dimethylethylenediamine (**2a**, 1 equiv, 0.5 mmol, 44 mg, 55 μL) and the base (K_2_CO_3_, 2 equiv, 1 mmol, 138 mg) were sequentially added in 1.0 g DES (Gly/ChCl, 2:1 mol mol^−1^), under air, in a vial with a Teflon screw tap. The reaction mixture was vigorously stirred at 60°C for 12 h (monitoring the complete consumption of the starting material by GC), and then cooled to room temperature. The product was extracted with CPME (1 mL) leaving the catalyst and the base in the eutectic mixture, which was re-used for further reaction runs. The organic layer was filtered through a celite pad, the volatile was evaporated under vacuum, and the crude so obtained was analyzed by ^1^H NMR to determine the yield of **3aa** (CH_2_Br_2_ was used as the internal standard). New, fresh reagents were then added to the recovered eutectic mixture, and the whole procedure was repeated for five times without any significant loss of the catalyst activity (96% yield after the sixth cycle) (see Figure 2). By analyzing the Cu amount in the CPME extract phase by ICP-OES, we conclude that up to 2% of the total amount of copper in the catalyst was lost during each extraction process.

## Data Availability Statement

All datasets generated for this study are included in the article/Supplementary Material.

## Author Contributions

AQ undertook all the synthetic experimental works. AQ and FP analyzed and collected experimental data. VC directed the project. PV, FP, and VC supervised the synthetic chemistry works. PV and FP carried out the characterization chemistry works. VC analyzed and drafted the manuscript, while AQ, PV, FP, and VC contributed to the discussion, to the writing, and the review.

### Conflict of Interest

The authors declare that the research was conducted in the absence of any commercial or financial relationships that could be construed as a potential conflict of interest.
