# Supplementary material for: Reshaping Ullmann Amine Synthesis in Deep Eutectic Solvents: A Mild Approach for Cu-Catalyzed C–N Coupling Reactions With No Additional Ligands
Source: Front Chem. 2019 Oct 30;7:723. doi: 10.3389/fchem.2019.00723 (PMC6833937; doi:10.3389/fchem.2019.00723)

SUPPLEMENTARY MATERIAL

TABLE OF CONTENTS

1. E Factor calculation p. S2

2. ^{1}H and ^{13}C NMR spectra of compounds **3** and **5**. p. S3

1. E Factor calculation

According to its original definition (*Green Chem*. **2007**, *9*, 1273), the Sheldon E factor value (total mass of waste/mass of product) takes into account only the mass of waste generated in a process, and its calculation is performed by simply dividing the sum of the molecular weight of all substances produced by molecular weight of the desired products, with reference to the stoichiometric equation. Thus, the amount of silica gel, the celite pad, the drying agents, and the mass of eluent solvent used for chromatography are usually not included in the calculation. We have followed this general equation in our own calculation.

Note: CPME 0.86 g/mL, at 25 °C.

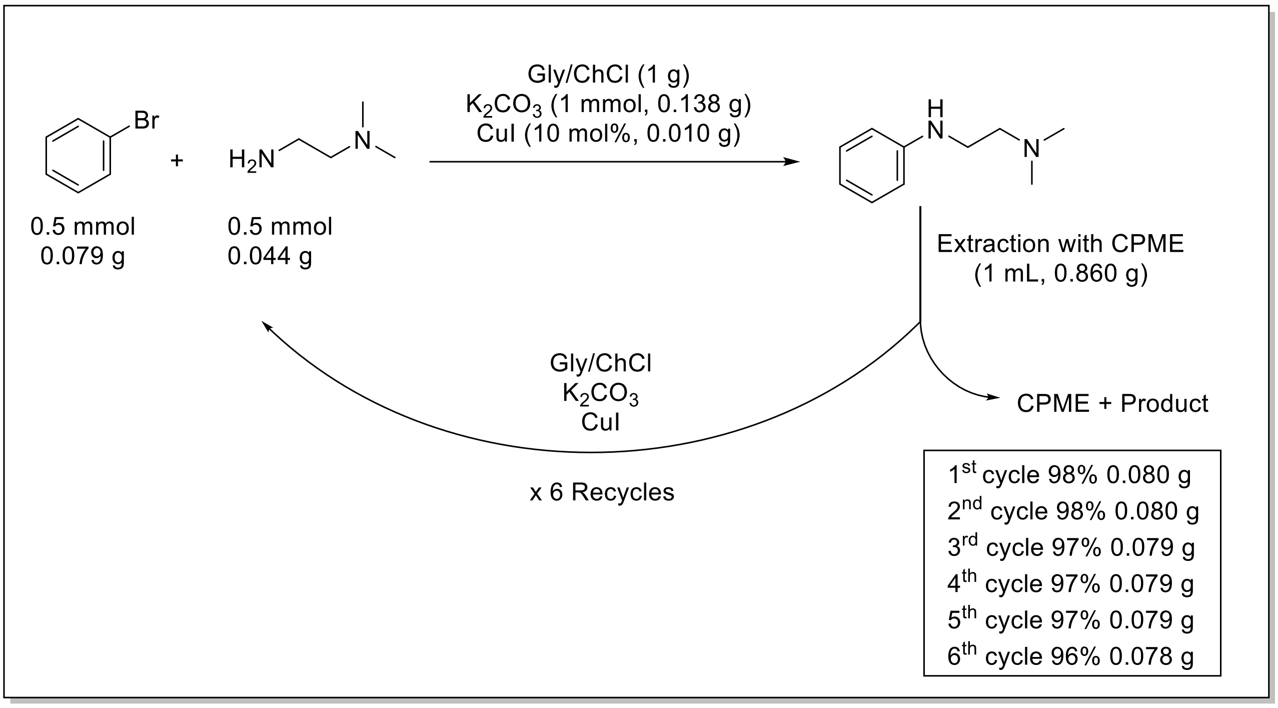

Total amount of reactants: 1 g + 6× 0.079 g + 6× 0.044 g + 0.010 g + 0.138 g + 6× 0.860 g = 7.046 g

Amount of final product: 0.475 g

Amount of waste: 7.046 g – 0.475 g = 6.571 g

E-Factor = amount of waste/amount of product = 6.571 g/0.475 g = **13.8**.

2. ^{1}H and ^{13}C NMR spectra

^{1}H NMR 600MHz, $CDCl_3$

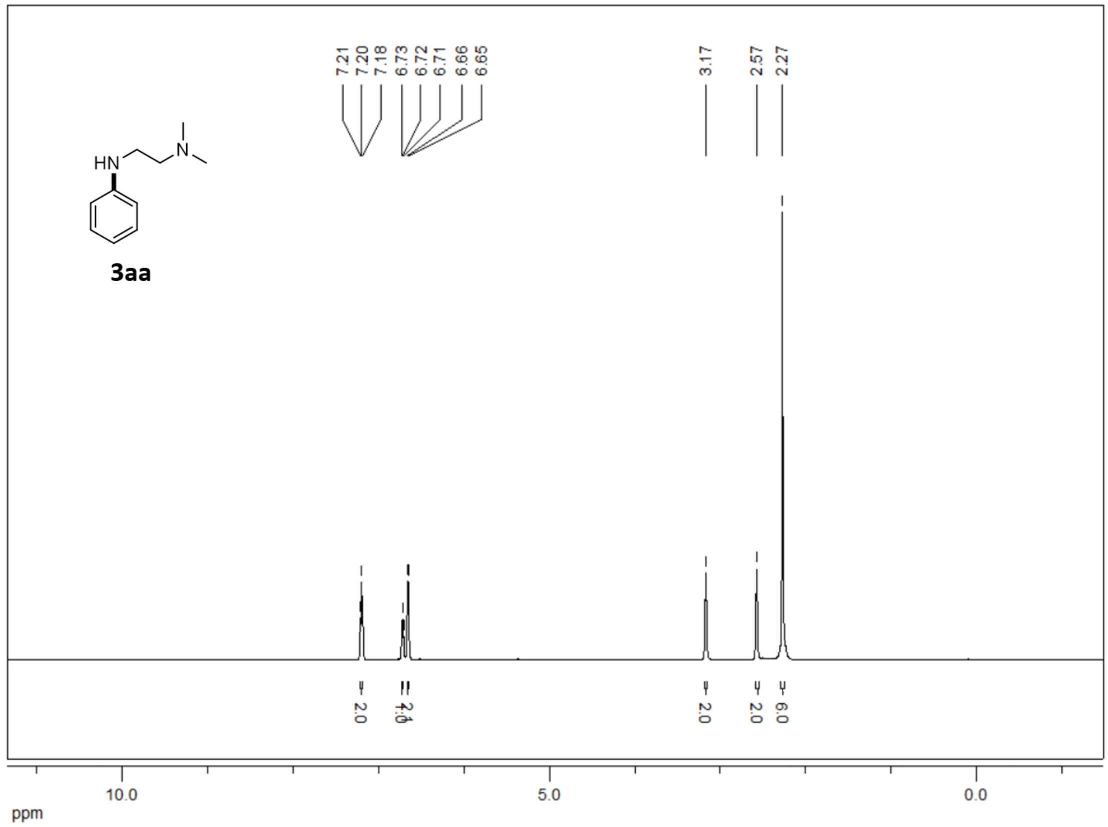

^{13}C NMR 150MHz, $CDCl_3$

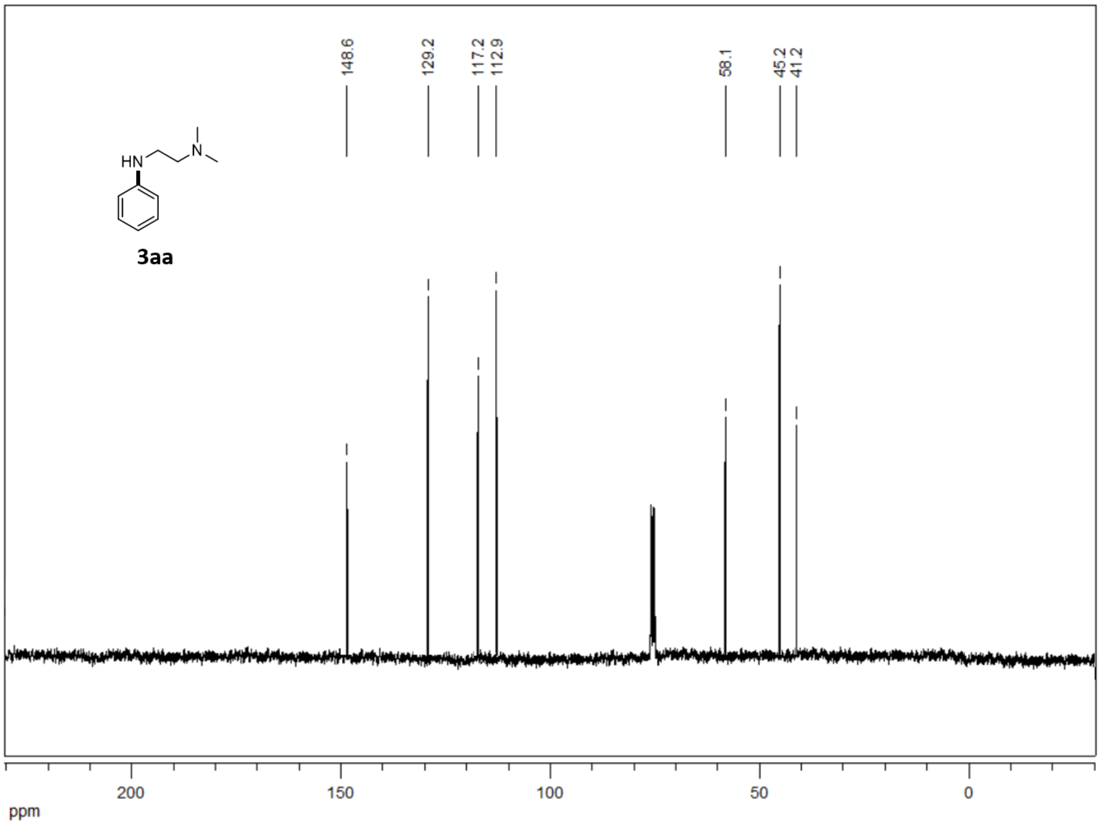

^{1}H NMR 600MHz, $CDCl_3$

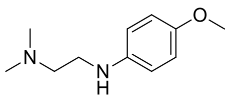

3ab

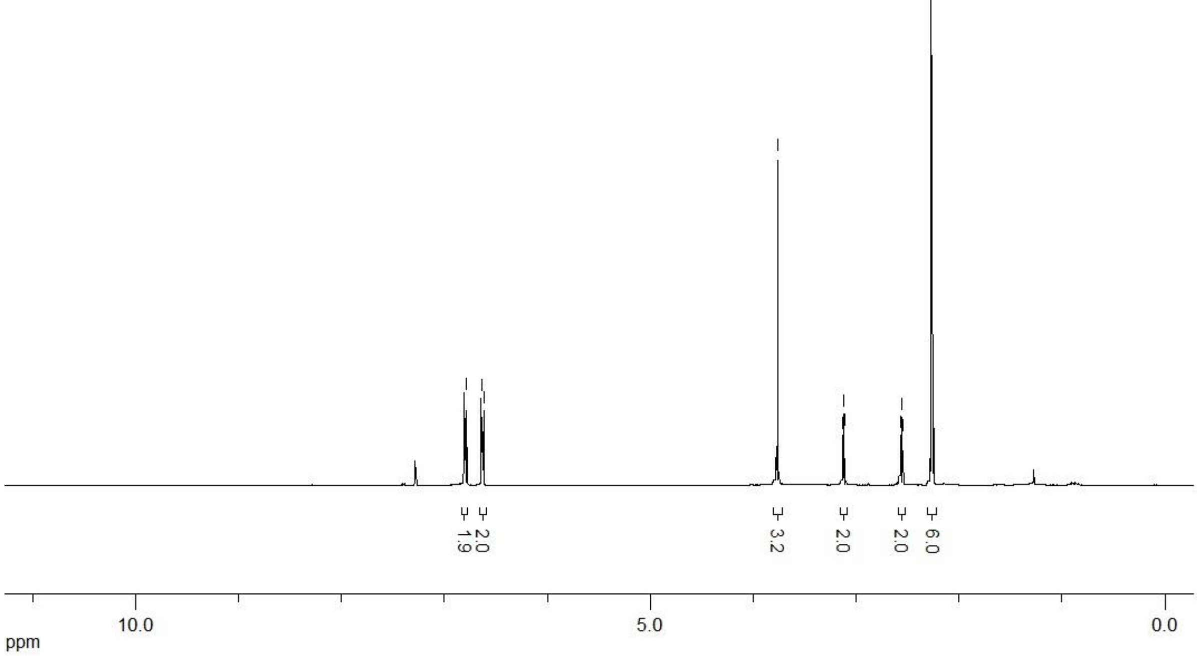

^{13}C NMR 150MHz, $CDCl_3$

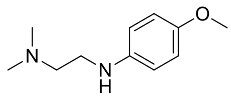

3ab

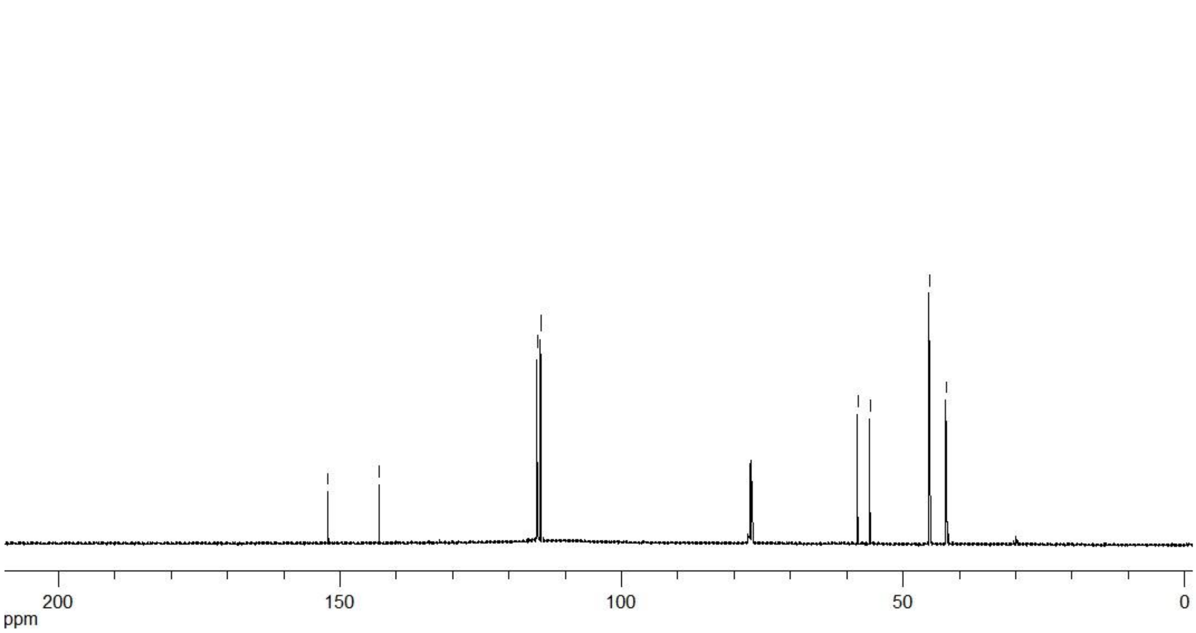

^{1}H NMR 600MHz, $CDCl_3$

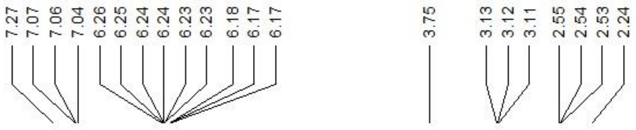

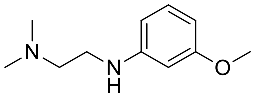

3ac

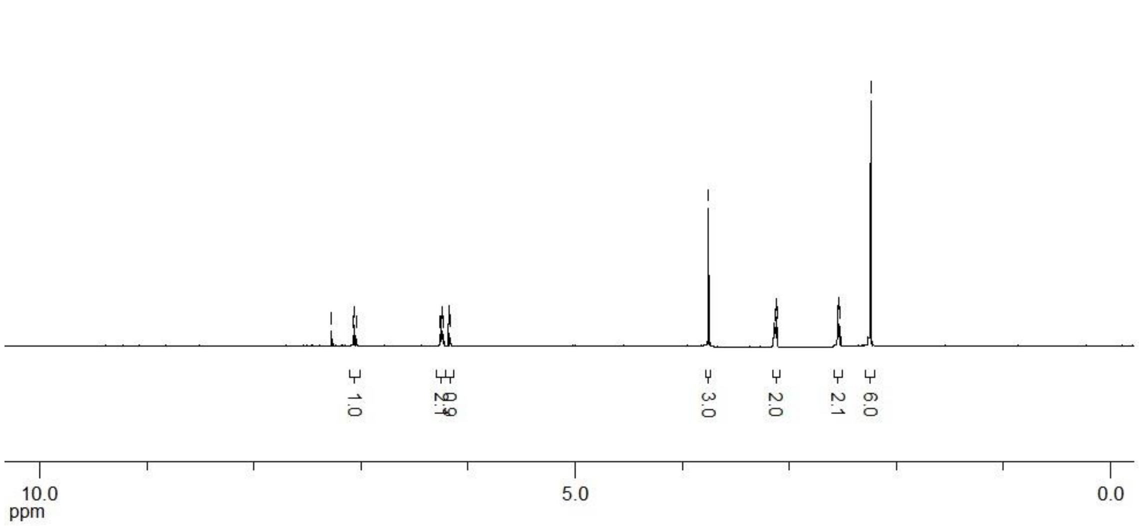

^{13}C NMR 150MHz, $CDCl_3$

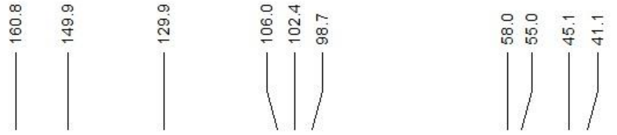

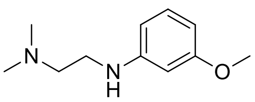

3ac

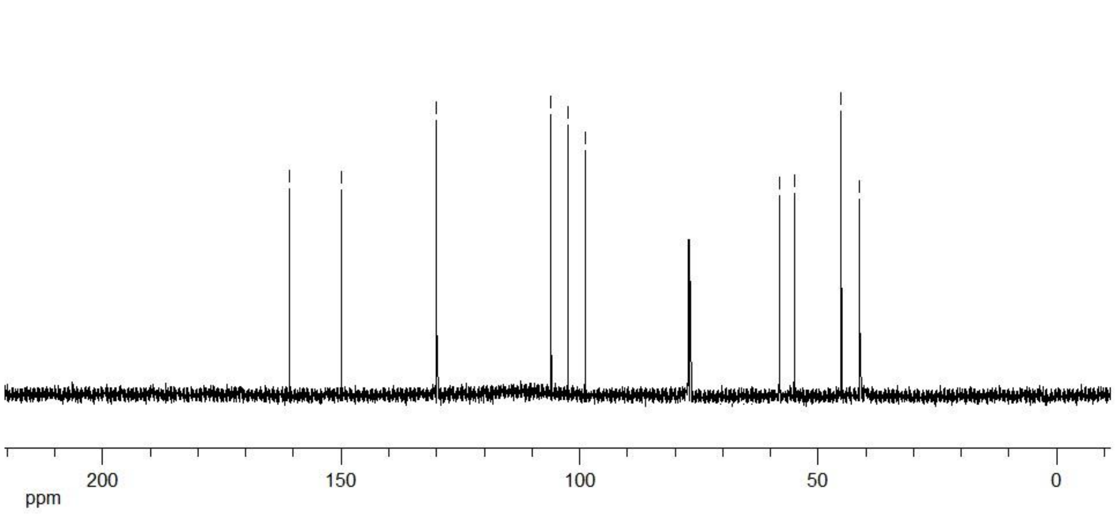

^{1}H NMR 600MHz, $CDCl_3$

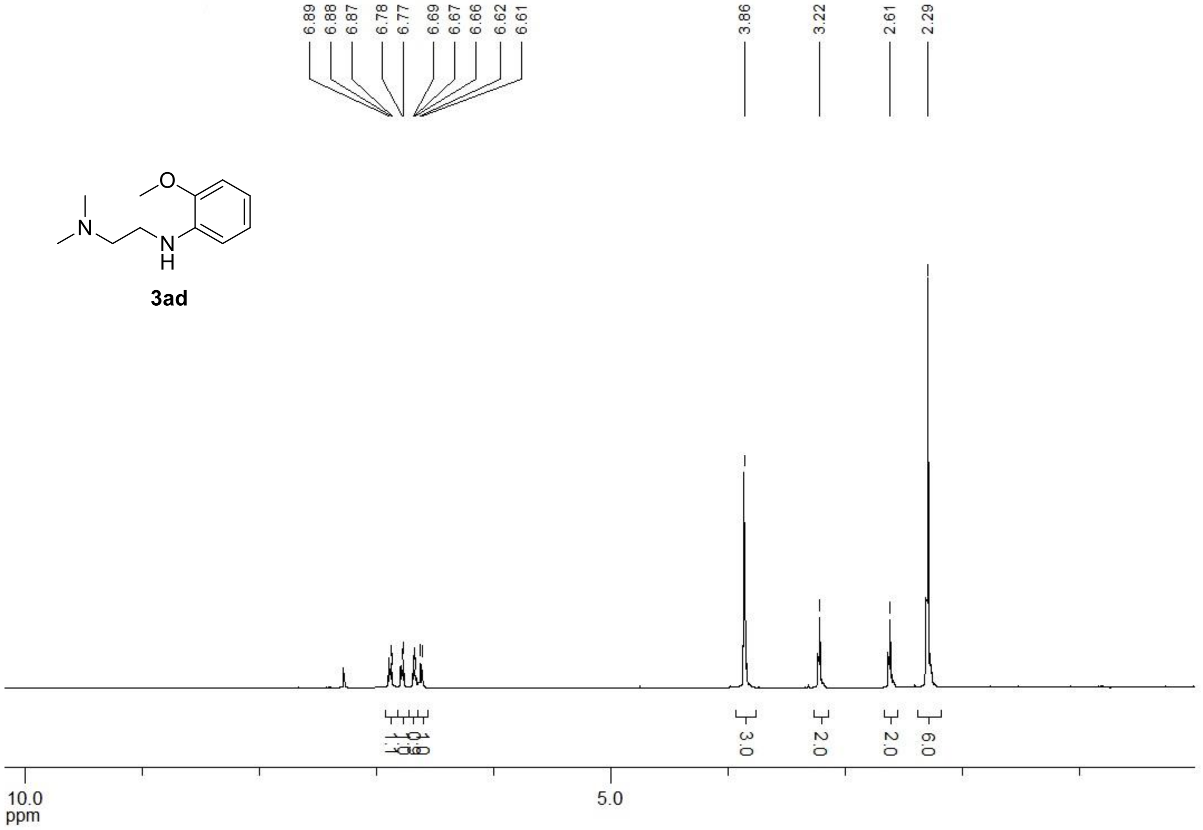

^{13}C NMR 150MHz, $CDCl_3$

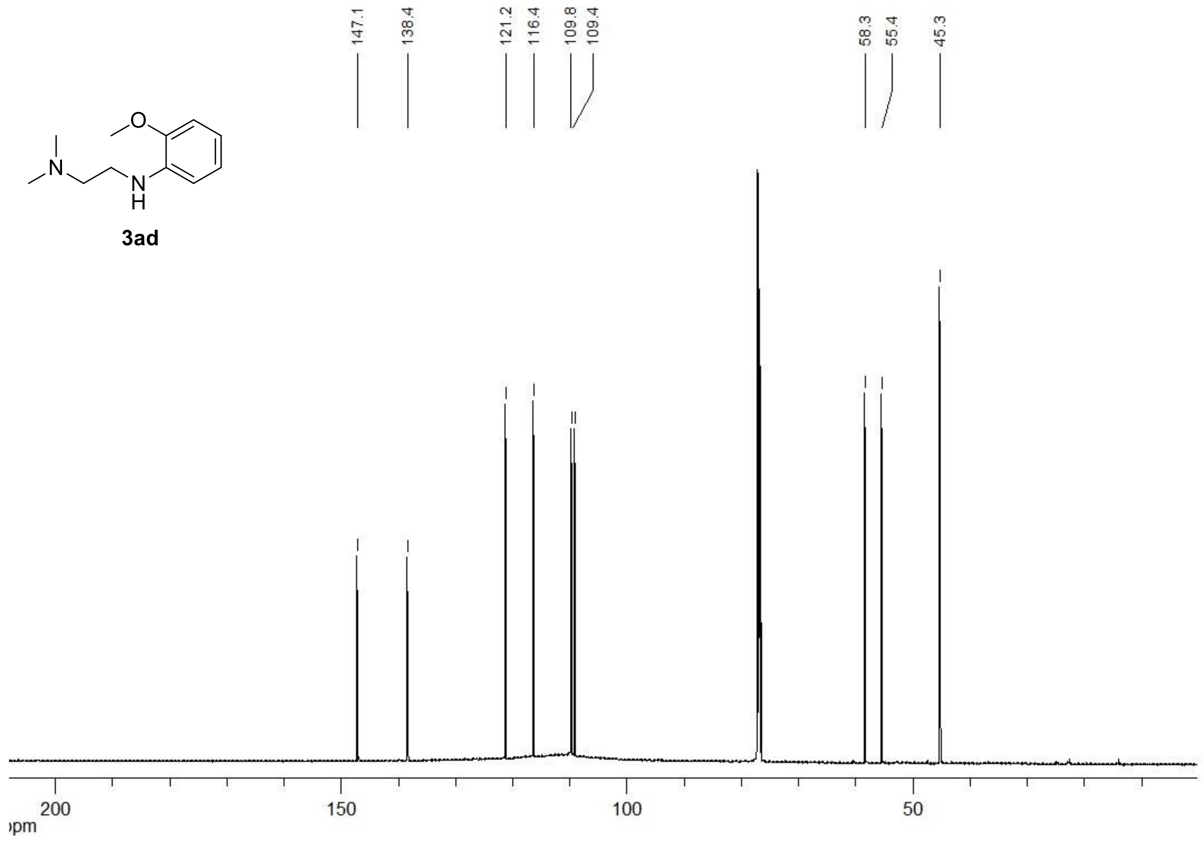

^{1}H NMR 600MHz, $CDCl_3$

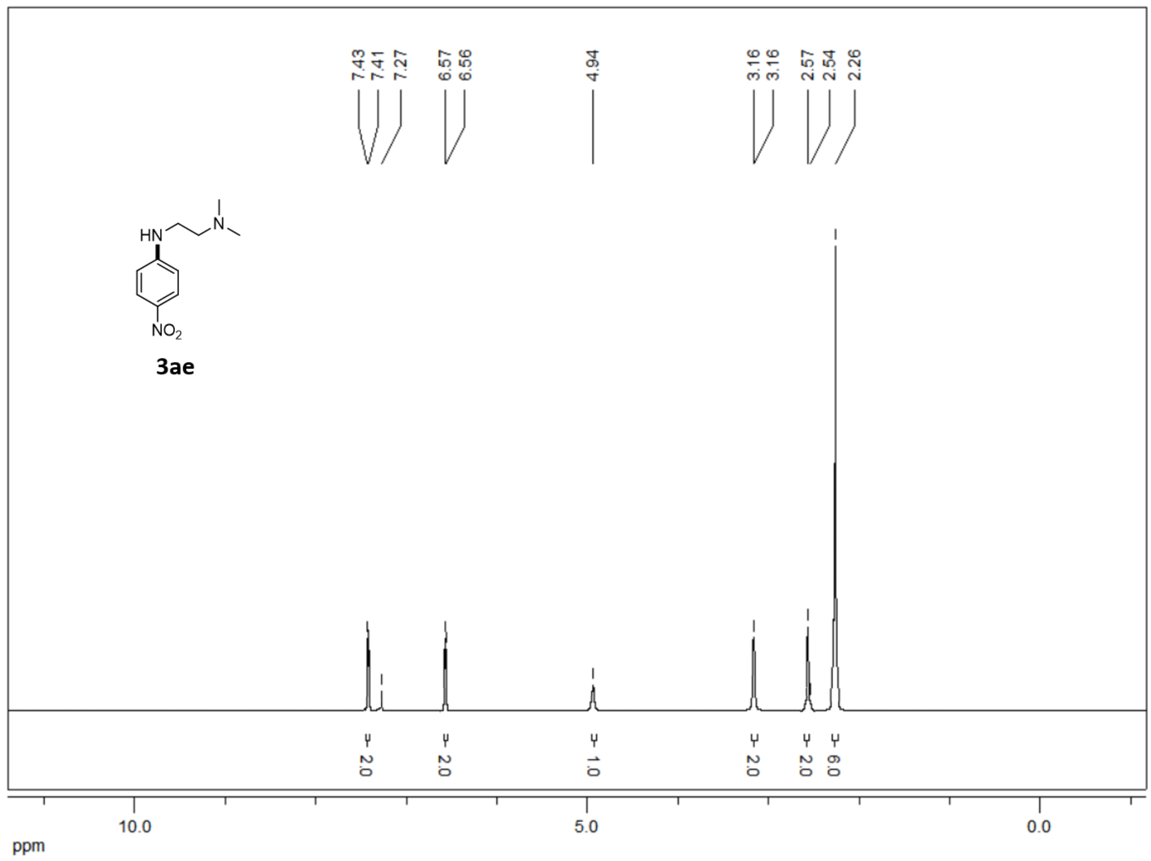

^{13}C NMR 150MHz, $CDCl_3$

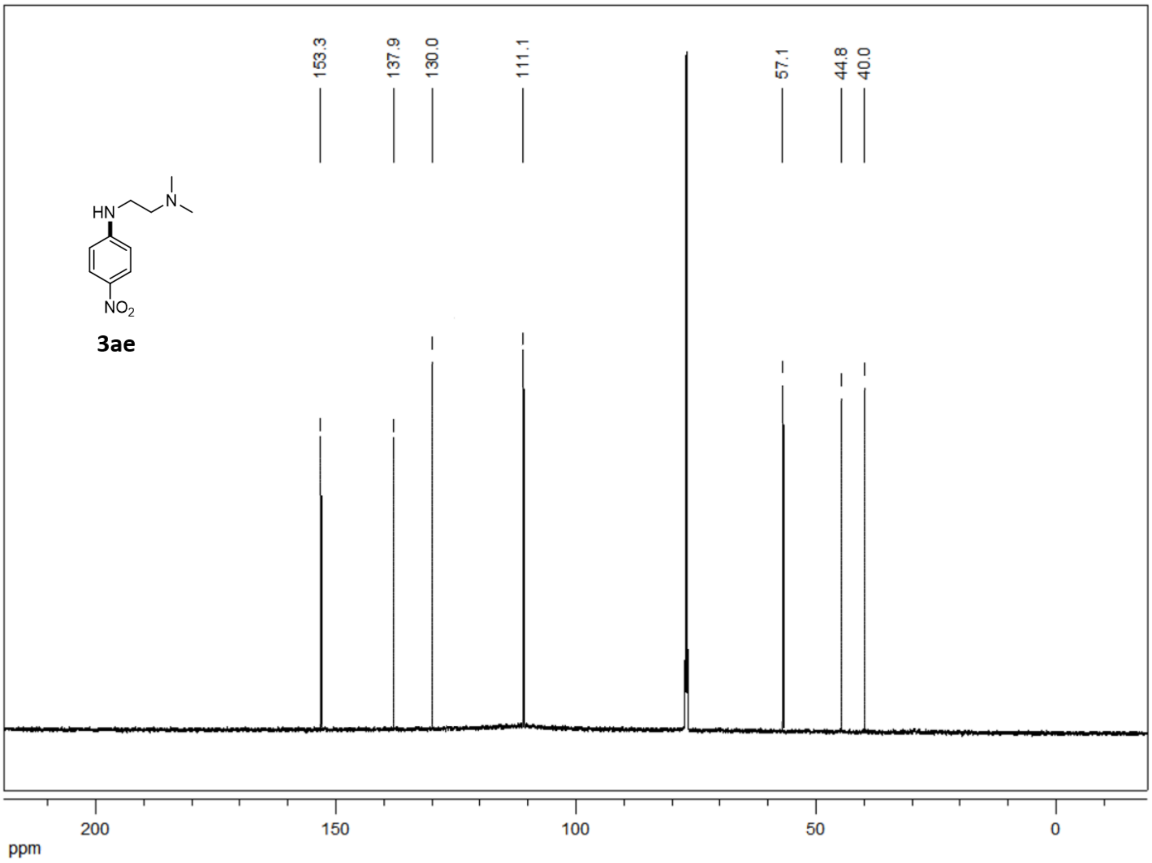

^{1}H NMR 600MHz, $CDCl_3$

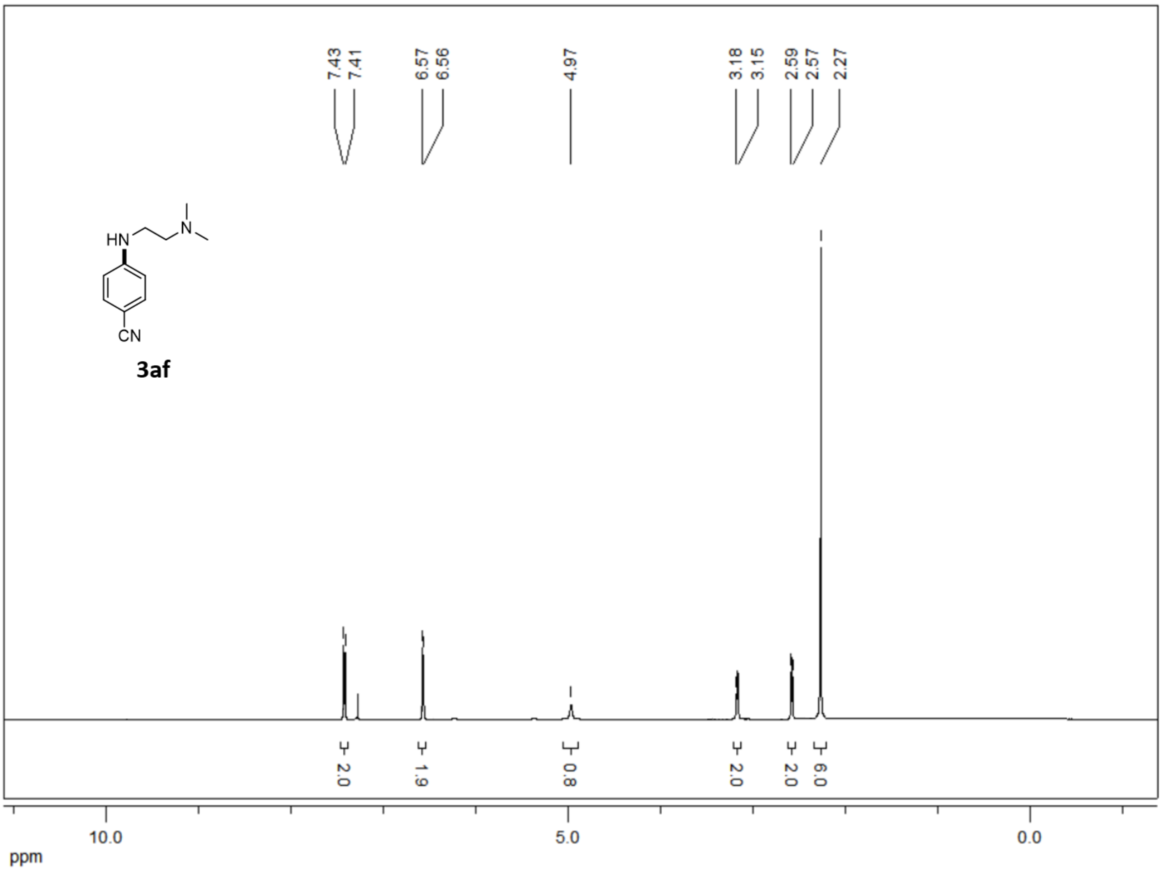

^{13}C NMR 150MHz, $CDCl_3$

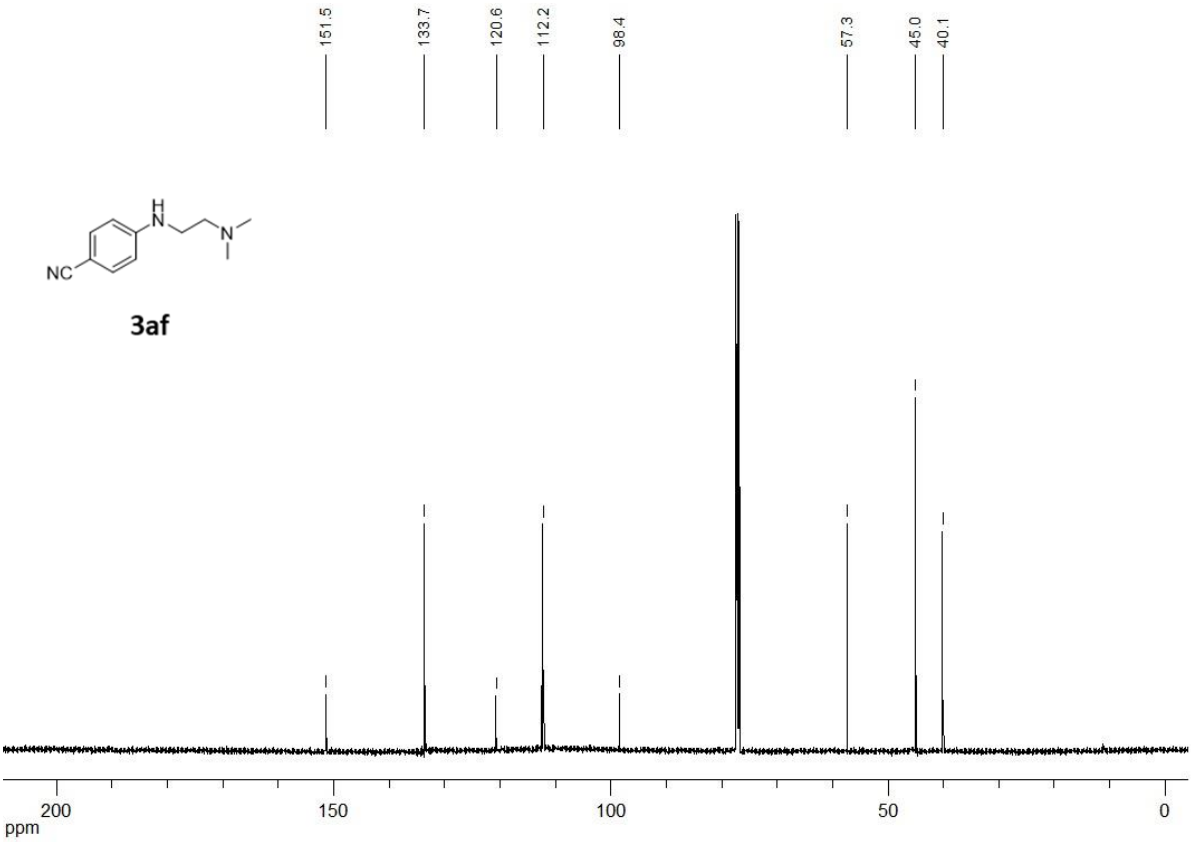

^{1}H NMR 600MHz, $CDCl_3$

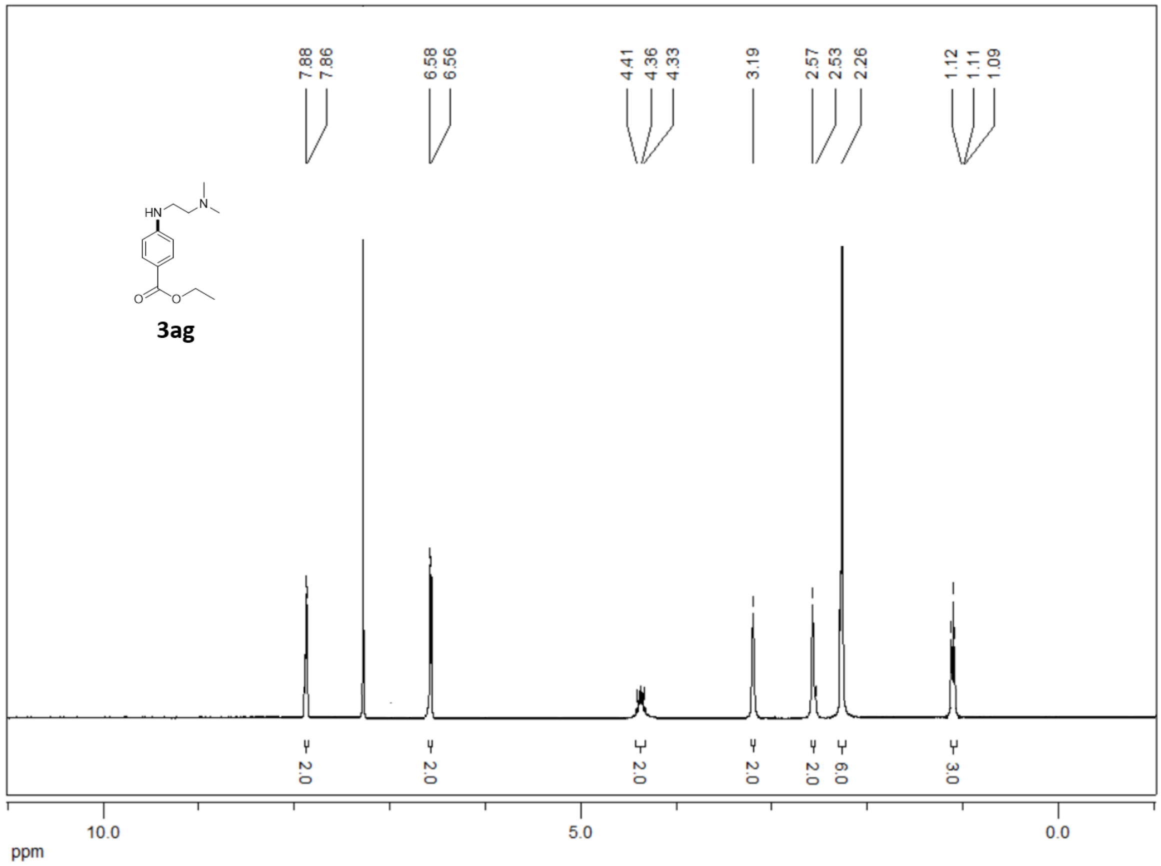

^{13}C NMR 150MHz, $CDCl_3$

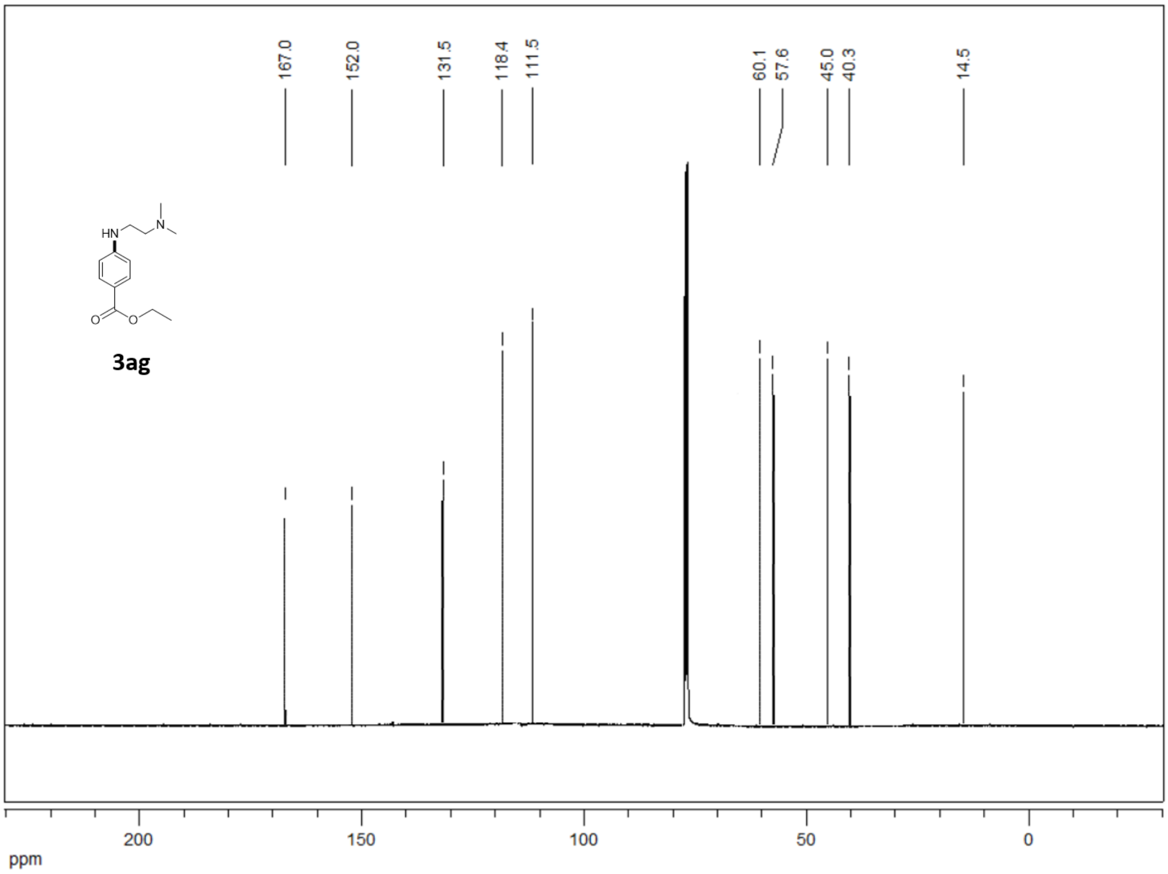

^{1}H NMR 600MHz, $CDCl_3$

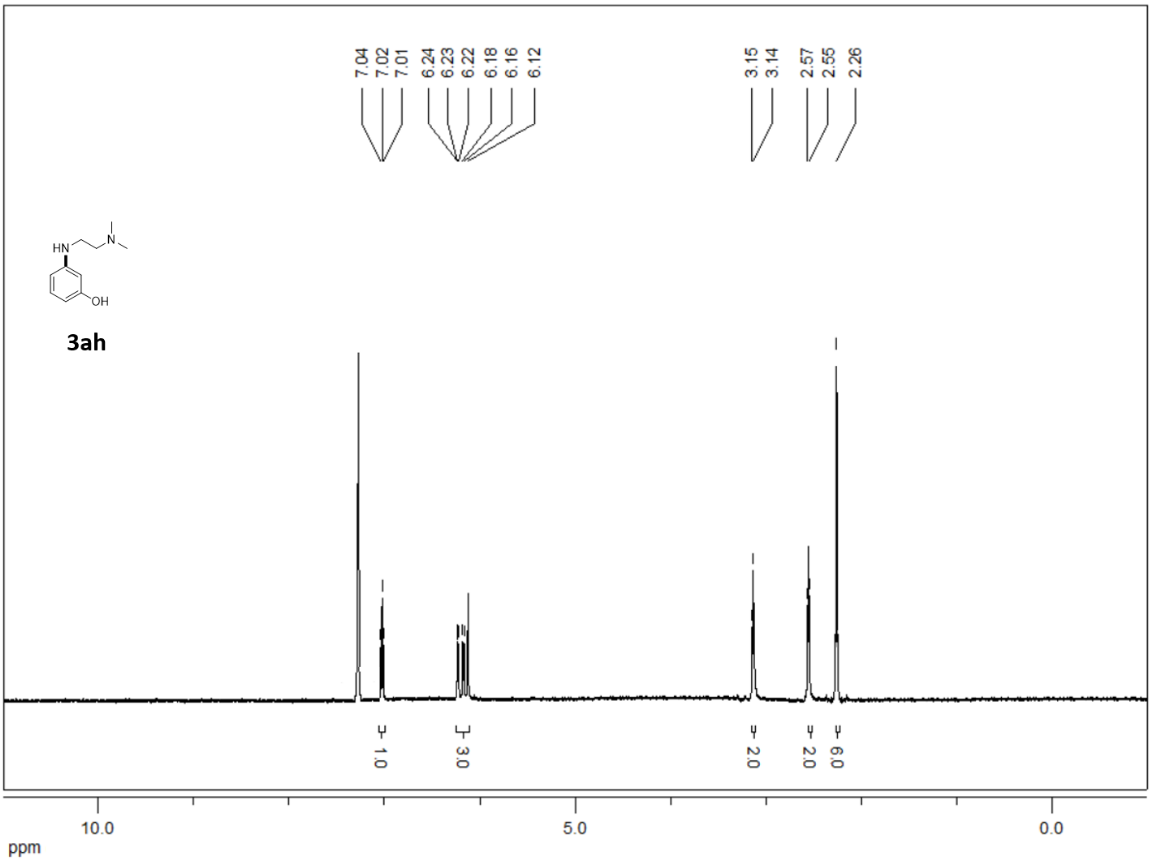

^{13}C NMR 150MHz, $CDCl_3$

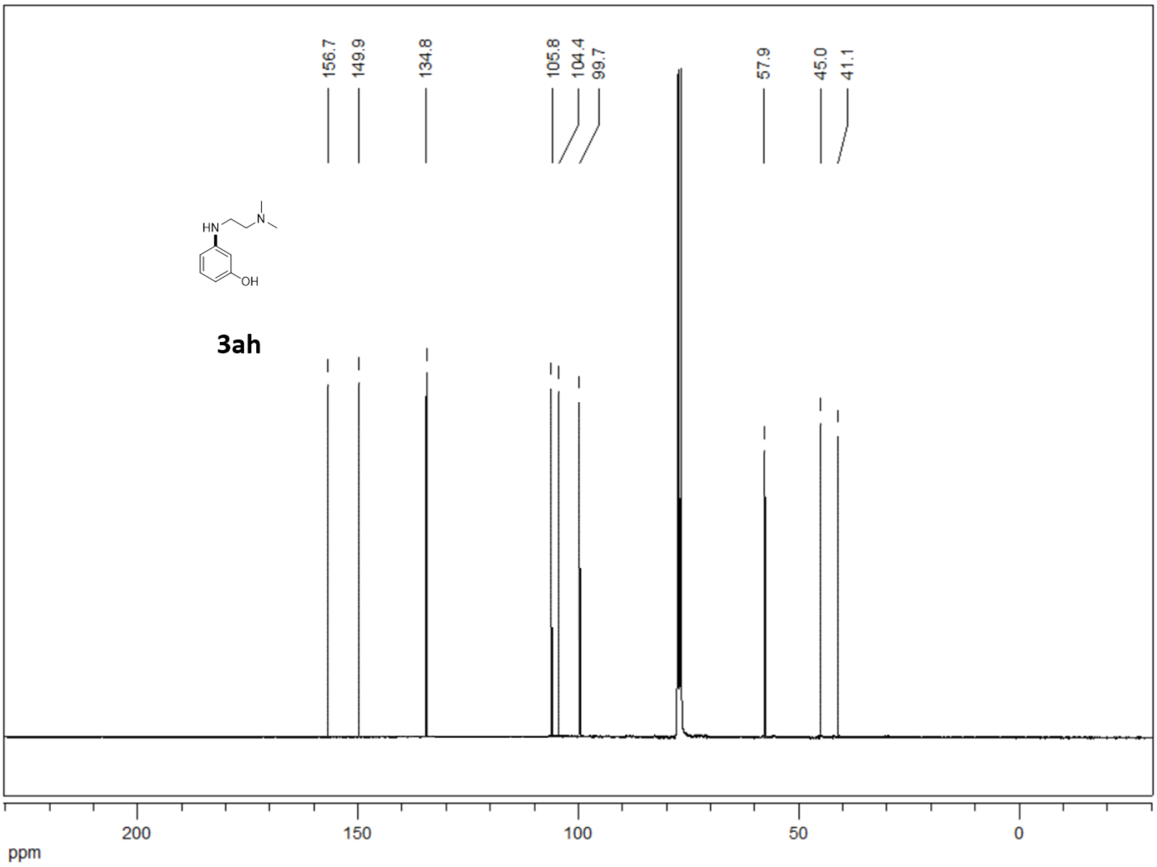

^{1}H NMR 600MHz, $CDCl_3$

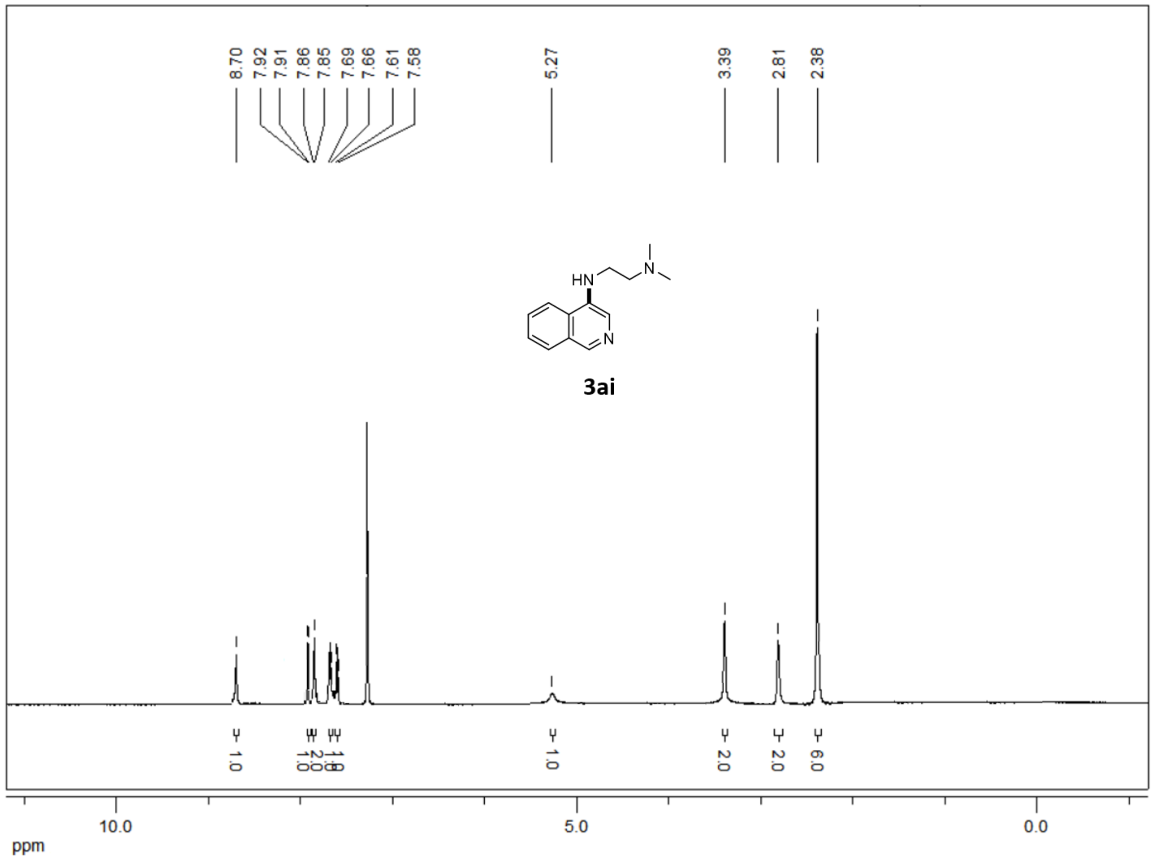

^{13}C NMR 150MHz, $CDCl_3$

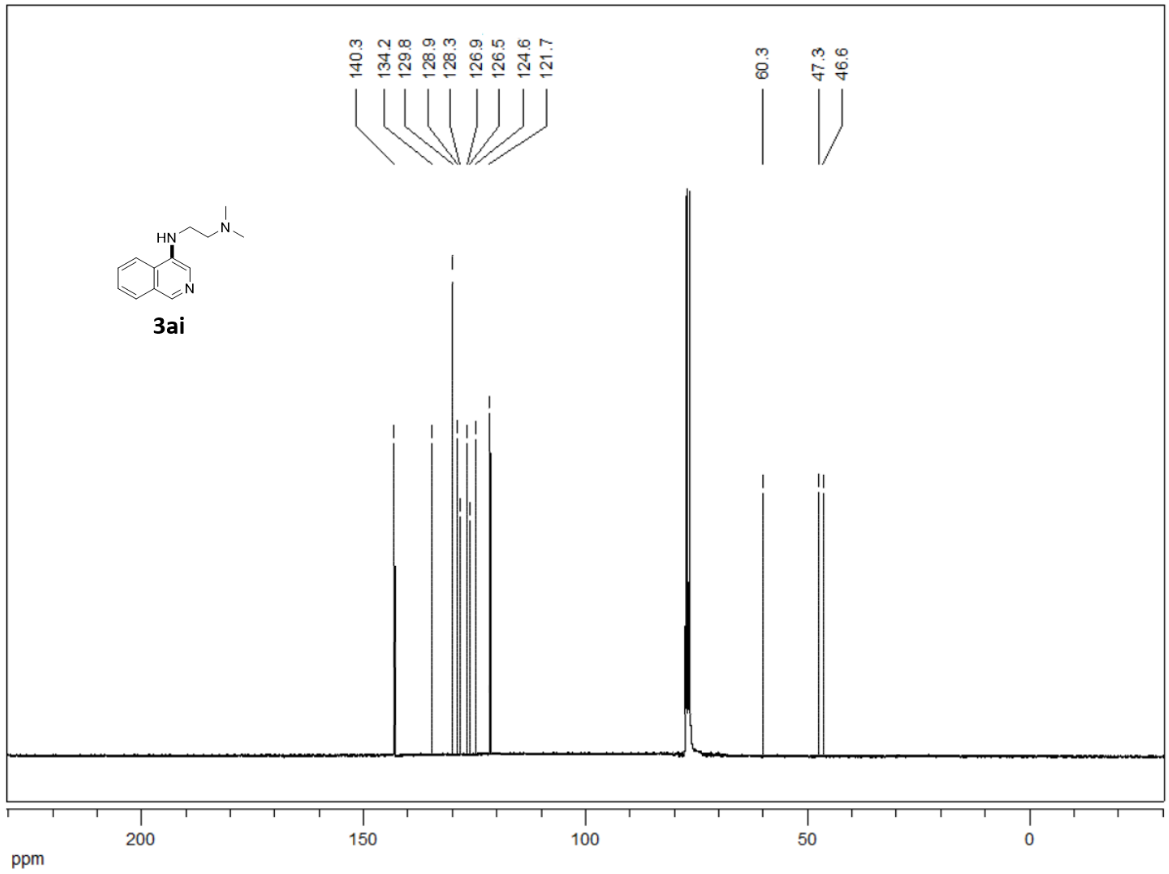

^{1}H NMR 600MHz, $CDCl_3$

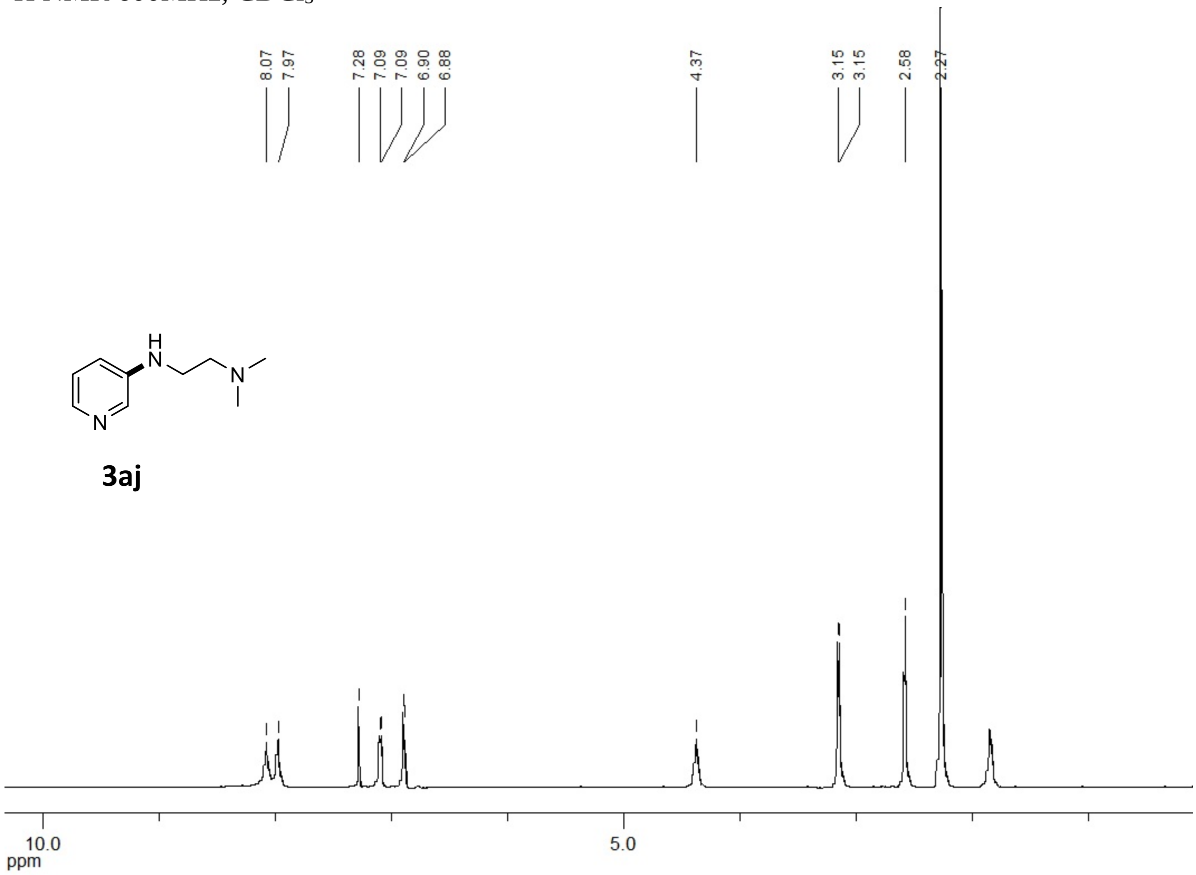

^{13}C NMR 150MHz, $CDCl_3$

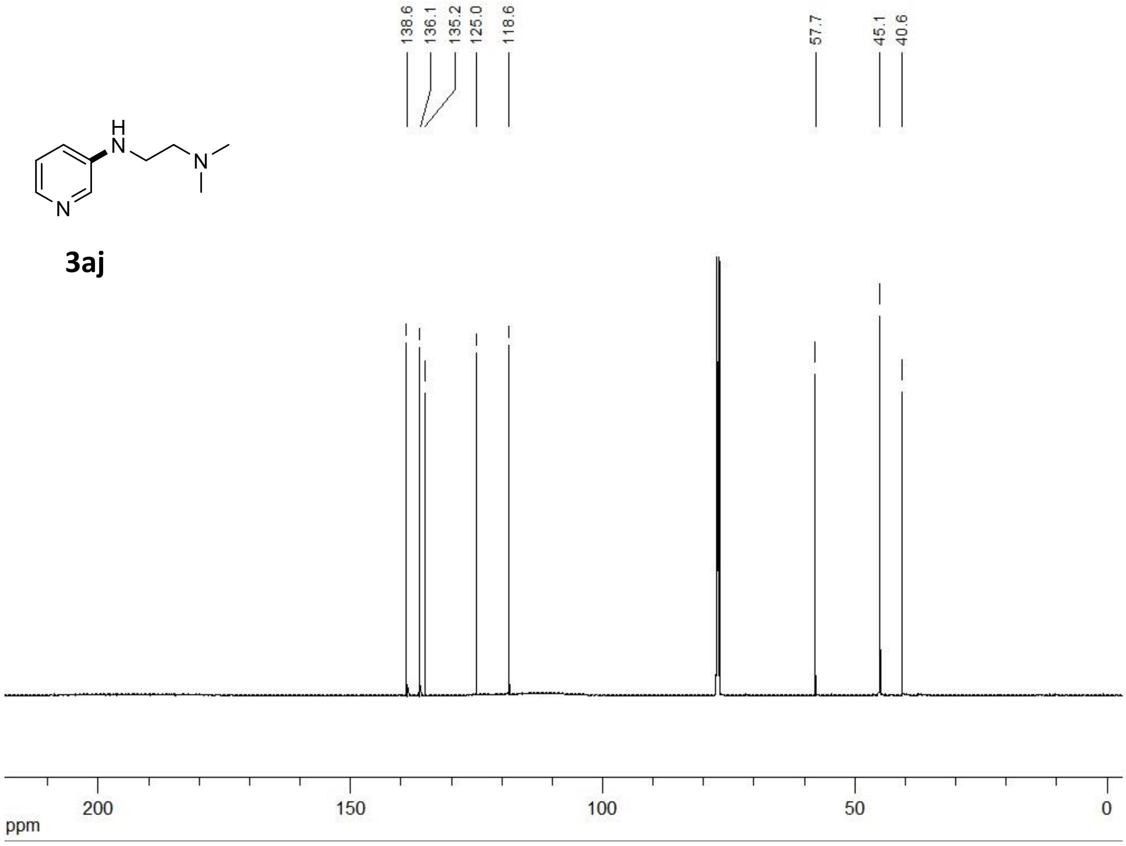

^{1}H NMR 600MHz, $CDCl_3$

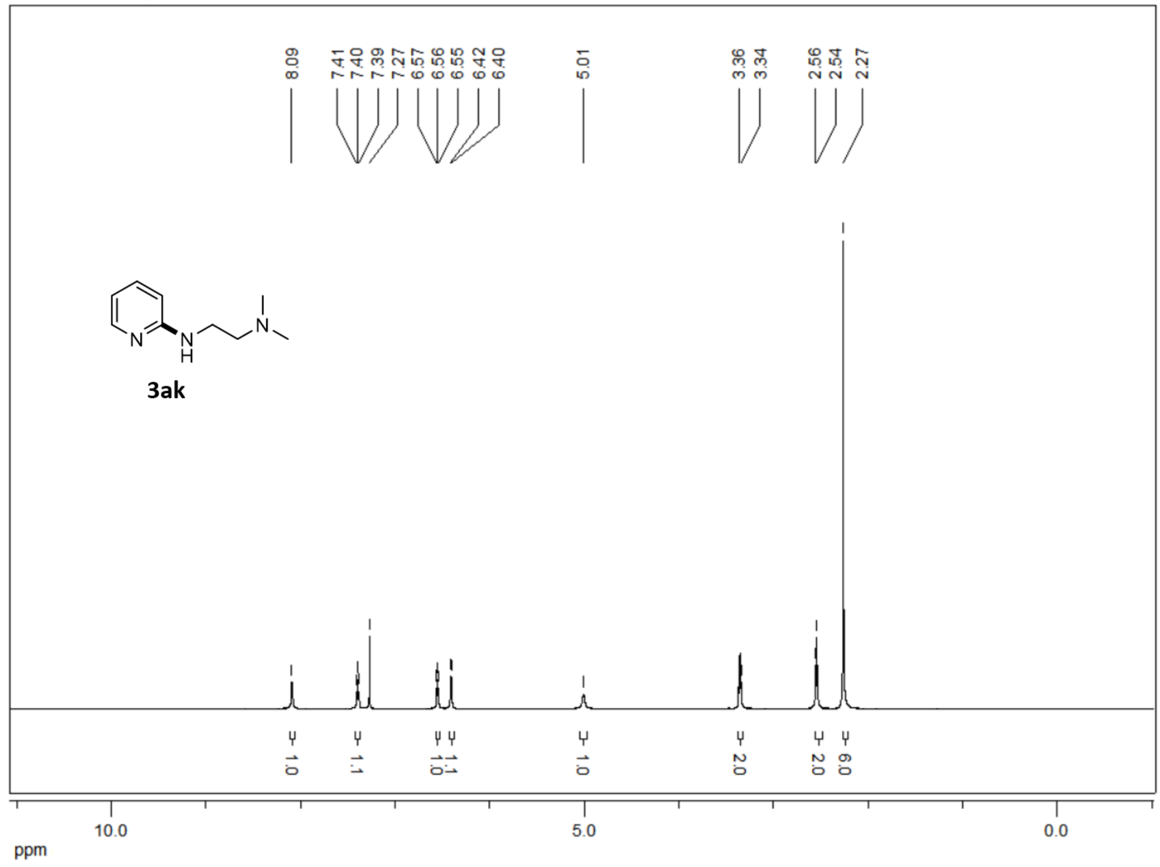

^{13}C NMR 150MHz, $CDCl_3$

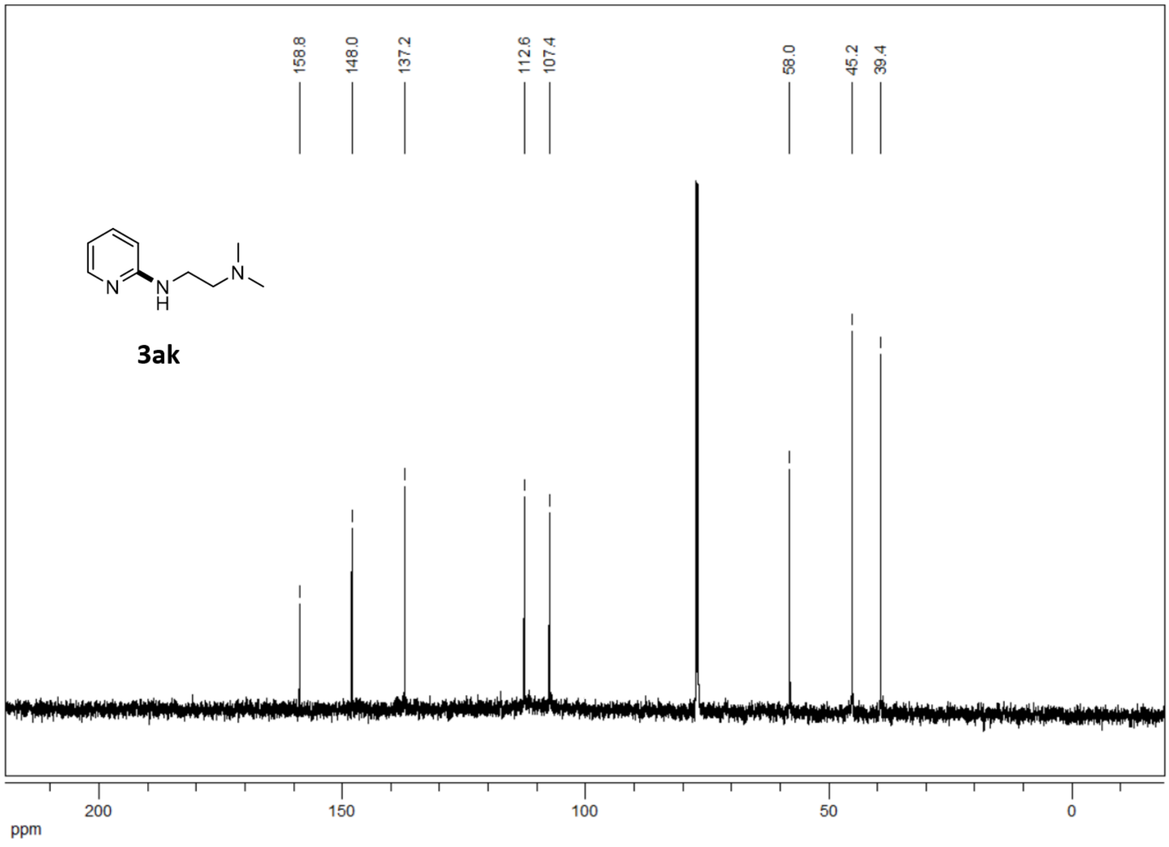

^{1}H NMR 600MHz, $CDCl_3$

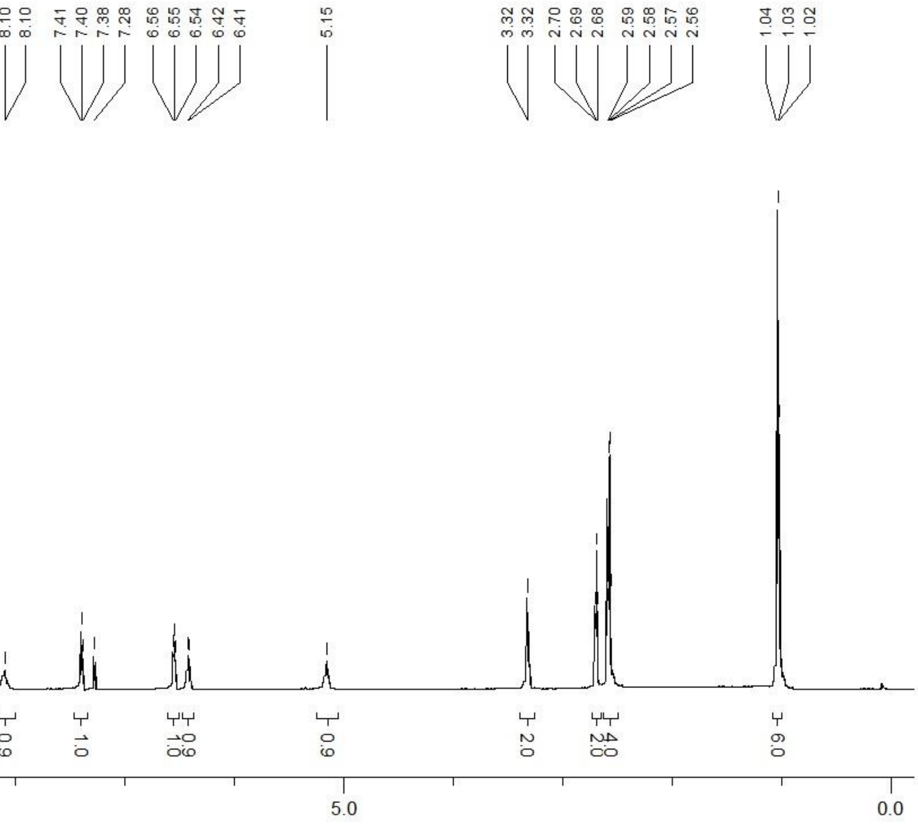

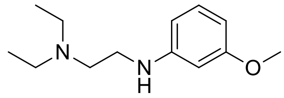

3bc

^{13}C NMR 150MHz, $CDCl_3$

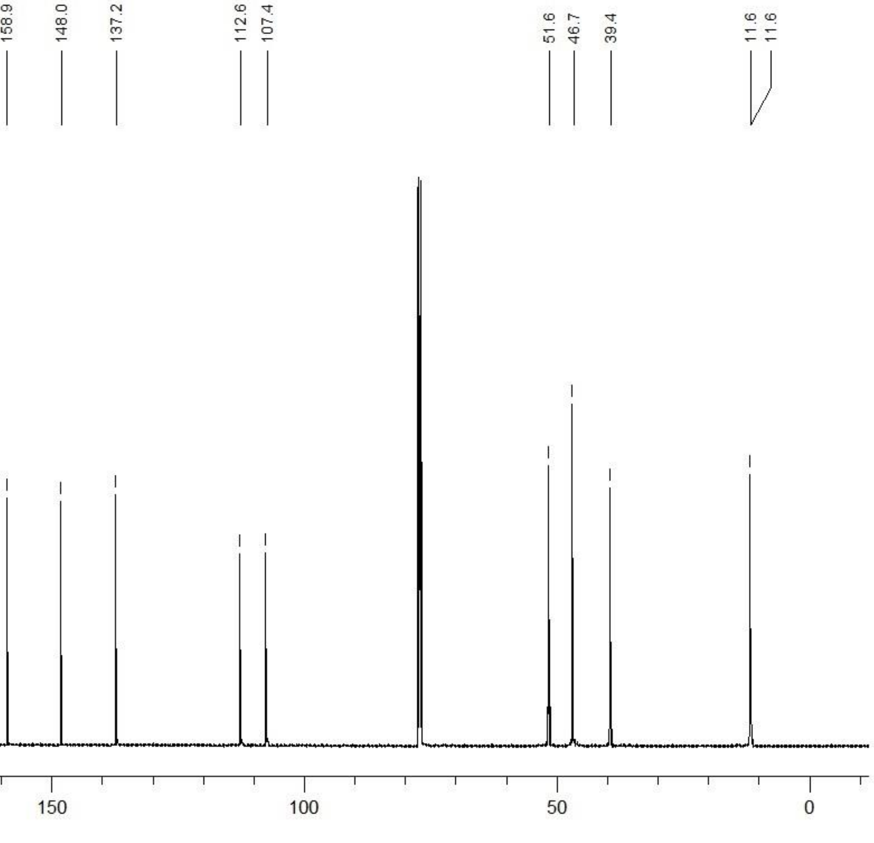

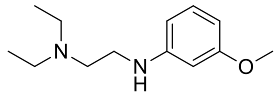

3bc

^{1}H NMR 600MHz, $CDCl_3$

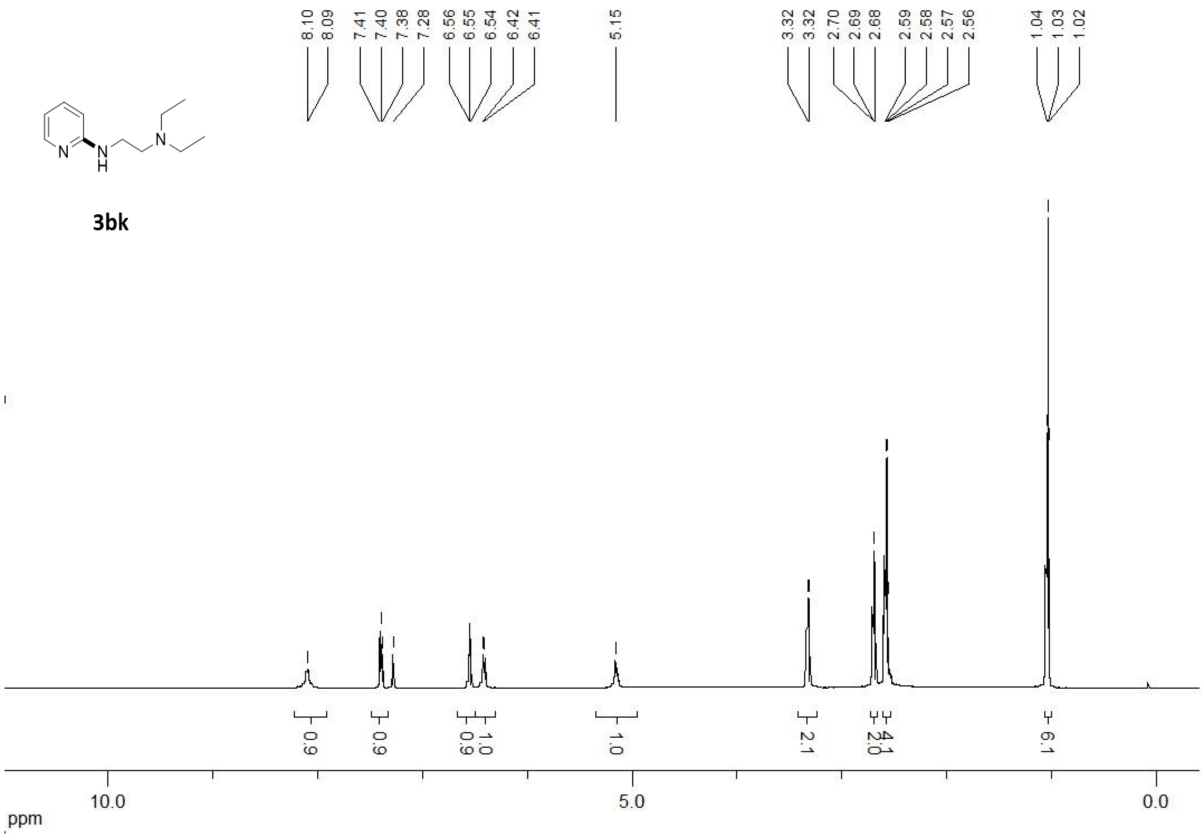

^{13}C NMR 150MHz, $CDCl_3$

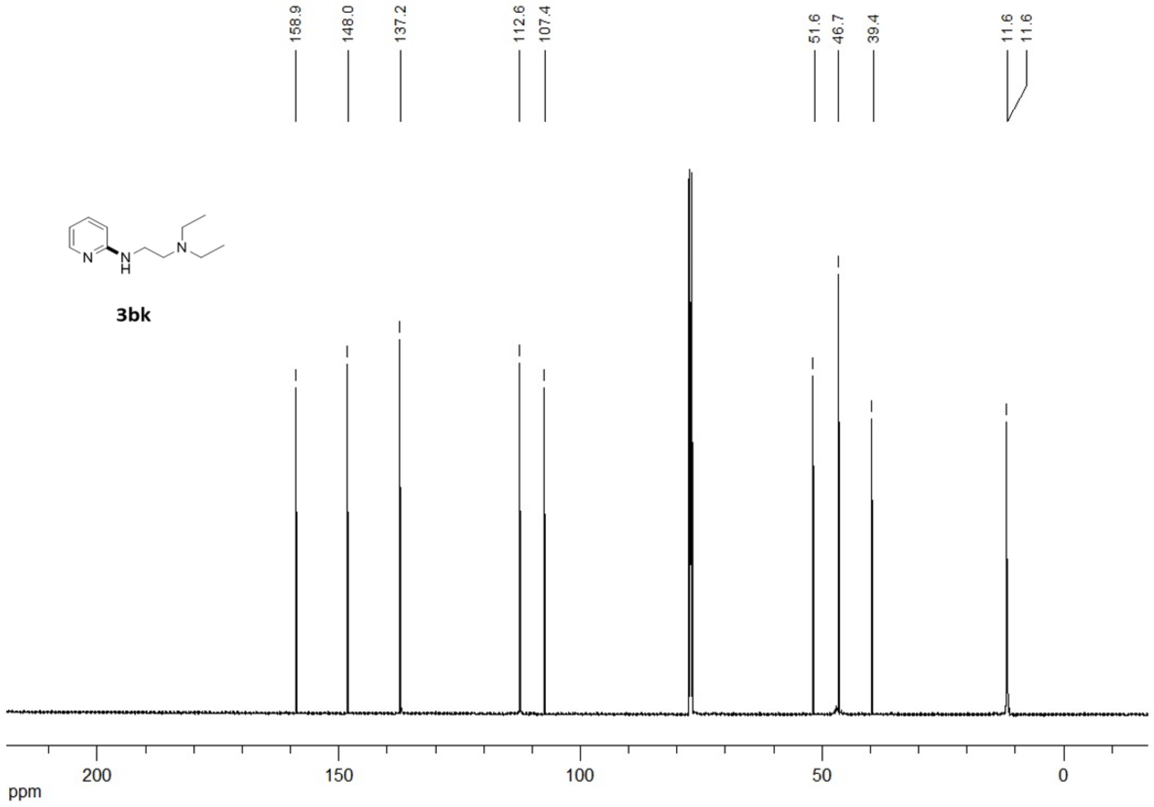

^{1}H NMR 600MHz, $CDCl_3$

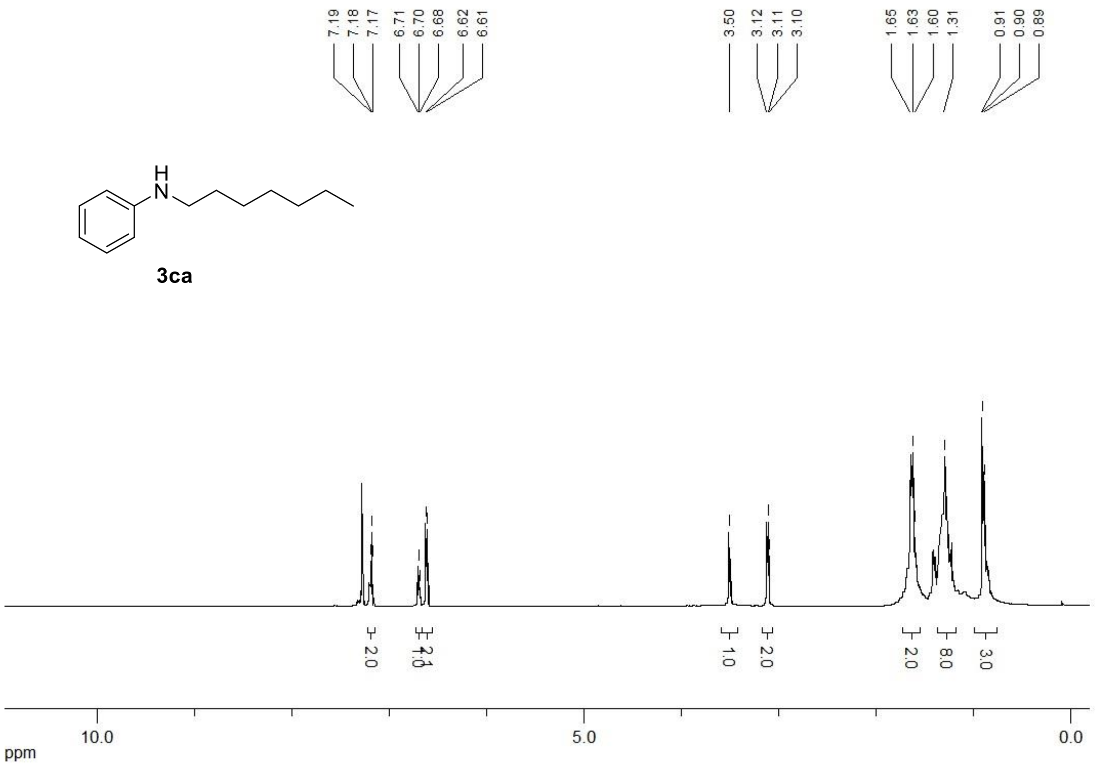

^{13}C NMR 150MHz, $CDCl_3$

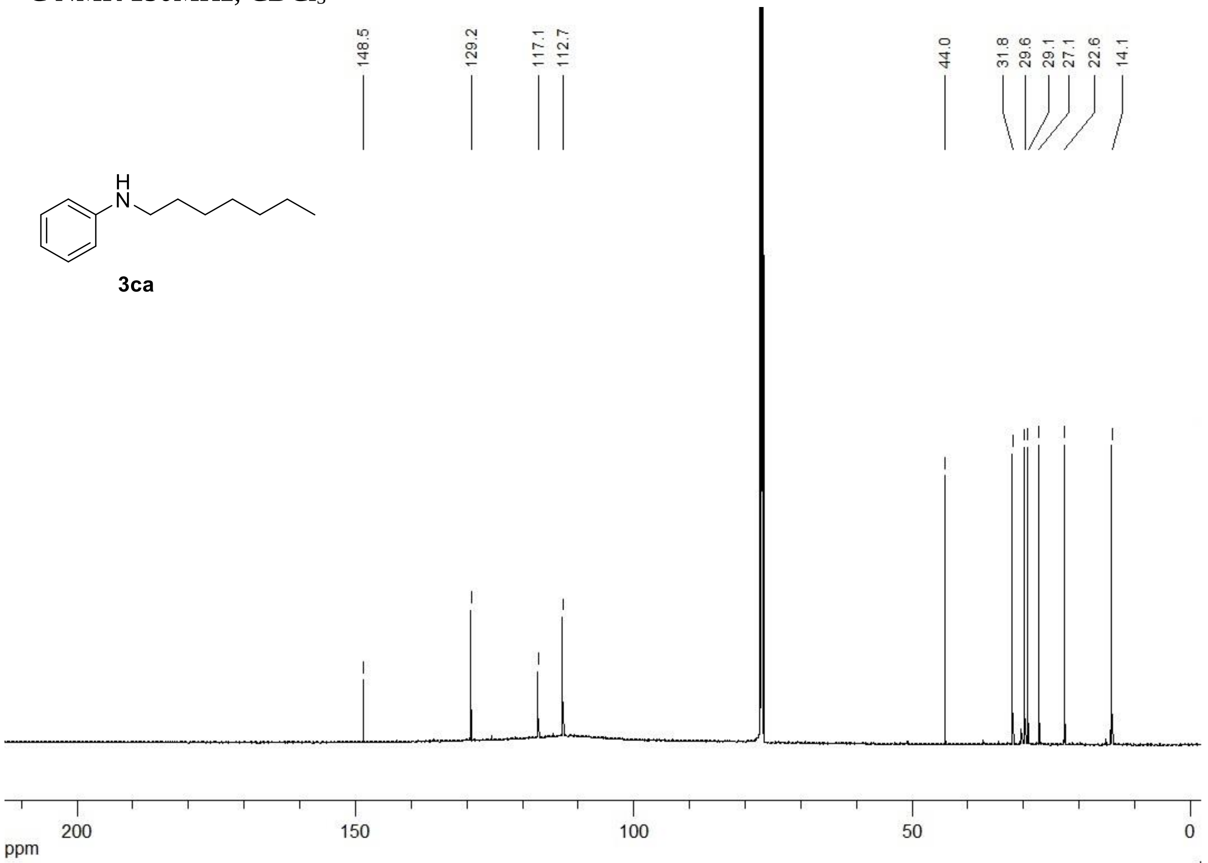

^{1}H NMR 600MHz, $CDCl_3$

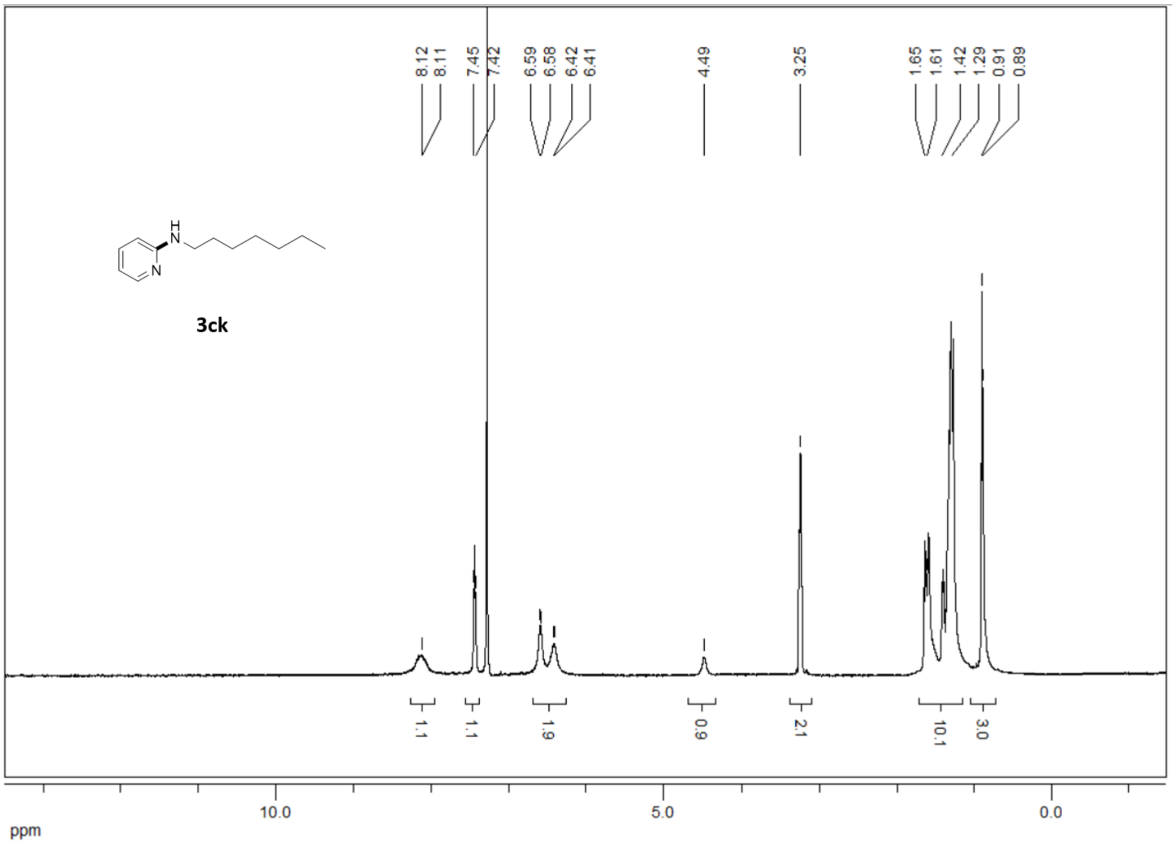

^{13}C NMR 150MHz, $CDCl_3$c

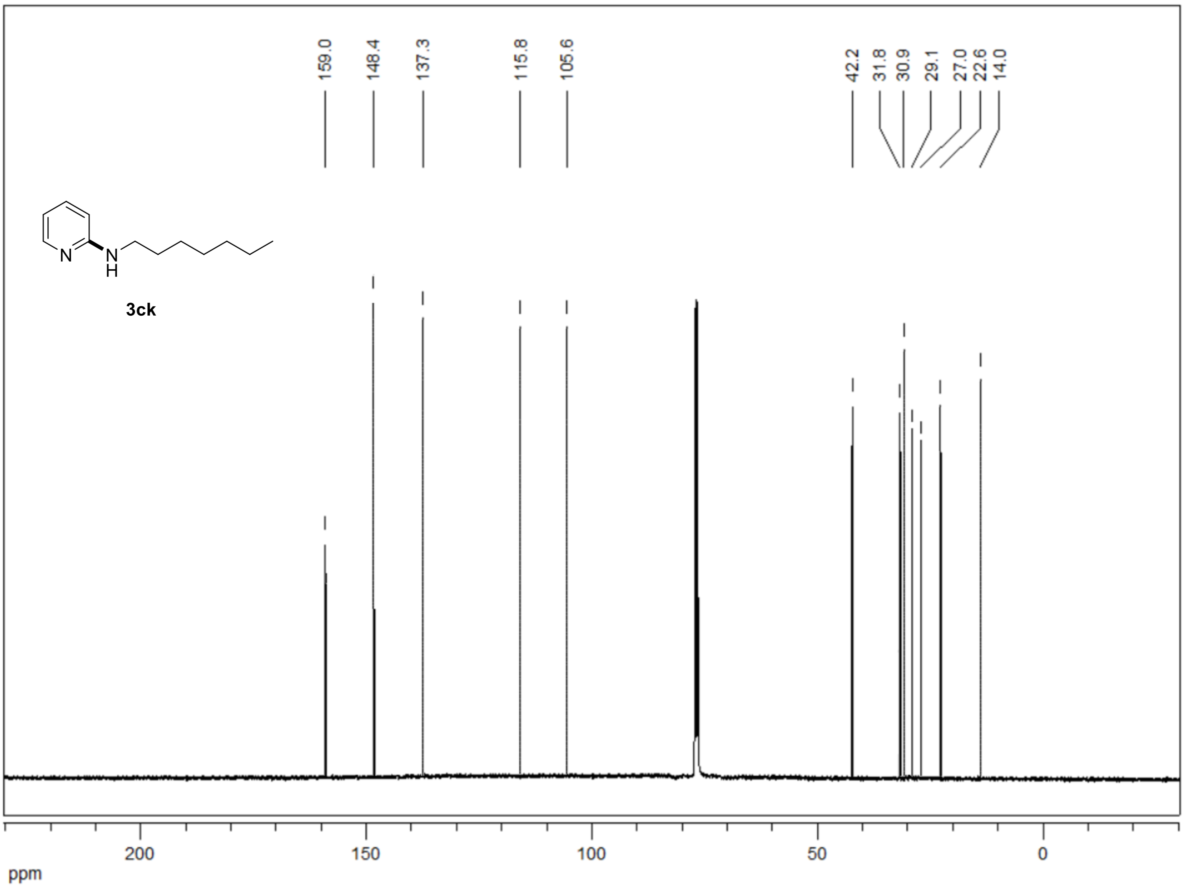

^{1}H NMR 600MHz, $CDCl_3$

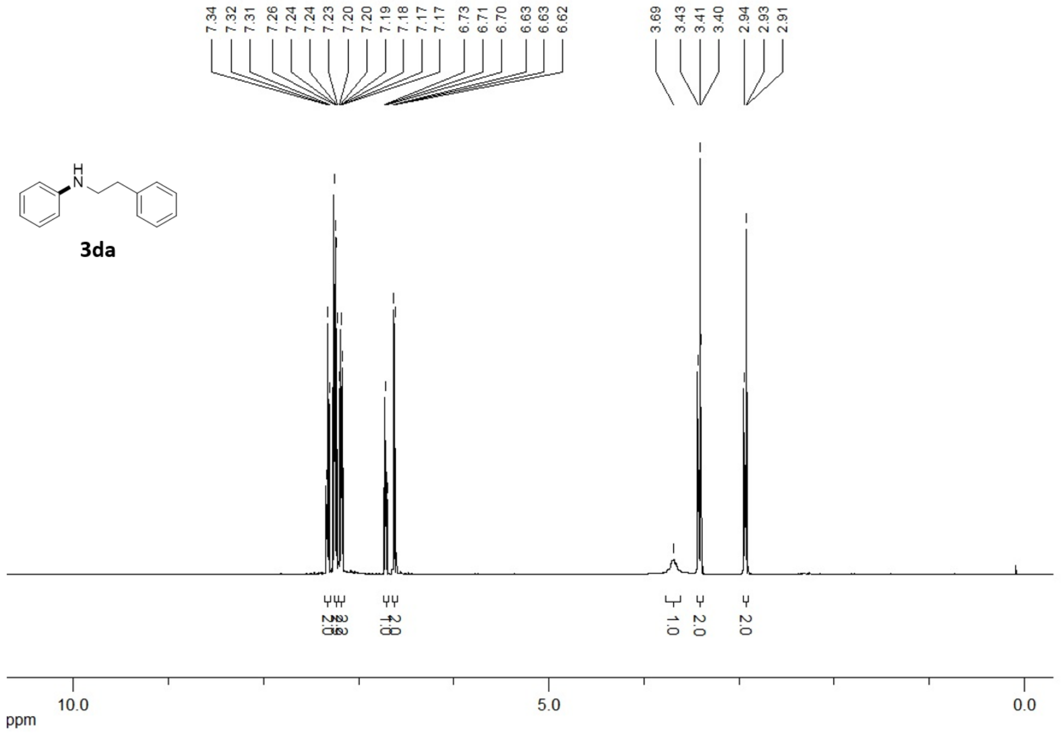

^{13}C NMR 150MHz, $CDCl_3$

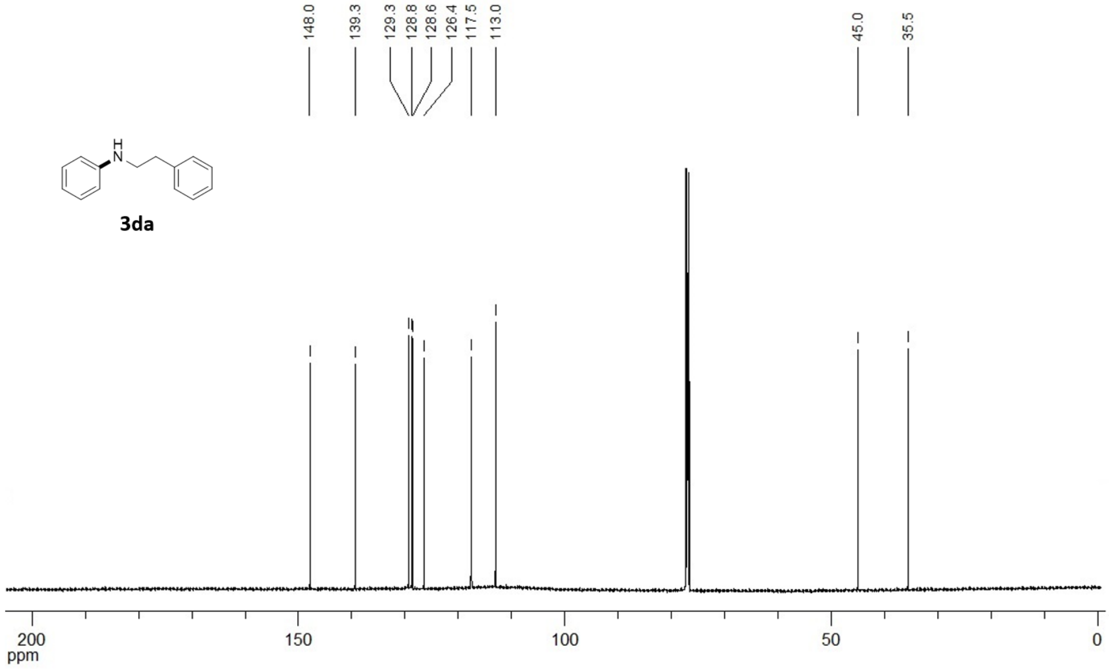

^{1}H NMR 600MHz, $CDCl_3$

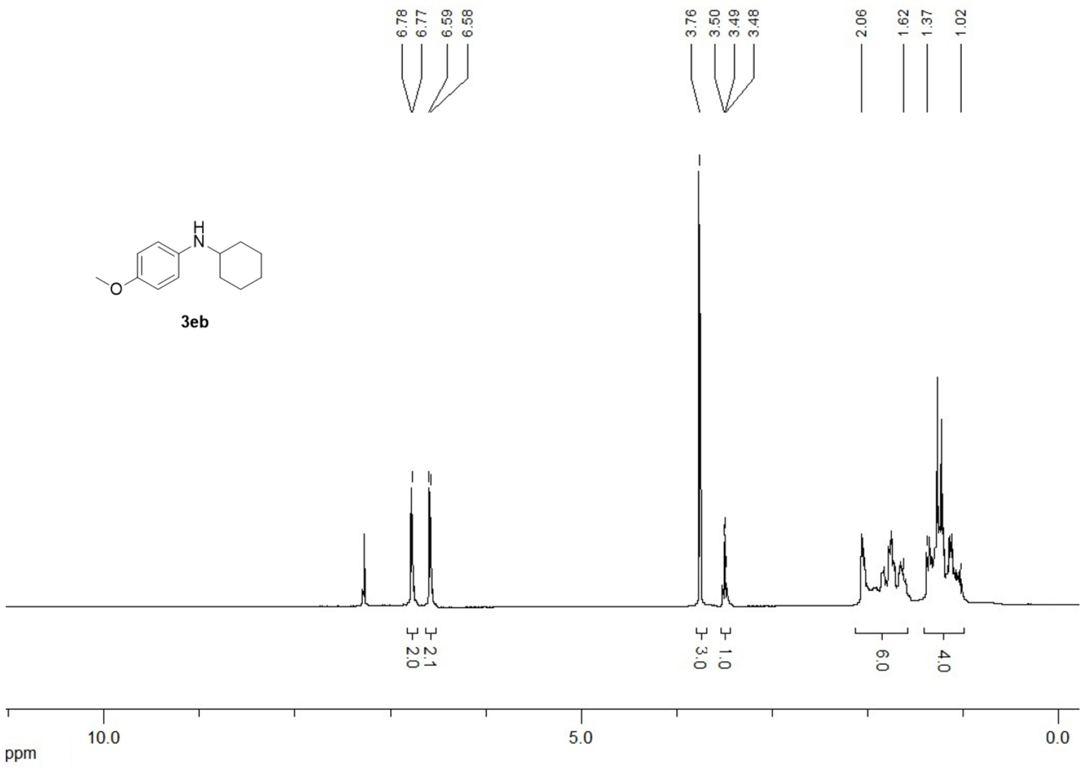

^{13}C NMR 150MHz, $CDCl_3$

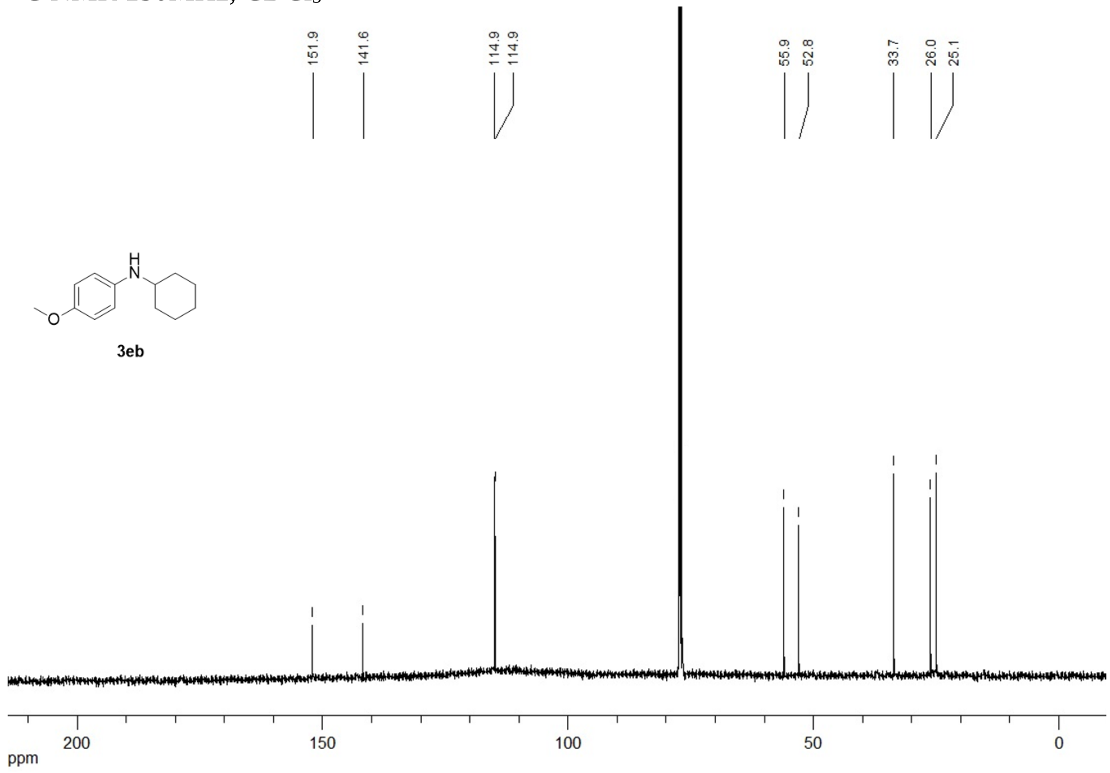

^{1}H NMR 600MHz, $CDCl_3$

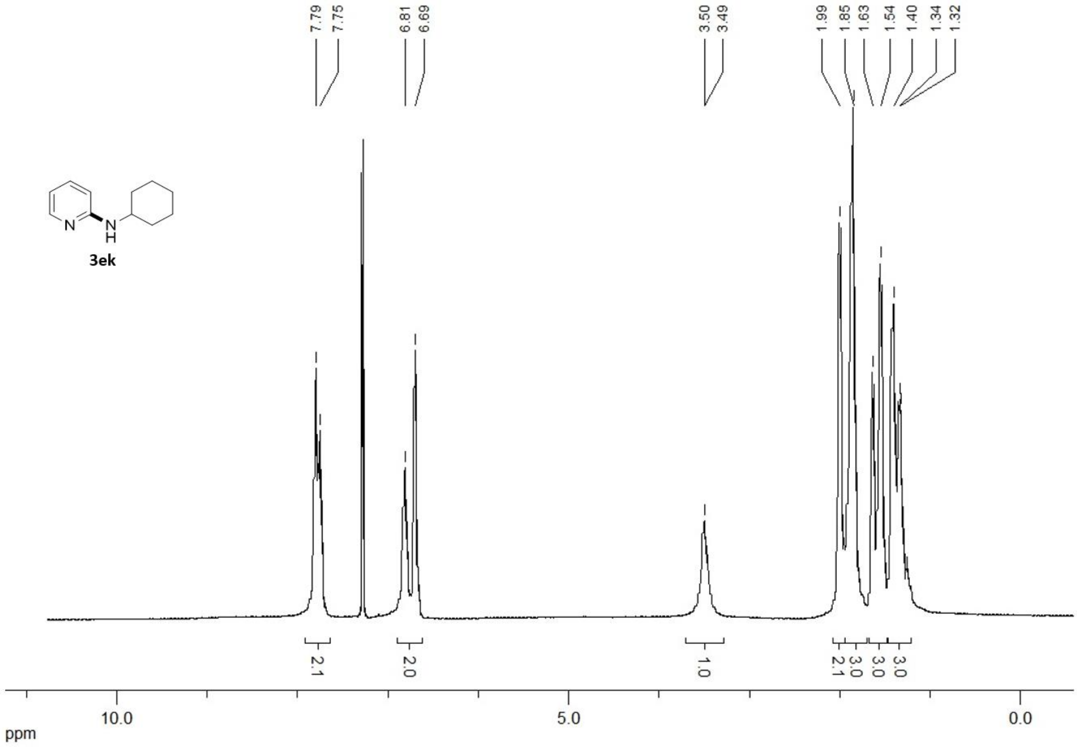

^{13}C NMR 150MHz, $CDCl_3$

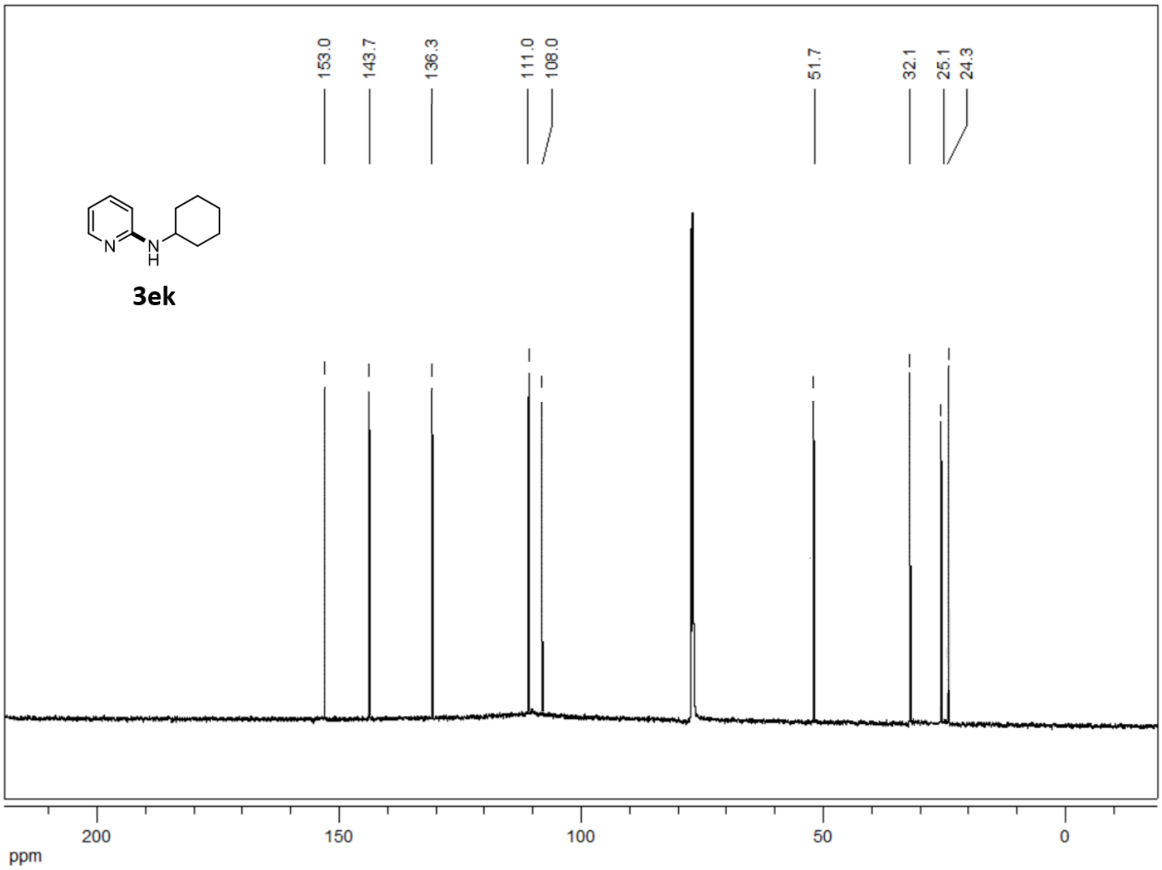

^{1}H NMR 600MHz, $CDCl_3$

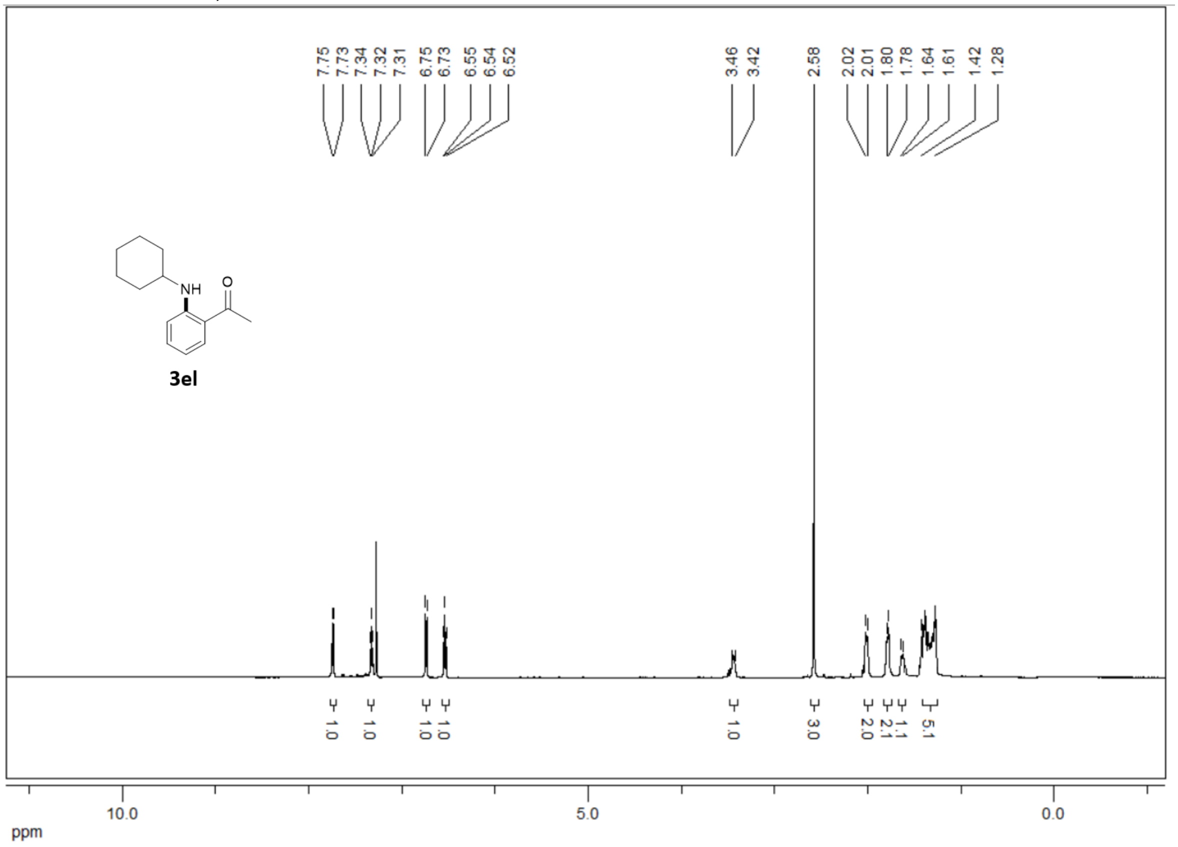

^{13}C NMR 150MHz, $CDCl_3$

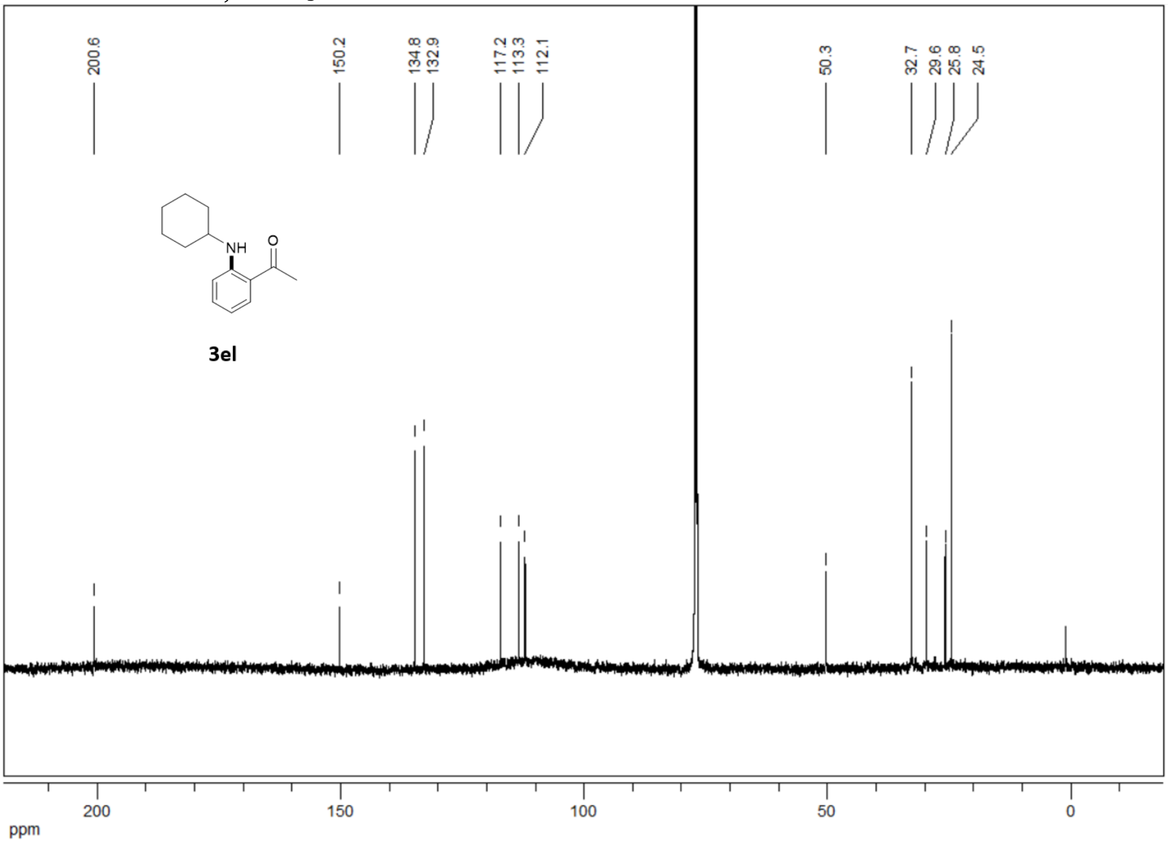

¹H NMR 600MHz, CDCl$_3$

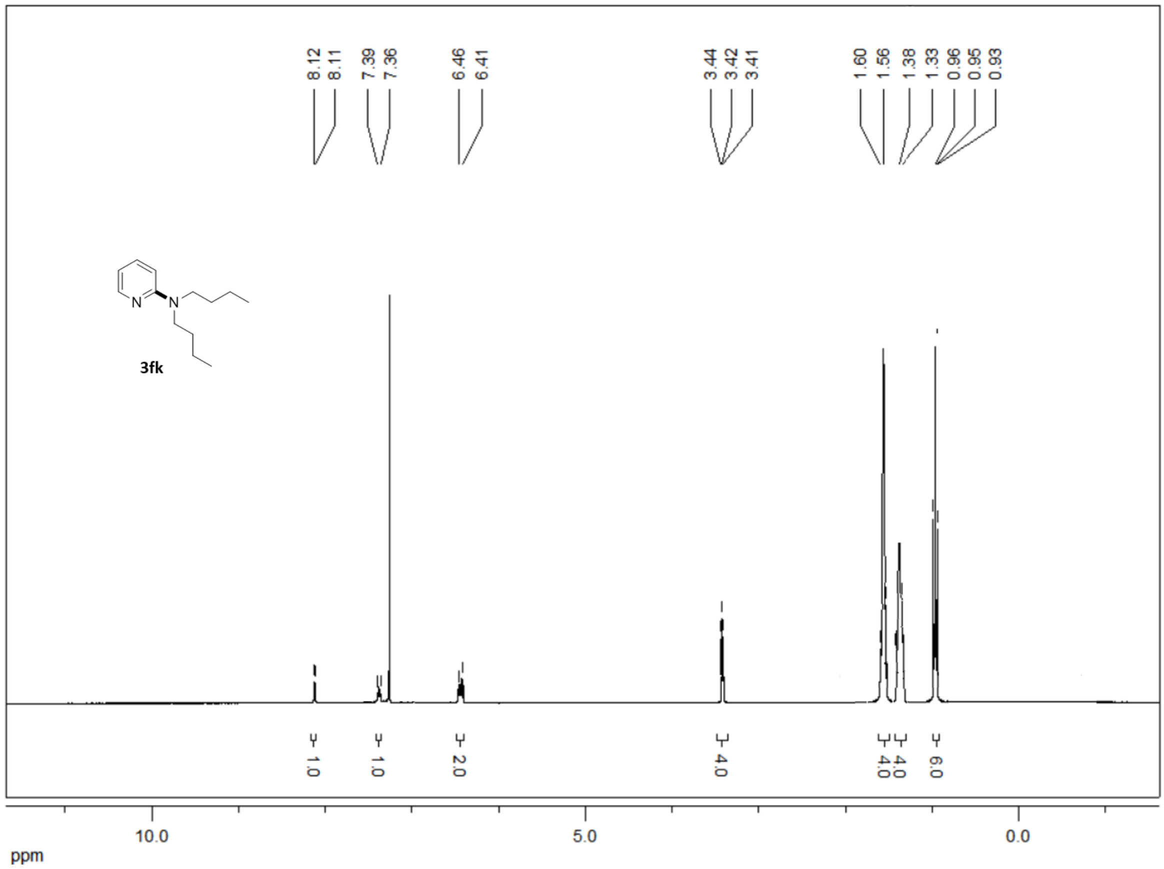

^{13}C NMR 150MHz, CDCl$_3$

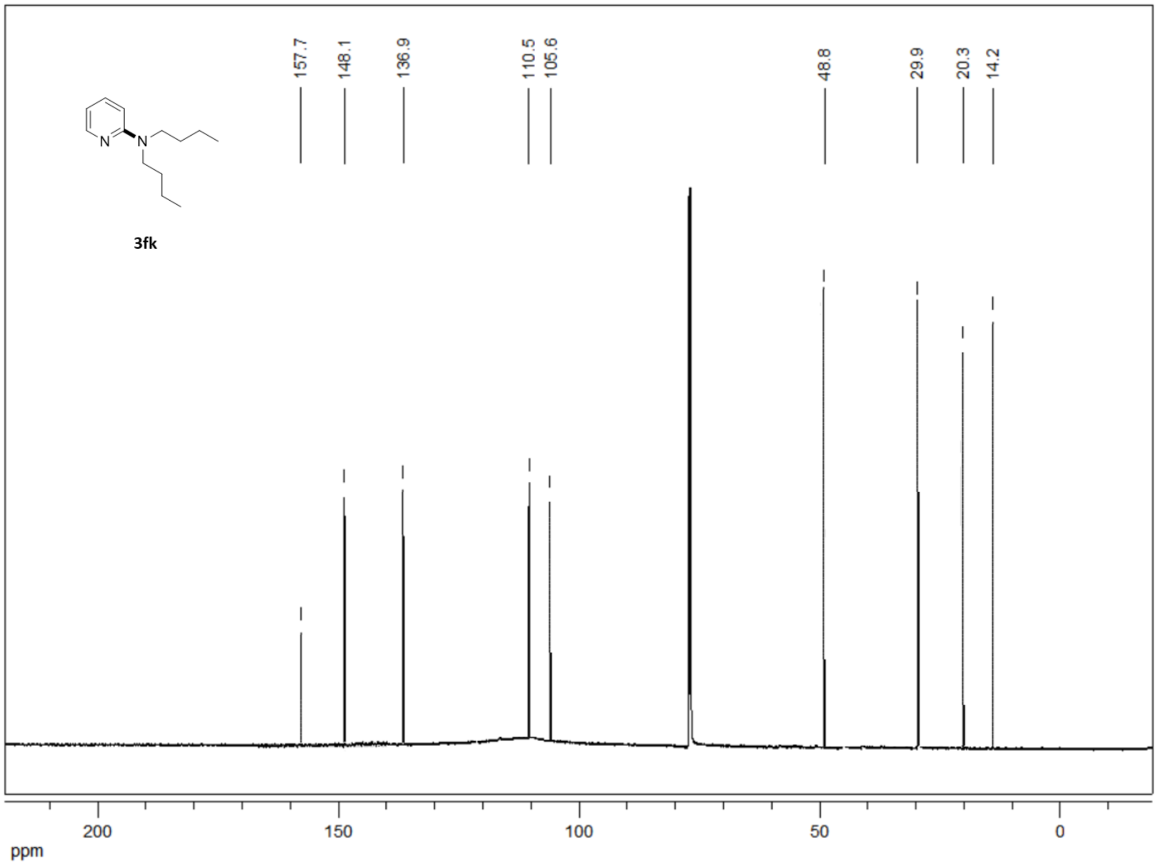

^{1}H NMR 600MHz, $CDCl_3$

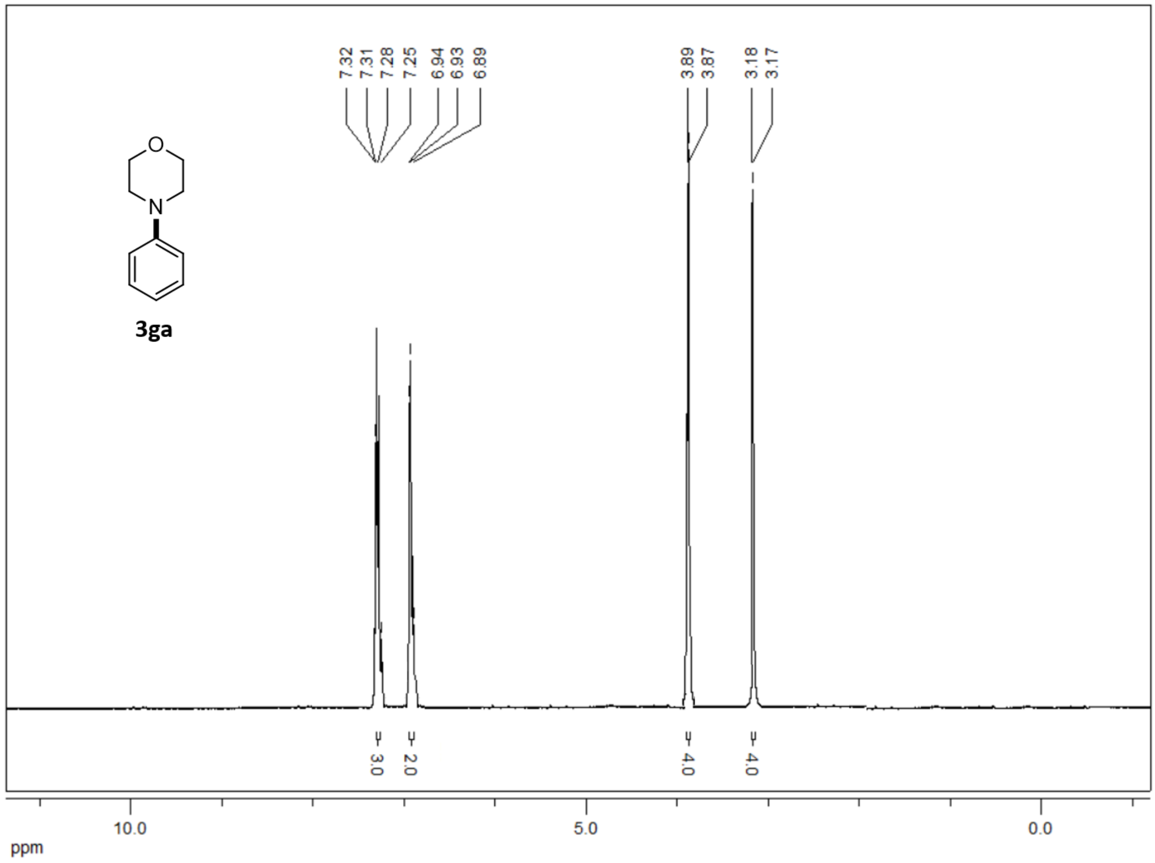

^{13}C NMR 150MHz, $CDCl_3$

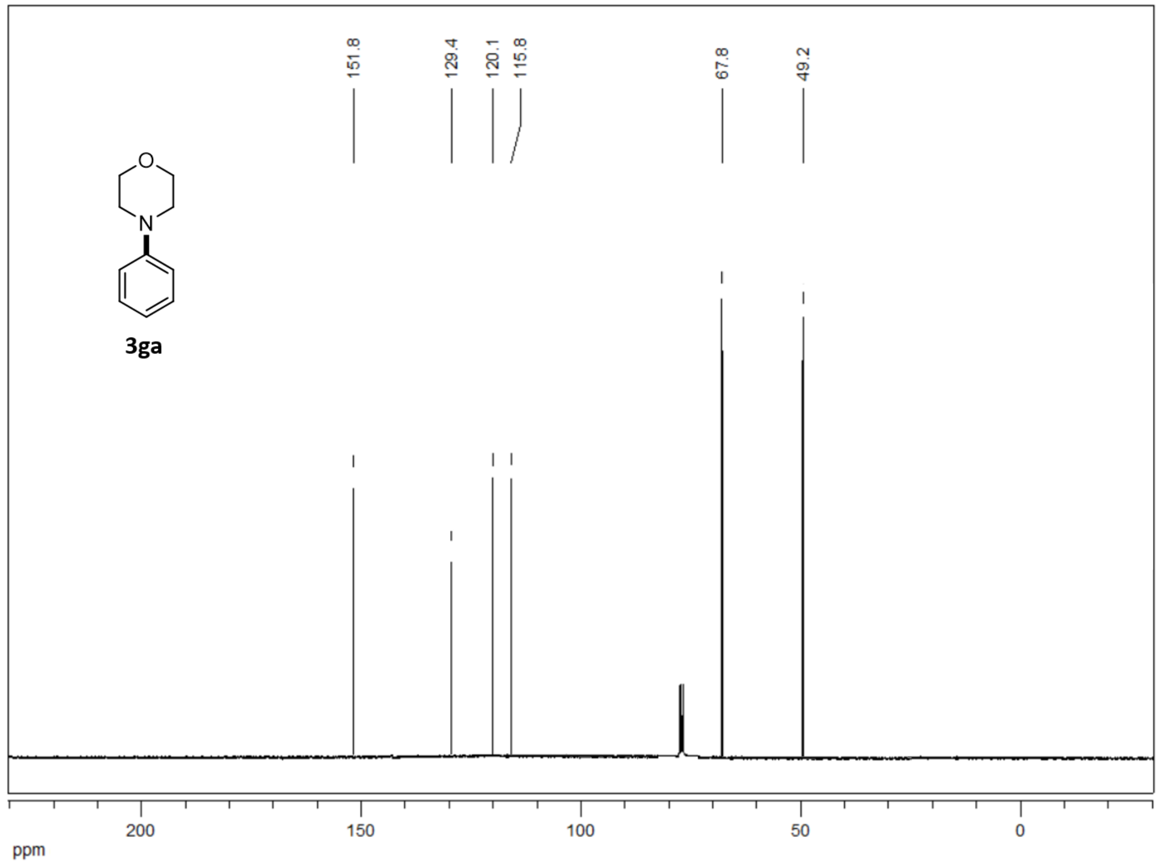

^{1}H NMR 600MHz, $CDCl_3$

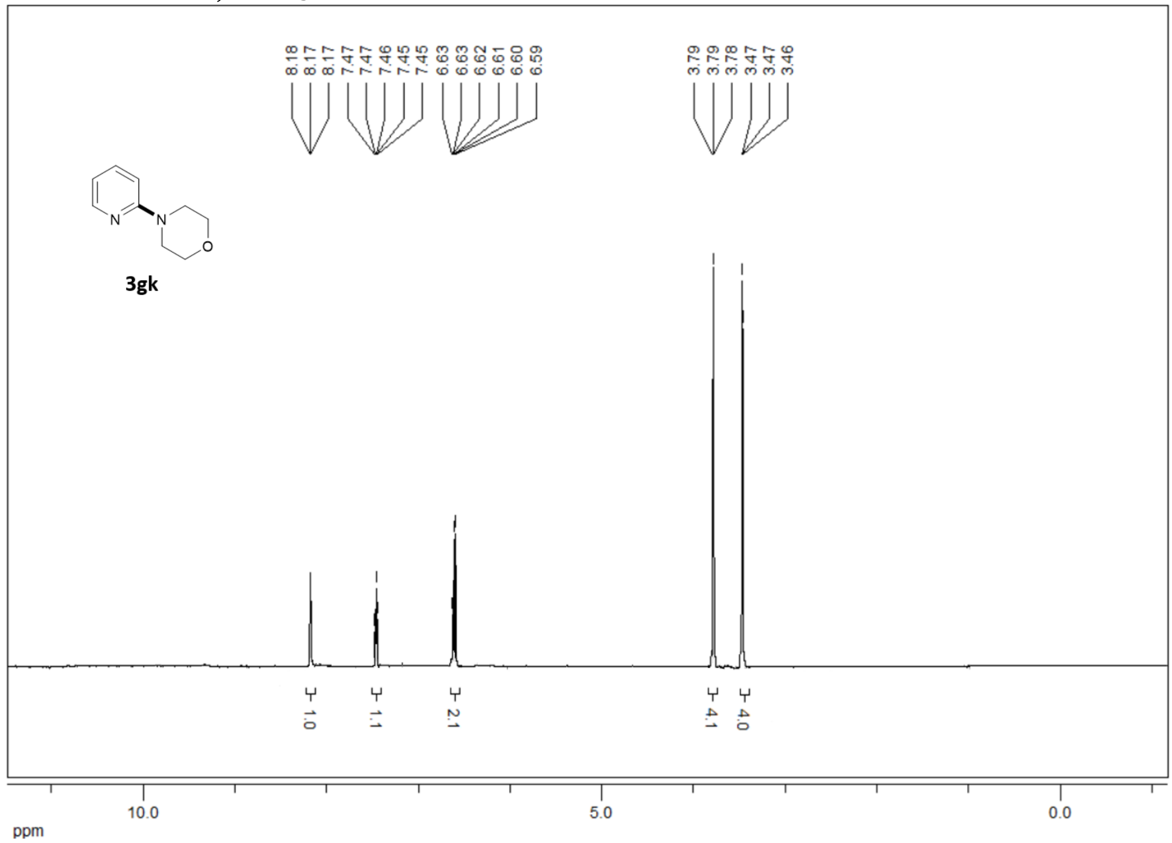

^{13}C NMR 150MHz, $CDCl_3$

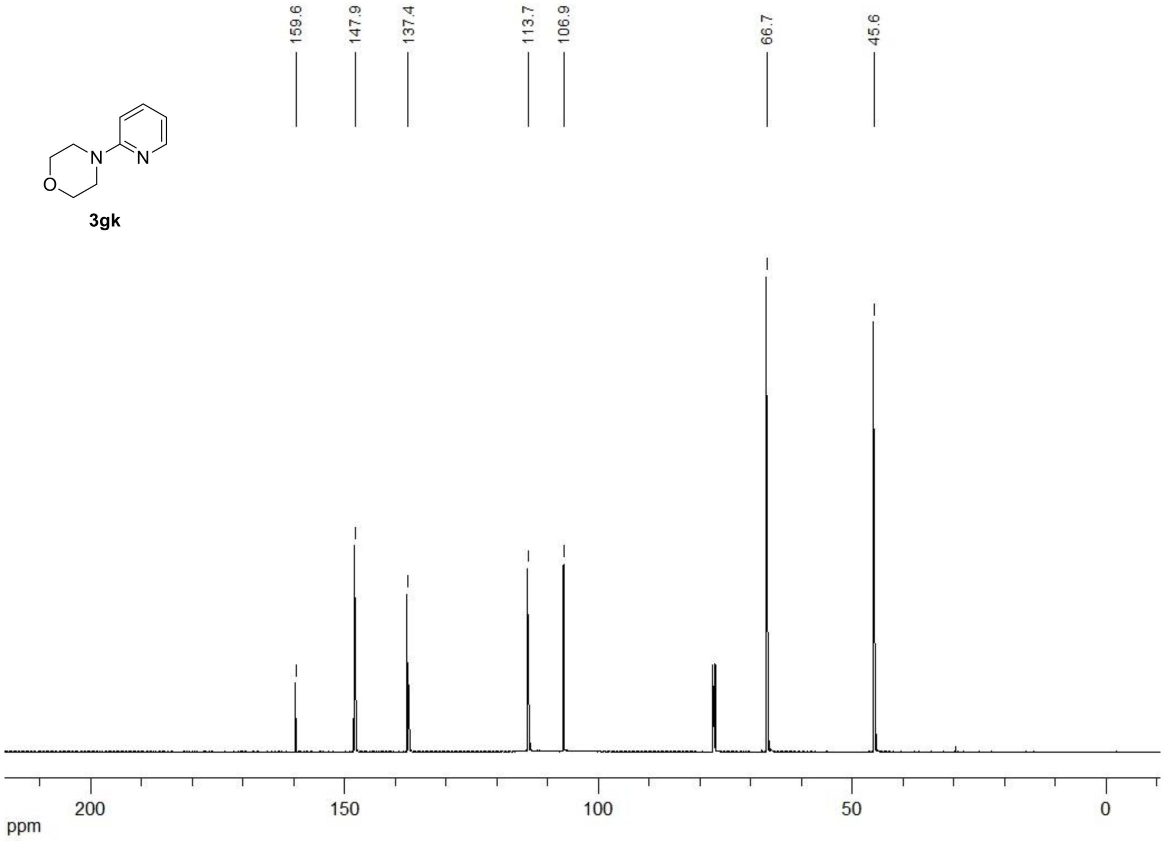

^{1}H NMR 600MHz, $CDCl_3$

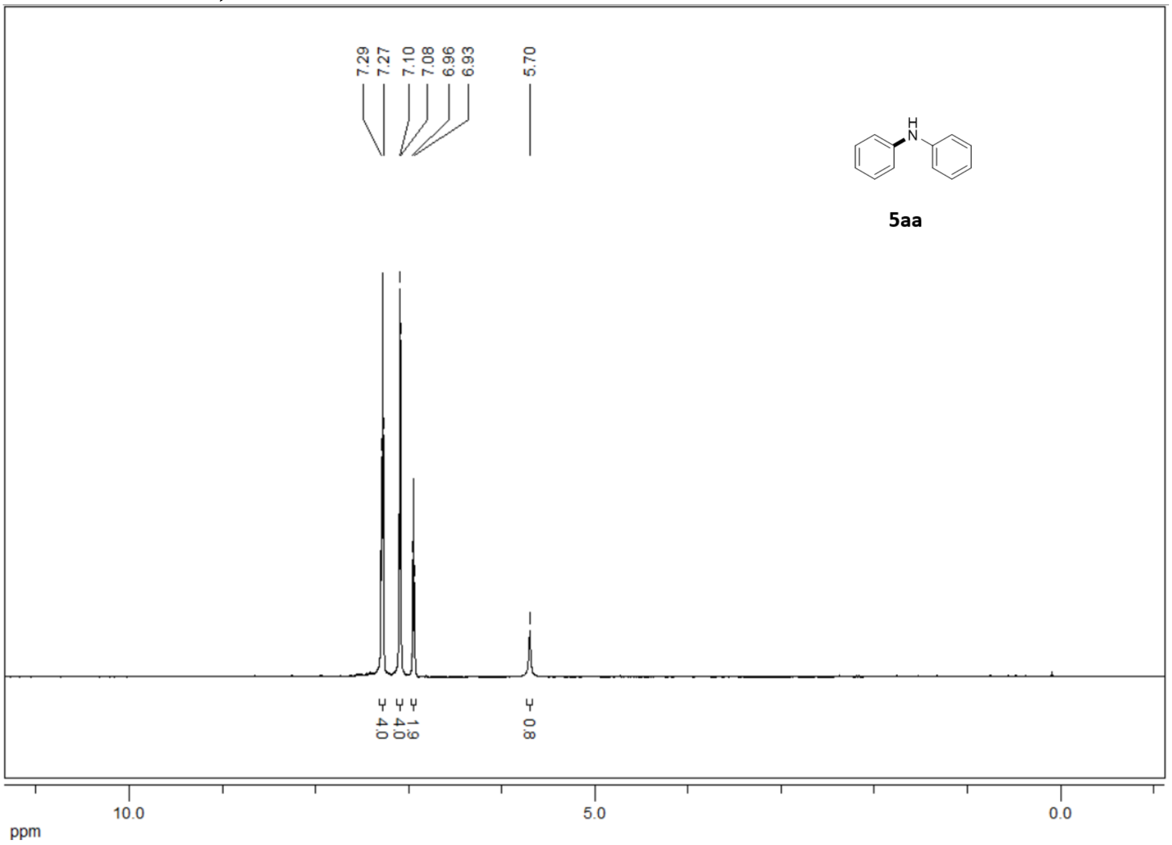

^{13}C NMR 150MHz, $CDCl_3$

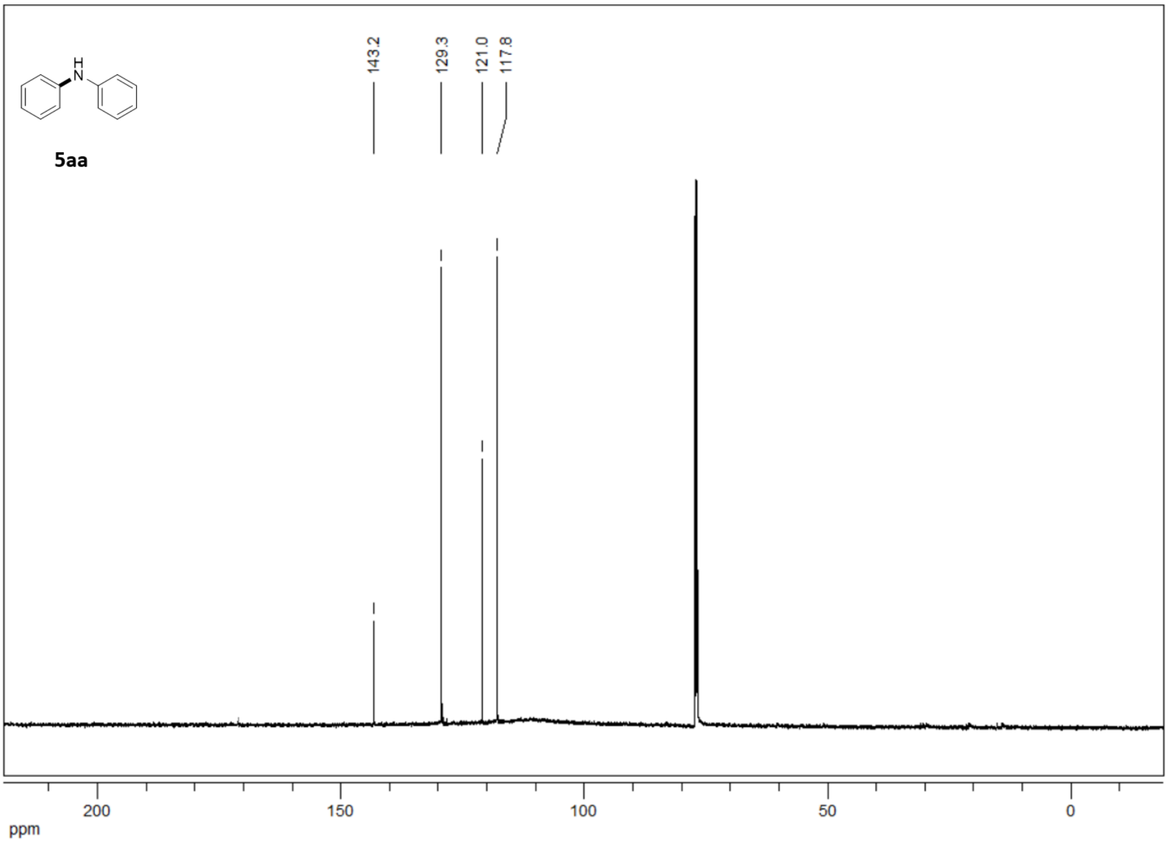

^{1}H NMR 600MHz, $CDCl_3$

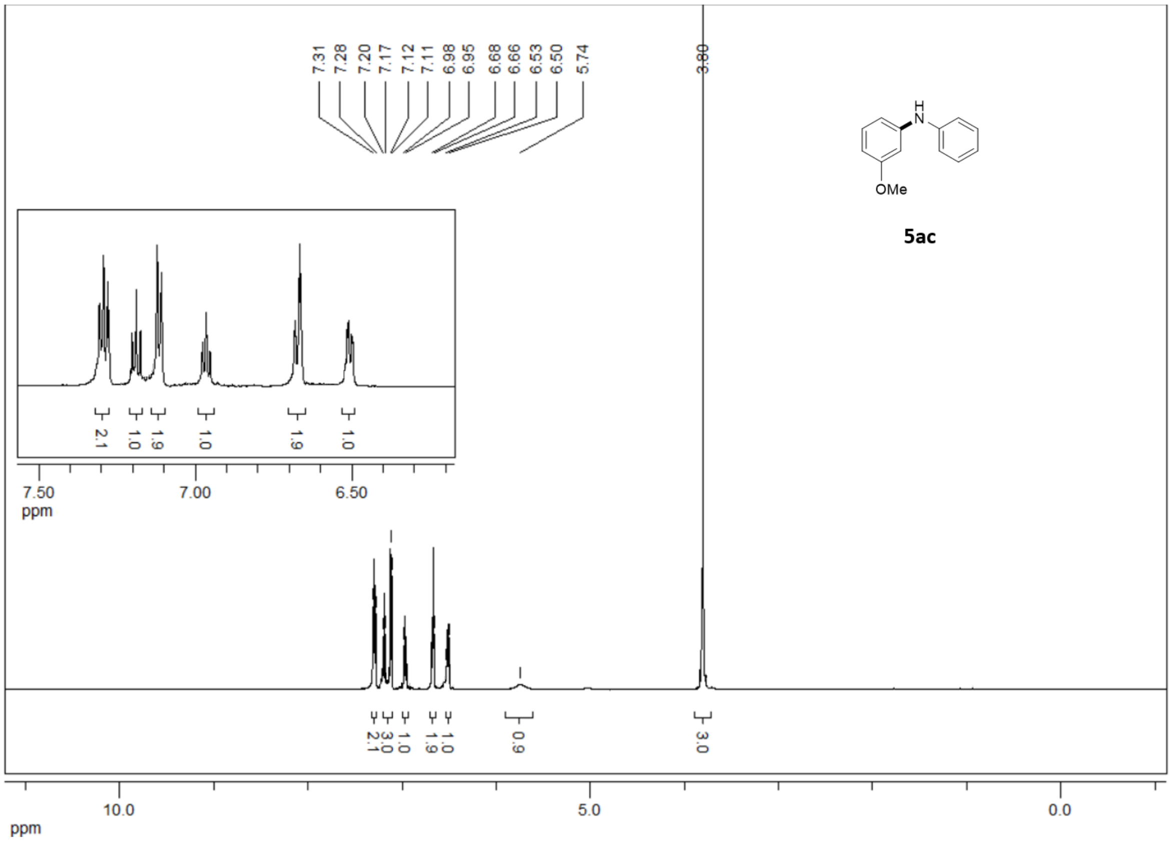

^{13}C NMR 150MHz, $CDCl_3$

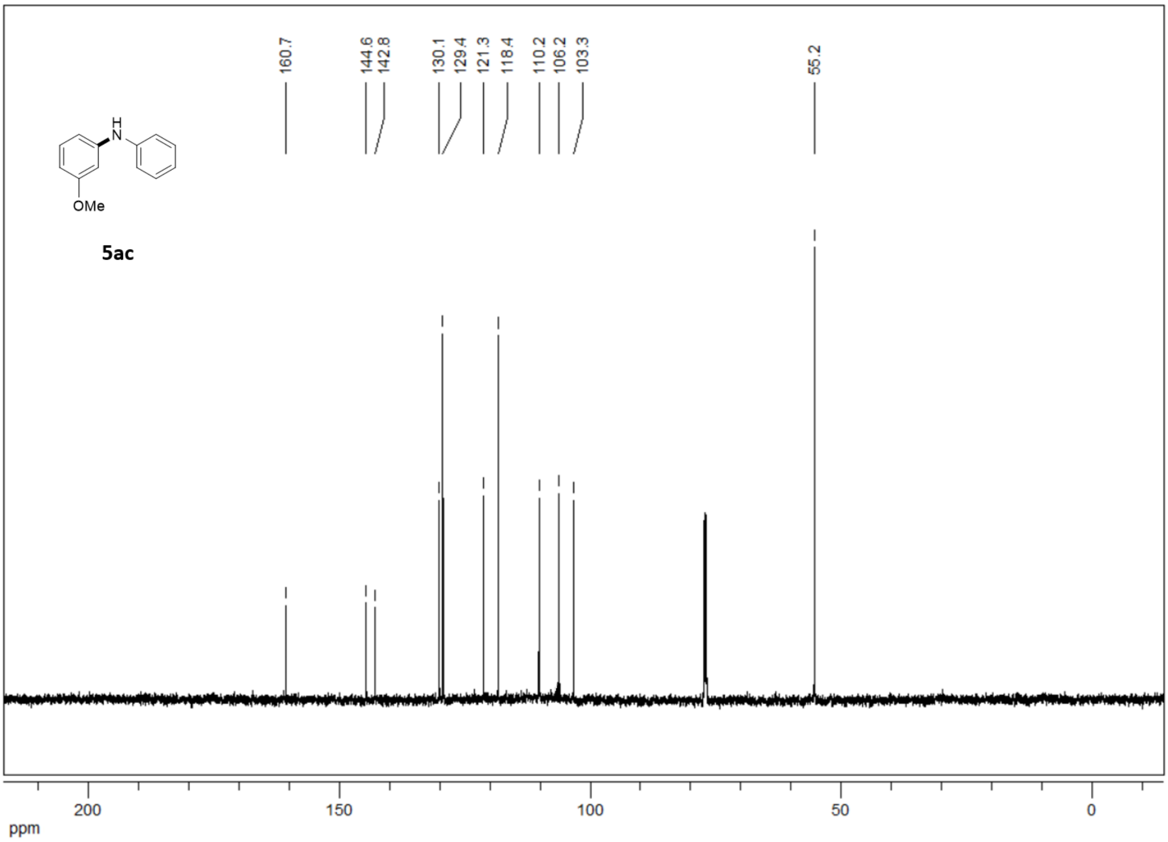

^{1}H NMR 600MHz, $CDCl_3$

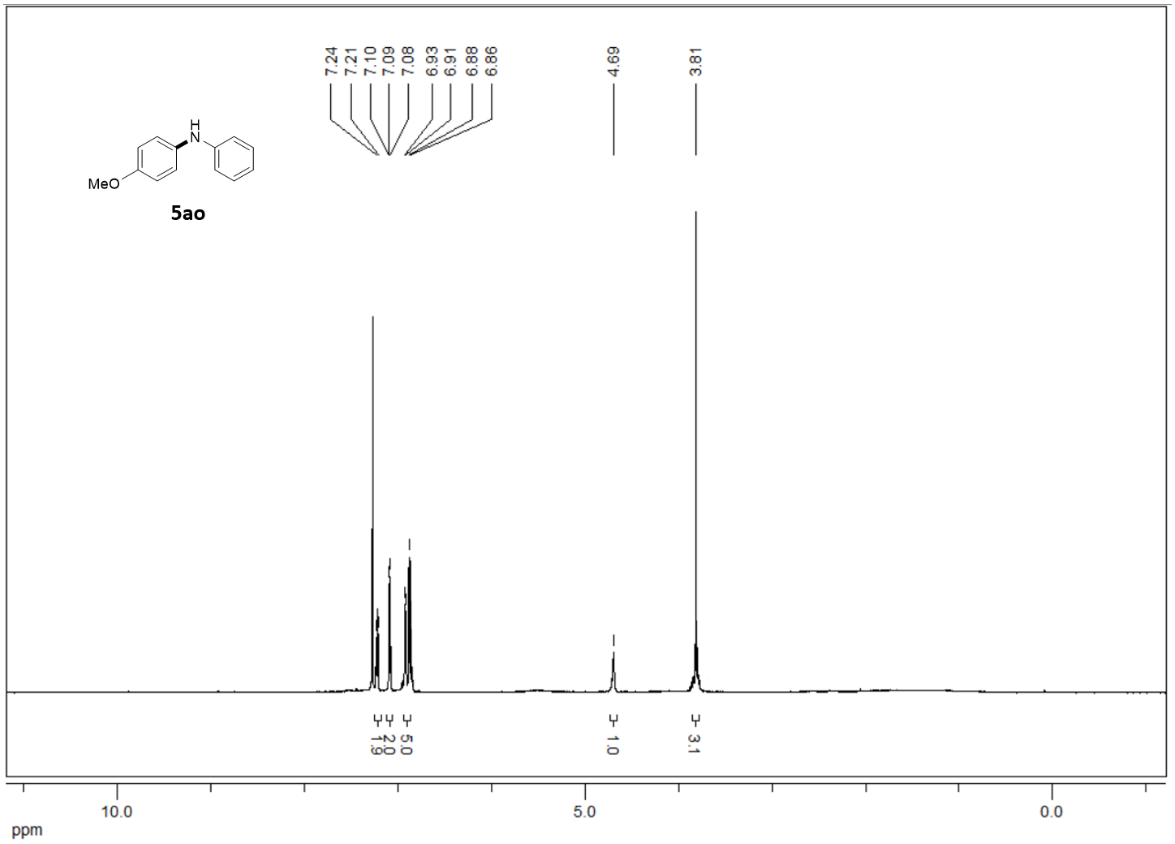

^{13}C NMR 150MHz, $CDCl_3$

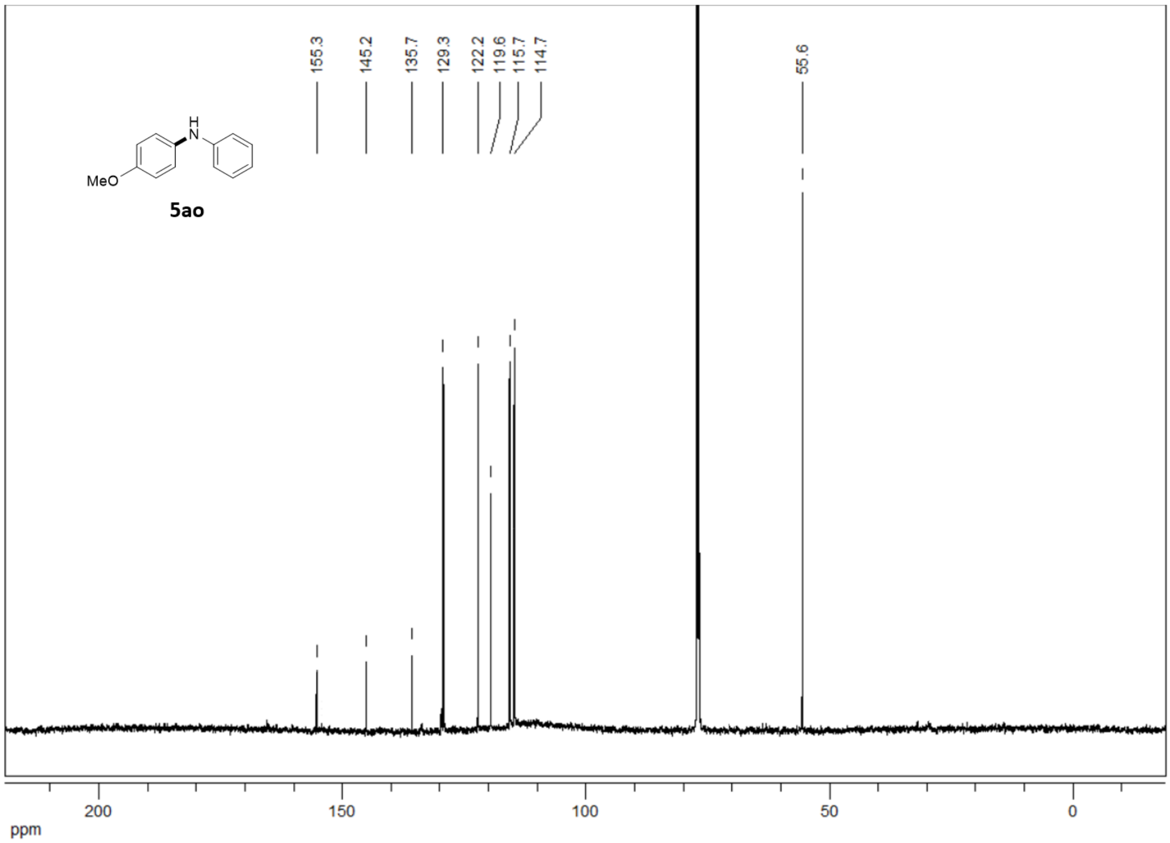

^{1}H NMR 600MHz, $CDCl_3$

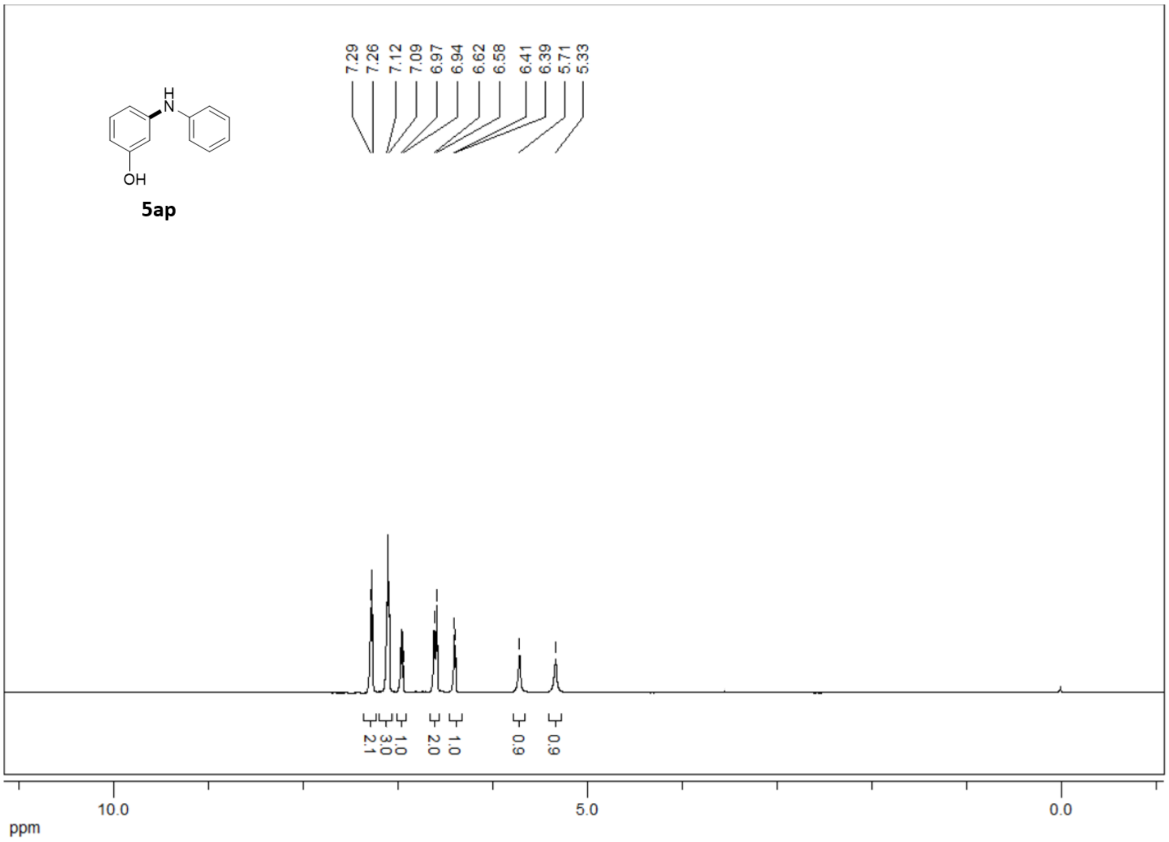

^{13}C NMR 150MHz, $CDCl_3$

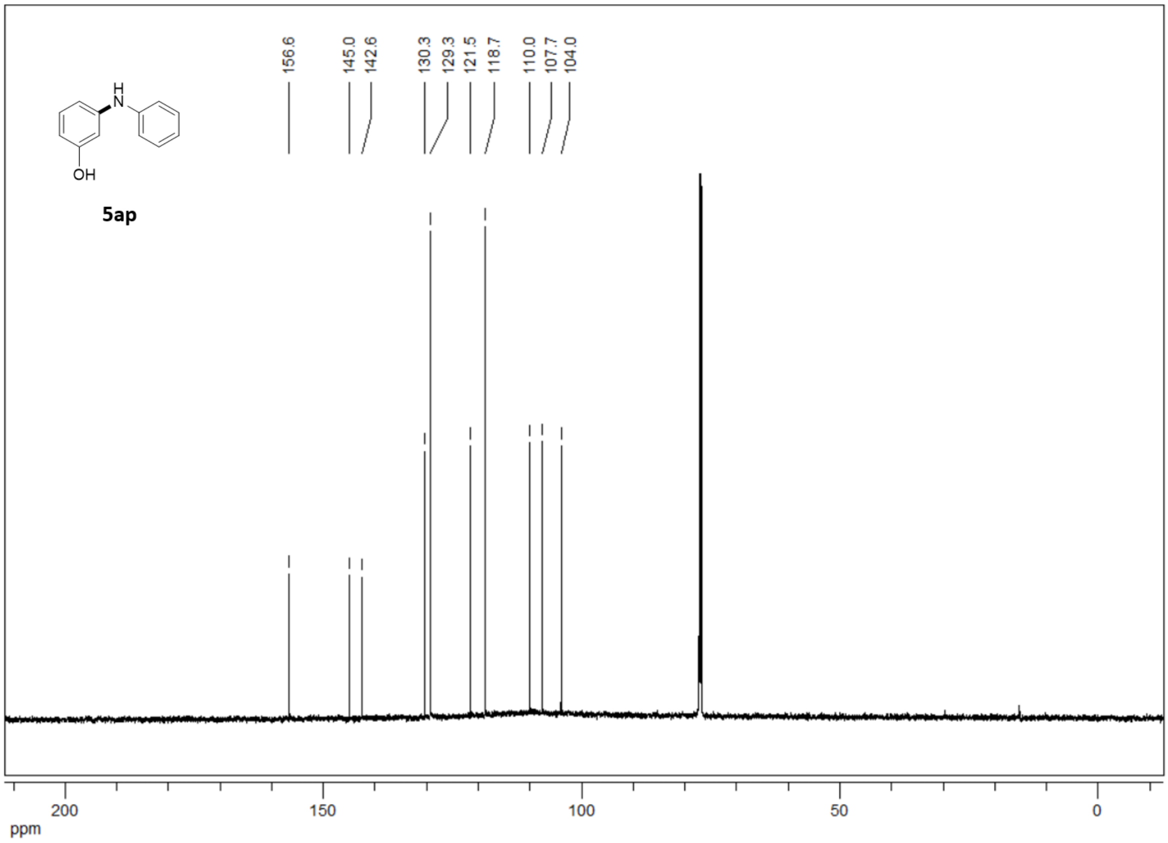

^{1}H NMR 600MHz, $CDCl_3$

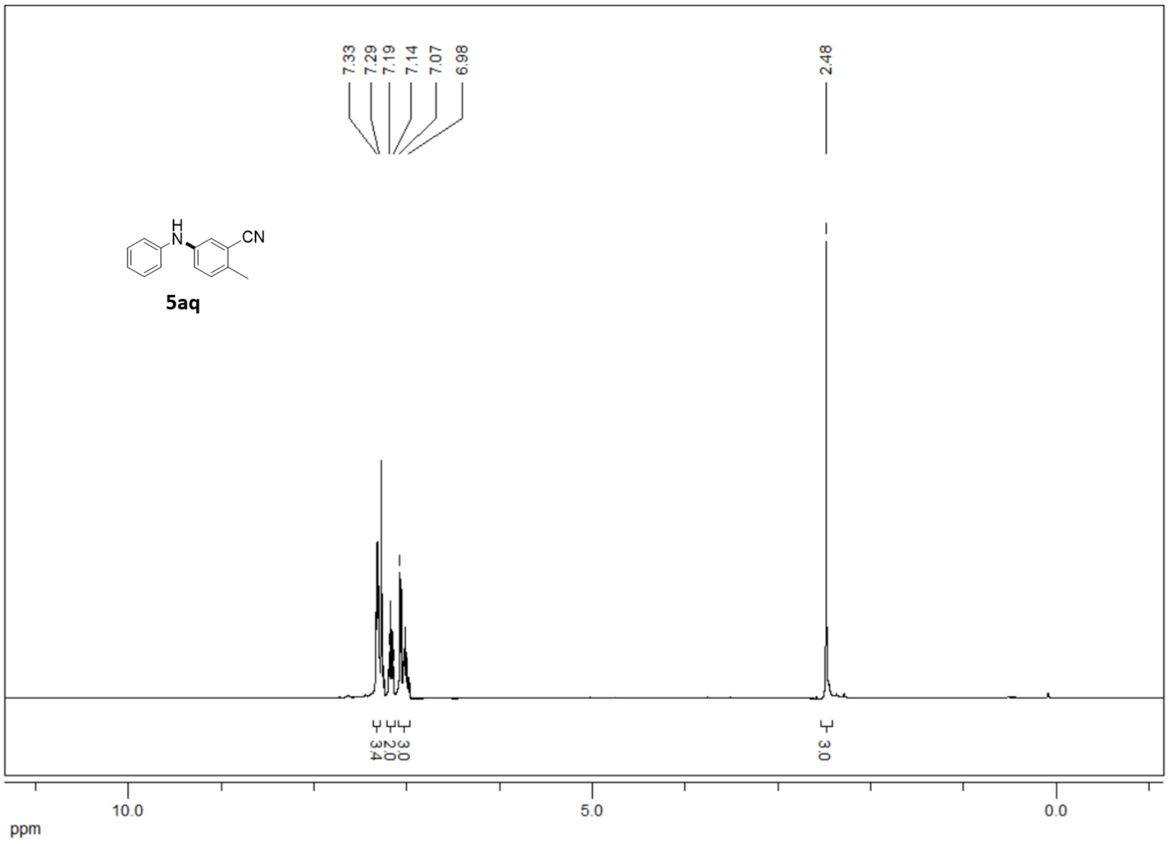

^{13}C NMR 150MHz, $CDCl_3$

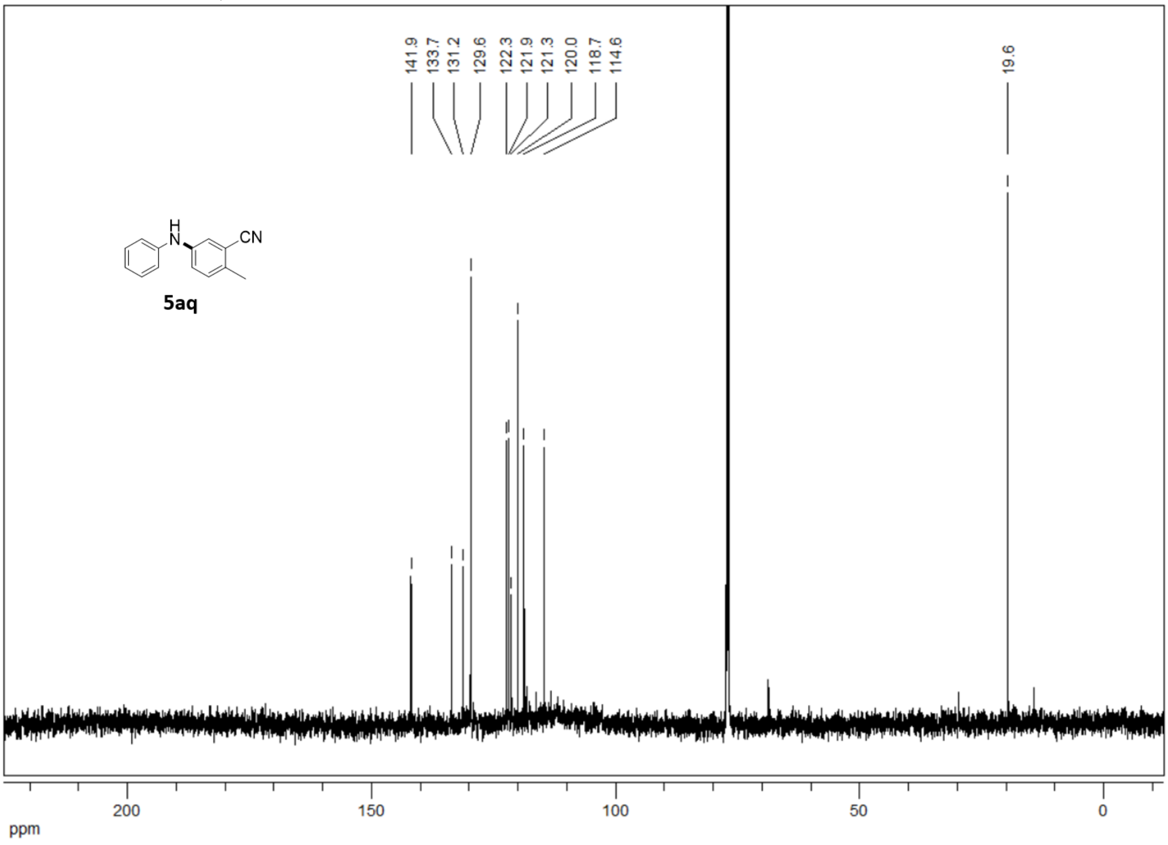

^{1}H NMR 600MHz, $CDCl_3$

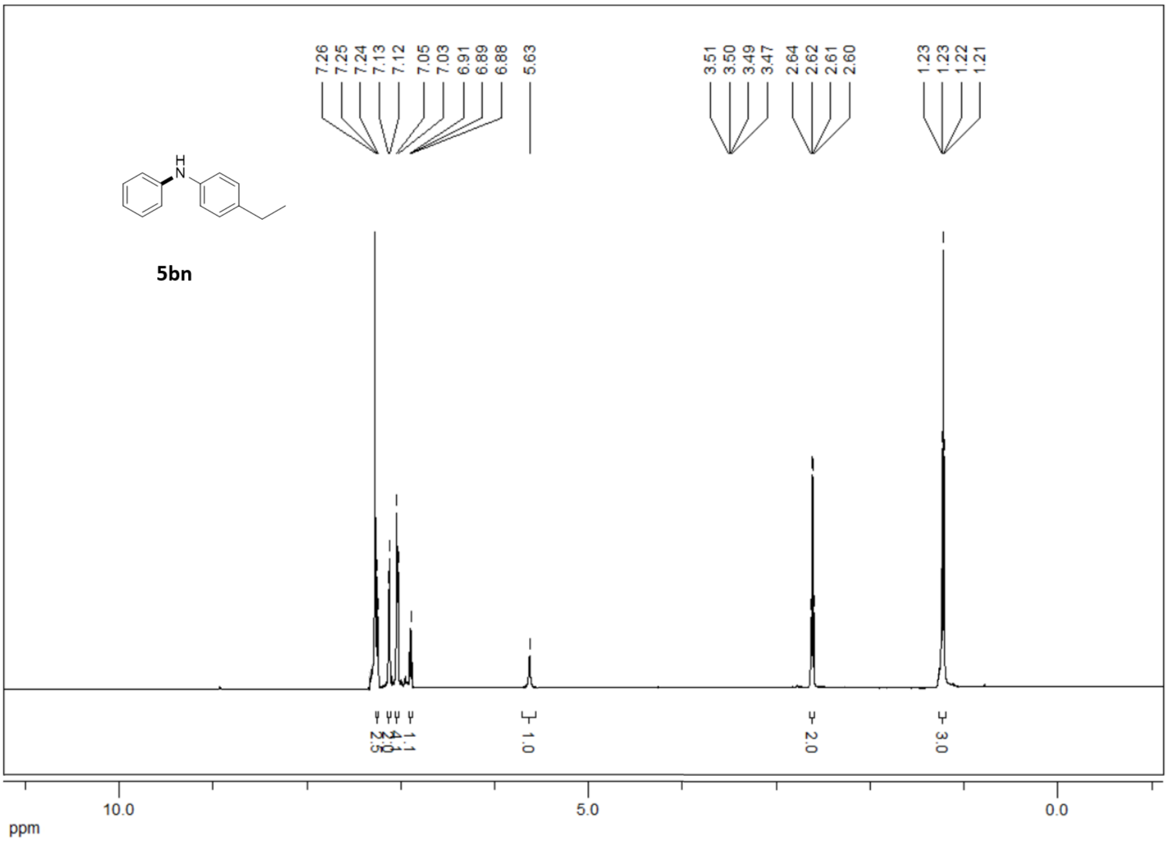

^{13}C NMR 150MHz, $CDCl_3$

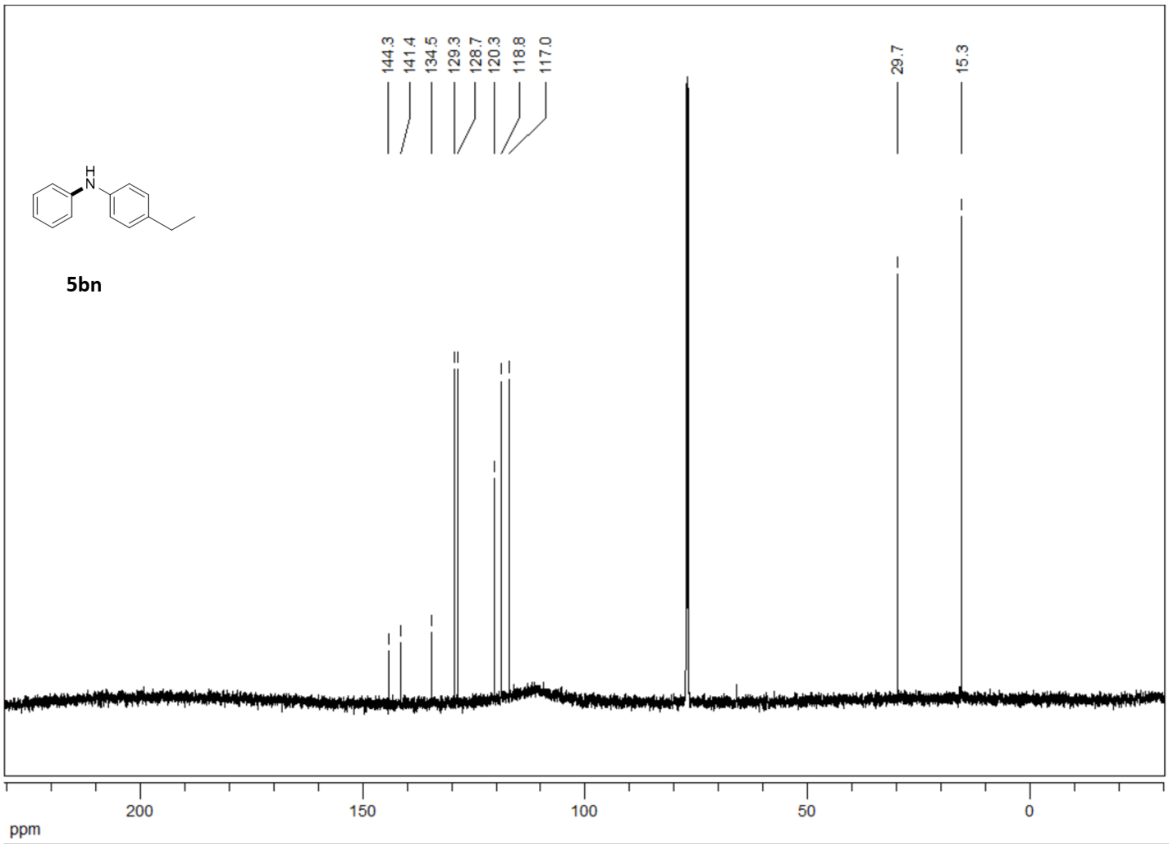

^{1}H NMR 600MHz, $CDCl_3$

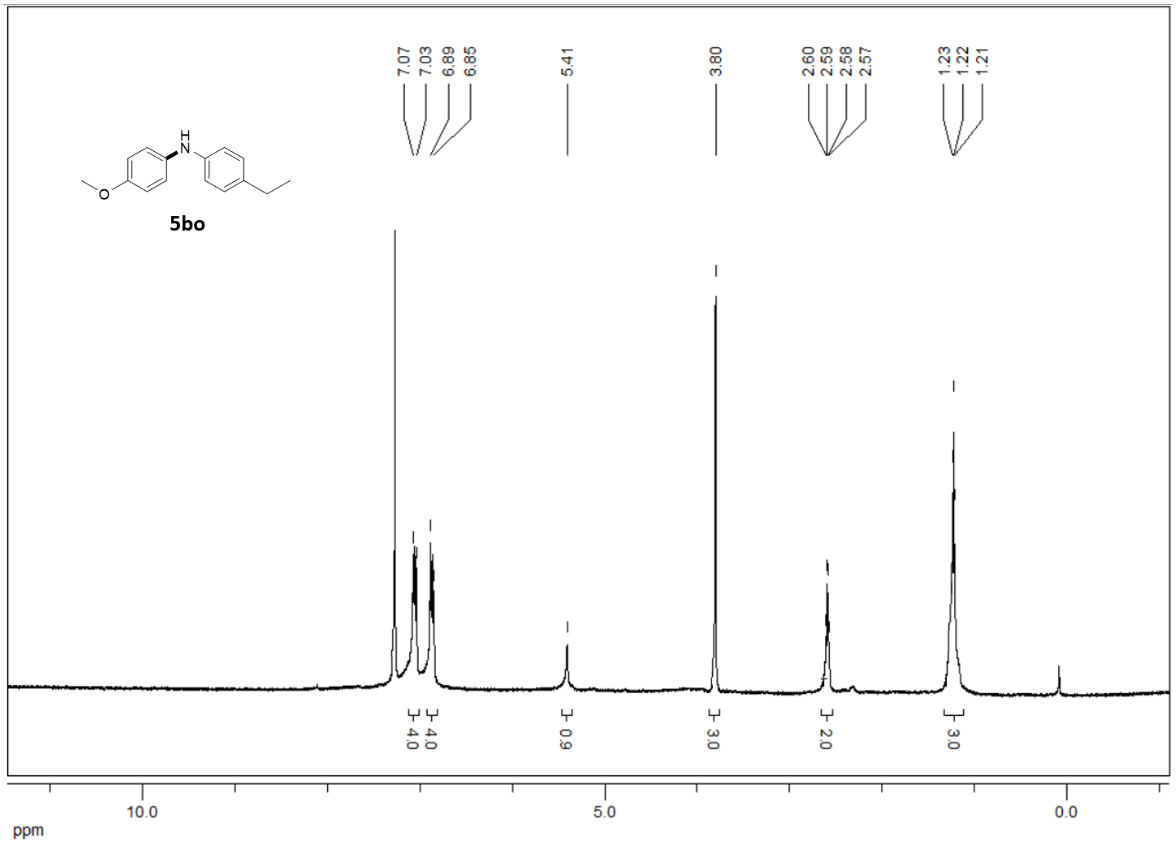

^{13}C NMR 150MHz, $CDCl_3$

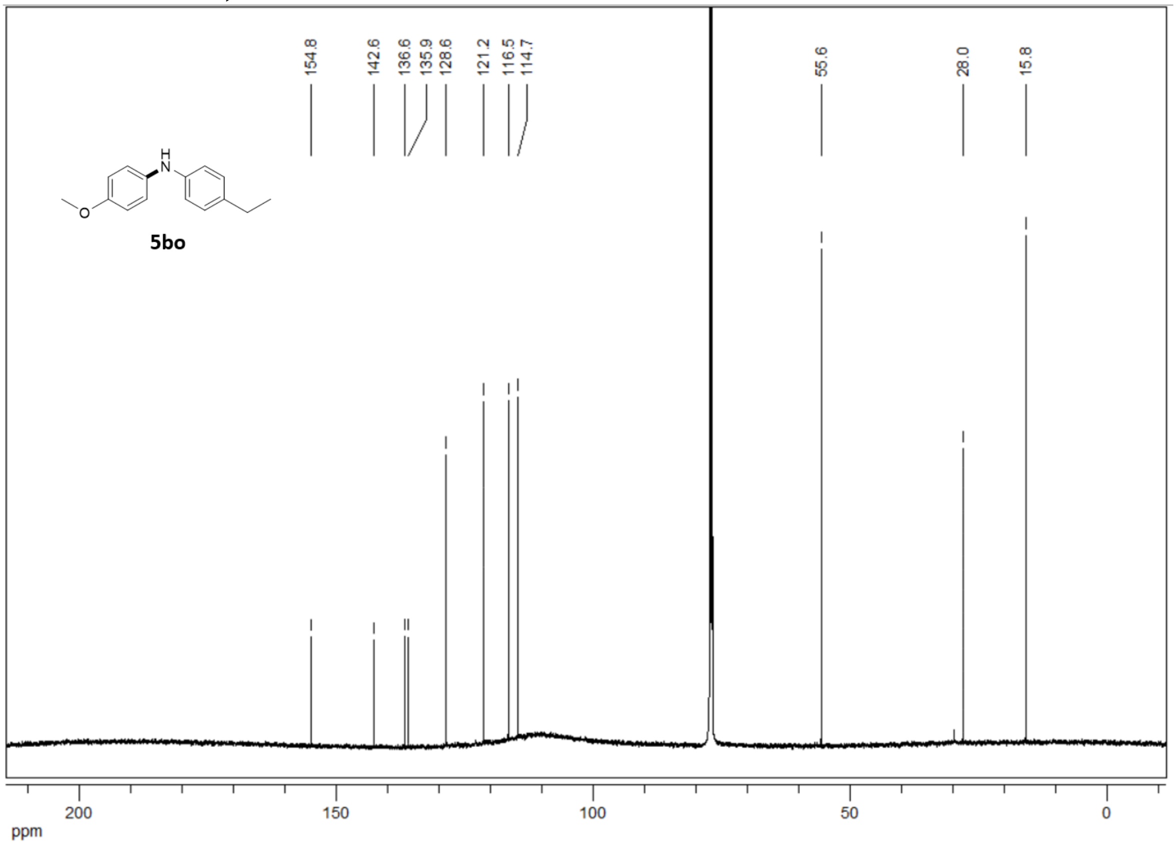

^{1}H NMR 600MHz, $CDCl_3$

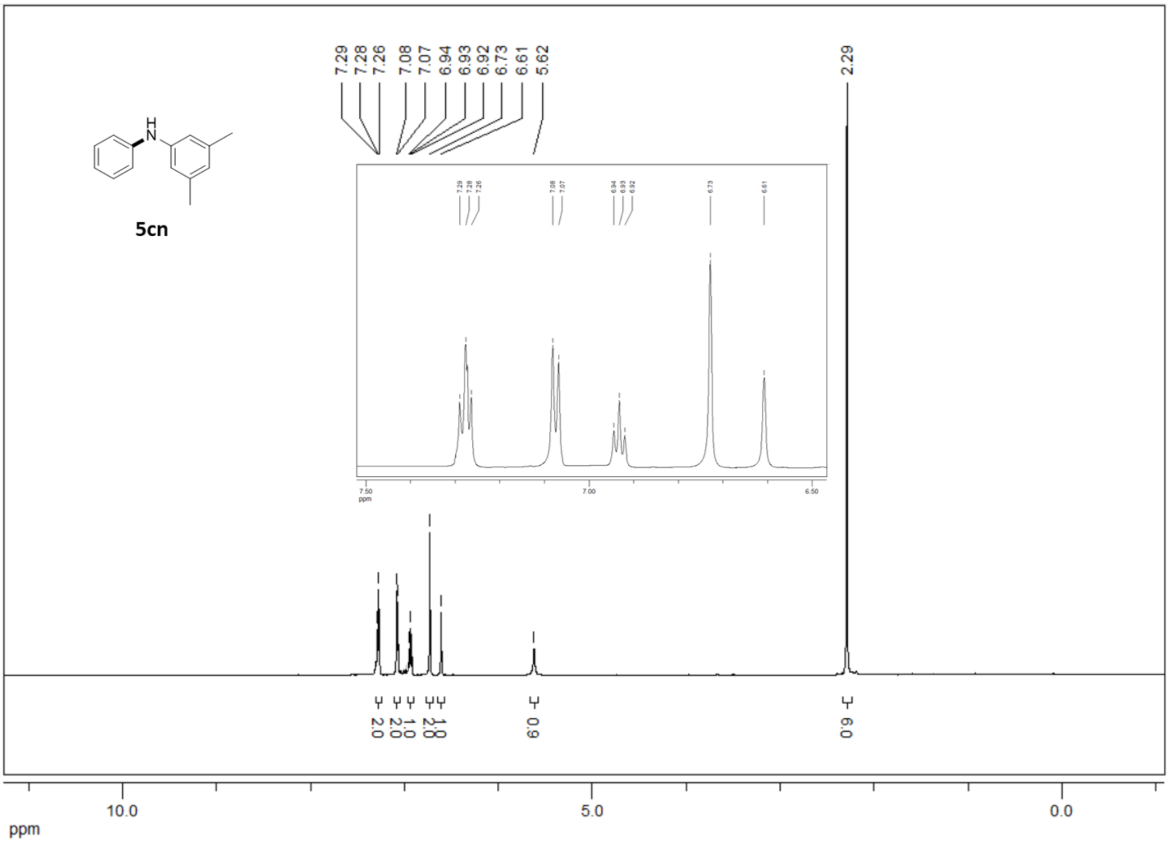

^{13}C NMR 150MHz, $CDCl_3$

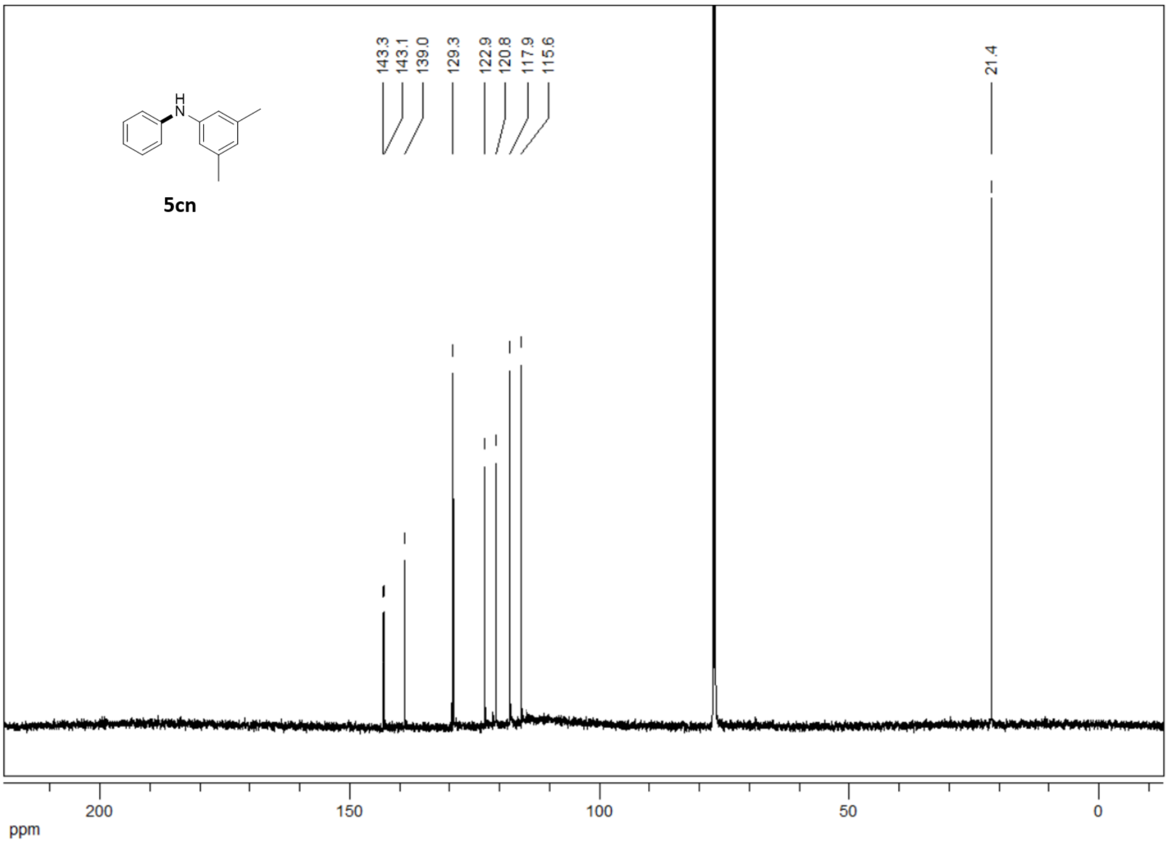

Supplement: Supplementary file 1 [file Data_Sheet_1.PDF]
